# Clickable Polymer-Based Coatings for Modulating the Interaction of Metal–Organic Framework Nanocrystals with Living Cells

**DOI:** 10.1021/acsami.5c01695

**Published:** 2025-04-21

**Authors:** Manuela Cedrún-Morales, Martina Migliavacca, Manuel Ceballos, Marta Perez-Maseda, Giulia Zampini, María Teresa Alameda Felgueiras, Jon Ostolaza-Paraiso, Marisa Juanes, Irene Rincón, David Fairen-Jimenez, Javier Montenegro, Patricia Horcajada, Ester Polo, Beatriz Pelaz, Pablo del Pino

**Affiliations:** † Centro Singular de Investigación en Química Biolóxica e Materiais Moleculares (CiQUS), Departamento de Física de Partículas, 16780 Universidade de Santiago de Compostela, 15705 Santiago de Compostela, Spain; ‡ Centro Singular de Investigación en Química Biolóxica e Materiais Moleculares (CiQUS), Departamento de Bioquímica y Biología Molecular, Universidade de Santiago de Compostela, 15705 Santiago de Compostela, Spain; § Centro Singular de Investigación en Química Biolóxica e Materiais Moleculares (CiQUS), Departamento de Química Inorgánica, Universidade de Santiago de Compostela, 15705 Santiago de Compostela, Spain; ∥ The Adsorption and Advanced Materials Laboratory (A2ML), Department of Chemical Engineering and Biotechnology, 2152 University of Cambridge, Philippa Fawcett Drive, Cambridge CB3 0AS, U.K.; ⊥ Centro Singular de Investigación en Química Biolóxica e Materiais Moleculares (CiQUS), Departamento de Química Orgánica, Universidade de Santiago de Compostela, 15705 Santiago de Compostela, Spain; # Advanced Porous Materials Unit (APMU), 202532 IMDEA Energy Institute, Av. Ramón de la Sagra 3, 28935 Móstoles-Madrid, Spain

**Keywords:** metal–organic-frameworks, click chemistry, polymer coating, functionalization, nanocrystal-cell interactions

## Abstract

Nanosized microporous metal–organic-frameworks (NMOFs) serve as versatile drug delivery systems capable of navigating complex microenvironments and interacting with cells in specific tissues. The physicochemical properties of NMOFs, such as size, composition, porosity, colloidal stability, and external surface functionalization are essential for their success as efficient carriers. This study introduces a flexible, clickable coating using an amphiphilic polymer derivatized with dibenzo cyclooctyne groups as a universal, postsynthetic functionalization tool. To prove its universality, nanosized MOFs with different structure and composition (UiO-67, NU-1000, PCN-222, and ZIF-8) were produced with high monodispersity and were coated with a clickable, amphiphilic polymer. The resulting polymer-coated NMOFs display exceptional colloidal and structural stability in different biologically relevant media. For comparative purposes, we selected two size-equivalent NMOFs, ZIF-8 and UiO-67, which were functionalized with a library of biologically relevant azide-derivatized (macro)­molecules, including poly­(ethylene glycol), mannose, and a dynein-binding cell-penetrating peptide, using a bioorthogonal reaction. The choice of ZIF-8 and UiO-67, both 150 nm in size but with distinct coordination and surface chemistries, is pivotal due to their differing acid and base stability characteristics, which may potentially influence their performance in cellular environments. To track their performance *in vitro*, the NMOFs were loaded with cresyl violet, a common histological stain and lysosomal marker. Cellular internalization of the surface-functionalized NMOFs was markedly governed by their distinct (macro)­molecule characteristics. This demonstrates that surface properties critically influence uptake efficiency, while also highlighting the versatility and effectiveness of the proposed coating strategy. In particular, the one functionalized with the dynein-binding peptide demonstrated a markedly higher rate of cellular internalization compared to other NMOFs. In contrast, derivatizations with mannose and poly­(ethylene glycol) are associated with a substantial reduction in cellular uptake, suggesting stealth behavior. These results provide a bioorthogonal and versatile alternative for the external surface engineering of NMOFs, aiming to improve targeted drug delivery effectiveness.

## Graphical abstract

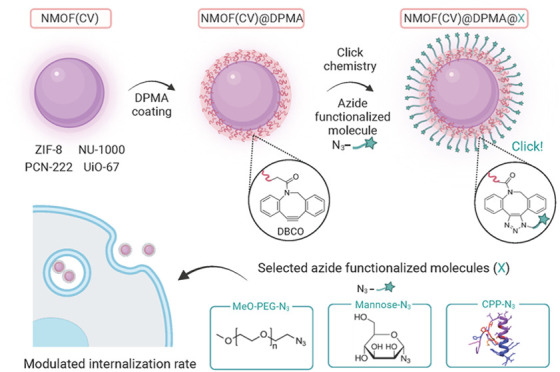

## 1 Introduction

Metal–organic-frameworks (MOFs) constitute a prolific family of porous materials consisting of three-dimensional (3D) extended networks that comprise metallic centers coordinated to polycomplexant organic ligands.^1^ These materials have gained significant interest due to their unique physicochemical properties, arising from their ordered voids with a defined size and chemical environment, as designed by their constituents. MOFs have been extensively explored for various applications, such as catalysis, gas storage, and sensing.^2^ Due to their large porosities, biocompatibility, and well-defined tunable structures, they are considered ideal candidates for drug delivery vehicles.^3^ The highly tunable surface functionalities of MOFs and their ability to encapsulate large amounts of diverse biologically relevant molecules make them excellent candidates for overcoming some limitations in drug delivery, such as prolonged and targeted delivery to site of action or therapeutic effectiveness.^3,4^

The microporous nature of ZIF-8 has made it a popular choice for drug delivery as it can accommodate a wide range of challenging drugs and grow around macromolecules, larger than their pore size.^5^ However, the rapid cargo release kinetics associated with these NMOFs in physiological media hamper their application in biomedicine, primarily stemming from its instability in aqueous environments, which is influenced by factors such as pH and concentration.^6^ This degradation is also related to the action of dissolved carbon dioxide which can replace imidazolate linkers leading to the degradation of the nanosized ZIF-8.^7^ Alternatively, the UiO family of Zr-MOFs, which feature Zr_6_O_4_(OH)_4_^12+^ clusters as secondary building units (SBU), are known for their exceptional hydrolytic stability and high tolerance for structural defects.^8^ Among the members of this prolific family is the isoreticular UiO-67, with 4,4′-biphenyl dicarboxylate (BPDC) linkers. UiO-67 features relatively large pores (∼1 nm) and high thermal and chemical stability due to its [Zr_6_O_4_(OH)_4_(BPDC)_12_] metal–organic coordination complex, making it a high-potential candidate for biomedical applications. However, conflicting conclusions about UiO-67′s stability against exposure to aqueous settings have been reported in multiple articles,^9^ as well as apparently contradictory findings for other Zr_6_(OH)_4_O_4_^12+^-based MOFs, including NU-1000, PCN-222 and MOF-808.^10–12^

To use MOF nanocarriers in healthcare, and thus in physiological environments, they must structurally and chemically withstand the relatively high endogenous phosphate concentrations (1–10 mM) and various pH present in biological settings. This is important to ensure they remain stable enough to deliver their payloads effectively, while also being biodegradable to prevent bioaccumulation and potential toxicity in the body. This may pose challenges, particularly for Zr-, Hf- and Ti-based MOFs^13^ due to the strong coordinating nature of phosphate ions.^8,14^ Also, NMOFs should remain colloidally stable in the presence of crowded media such as blood, serum, or cytosolic media of cells.^8,15^

Due to the need of ensuring NMOFs’ stability and acquiring recognition abilities, one of the primary interests of NMOFs in the context of biological applications is their potential for external surface functionalization.^4,15–17^ Two main strategies are commonly used: modification during self-assembly and postsynthetic modification (PSM). While self-assembly offers some control over surface properties, it is limited by the chemical diversity of available linkers and potential impacts on MOF stability. PSM, however, provides greater flexibility, allowing the functionalization of presynthesized NMOFs while preserving their porosity and crystallinity. The main PSM methods include coordinative PSM, that employs free metal sites on NMOFs to attach new functionalities via coordination chemistry; covalent PSM, that relies on the creation of stable bonds with the NMOF surface to introduce external functionalities, and core–shell strategies, based on the coating NMOFs with silica or polymers to improve their stability, prevent premature drug release, and allow further functionalization. This last option offers the possibility of creating universal surfaces containing further reactive groups which allows the generation of equivalent surfaces for different NMOFs.

PSM can improve colloidal stability, cellular uptake, drug release control, or prolong circulation time, among others.^15,18^ Furthermore, active targeting through specific interactions between homing molecules such as peptides, antibodies, or saccharides can enhance the performance of these systems.^15,19^ Polymers such as poly­(ethylene glycol) (PEG) stand out as one of the most utilized macromolecules for stabilizing nanomaterials. PEG’s widespread use is attributed to its antifouling properties, which partially prevent opsonization and uptake by macrophages, thereby promoting prolonged blood circulation.^20–22^ On the other hand, carbohydrates are also extensively employed as biomolecular coatings, garnering attention for their capacity to enhance cell recognition, stability, and biocompatibility.^23,24^ Among carbohydrate-functionalized nanomaterials, mannose-conjugated nanoparticles provide new advances in targeted delivery because lectins with mannose-binding ability are predominantly expressed on various cancer cells, enabling cancer treatment targeting.^25,26^ Mannose is a relevant carbohydrate capable of binding to glycolipids and glycoproteins, playing a crucial role in cell–cell recognition and enzyme-triggering processes.^27^ External surface functionalization of nanoparticles with mannose molecules can enhance target binding and selectivity of drug delivery systems (DDSs), as several tumor cell lines overexpress the mannose receptor (MR).^25,28^ Studies have shown that mannose can affect tumor growth. As tumor cells grow, their metabolic demands exceed their energy supply, with saccharides being the primary source of energy. Recent research has reported that mannose can inhibit tumor growth and improve response to chemotherapy in solid tumors.^26^

Moreover, mannose can be used as a tumor-targeting ligand due to the high expression of the mannose receptor in certain lung cancer cell lines, such as A549 cells. The mannose-derived nanocarrier can selectively enter mannose receptor-rich cells through mannose receptor-mediated endocytosis.^29^

Similarly, cell-penetrating peptides (CPPs) are widely used as a biomimetic, virus-inspired approach for delivering a broad range of cargoes across cellular membranes.^30,31^ CPPs belong to a class of peptides that have the potential to act as carriers for covalently or noncovalently attached cargoes of various sizes, ranging from small molecules to peptides, nucleic acids, proteins, and even nanoparticles.^32^ These peptides facilitate the translocation of certain (macro)­molecules across the plasma membrane, enabling them to overcome cellular transport mechanisms such as endocytosis, which can impede the cytosolic delivery of hydrophilic particles or molecules with high molecular weight.^32–34^ CPPs find applications in diverse fields, including gene delivery, liposome functionalization, nanoparticle decoration, and therapeutic protein delivery.^31,35^

Among (PSM) methods, covalent PSM involves external surface functionalization by forming covalent bonds between selected functional groups and the partially coordinated linkers placed on the outer surface of the NMOFs. This method includes various covalent strategies, including *N*-alkylation, metal coordination, click chemistry, and protonation. The grafting of oligonucleotides onto the external surface of NMOFs has been achieved by coordinating a phosphate group to unsaturated metal sites of the MOF.^36^ A similar approach was employed using PEG,^22^ which was further developed into the formation of a bilayer to protect the MOF carrier and regulate drug release.^37^ In any case, click chemistry stands out as a series of chemical reactions offering distinct advantages over traditional methods,^38–40^ including high yields, broad applicability, minimal generation of cytotoxic byproducts, high stereospecificity, and straightforward reaction conditions.^41^ Additionally, it exhibits compatibility with a range of functional groups such as azides, amines, and carboxylic acids, rendering it a versatile tool for the surface modification of NMOFs in biomedical applications.

Compared to conventional methods like direct ligand coordination, which can be reversible and sensitive to physiological conditions, click-chemistry functionalization forms strong covalent bonds, enhancing thermal and chemical stability. Furthermore, these modifications allow for a higher functionalization density due to the high reaction efficiency and the possibility of modifying specific sites without compromising the structural integrity of the NMOF.^42^ In contrast, conventional functionalization may be limited by steric hindrance and competition for metal coordination sites, reducing the effective number of incorporated ligands. Additionally, this method for surface modification allows more controlled biological interactions, as the selectivity of the reaction enables the introduction of specific functional molecules, such as peptides or sugars, with precise orientation. In particular, the copper-free variant of the azide–alkyne Huisgen cycloaddition involves the reaction between a diaryl cyclooctyne moiety, such as dibenzo cyclooctyne (DBCO) or azadibenzocyclooctyne (ADIBO), and an azide-labeled reaction partner.^43^ This reaction, known as strain-promoted alkyne azide cycloaddition (SPAAC), offers significant advantages.^44^

Notably, these copper-free reactions exhibit rapid kinetics at room temperature (RT) and can be conducted within living cells without inducing cytotoxicity associated with copper ions. This is particularly advantageous, as traditional Cu­(I)-catalyzed reactions may cause damage to normal cells and healthy tissues.^45^ However, despite its advantages, click chemistry also presents certain drawbacks, particularly the requirement for ligand modification to incorporate the necessary functional groups, a process that may not be universally applicable across all types of NMOFs.^15^ To address this challenge, we propose here a hybrid approach that combines the strengths of both strategies. Specifically, we propose utilizing the amphiphilic polymer poly-[isobutylene-*alt*-maleic anhydride]-*graft*-dodecyl (PMA), which was further derivatized with DBCO groups (DPMA) serving as anchor points for the covalent attachment of selected biomolecules via click chemistry. This approach would improve the application of click chemistry alone and it would also expand the scope of surface functionalization possibilities for NMOFs in biomedical applications (see Table S1).

Here, we report the synthesis of highly monodispersed Zr- and Zn-based NMOFs – PCN-222, NU-1000, UiO-67, and ZIF-8 – and the suitability of the polymer coating technique for these NMOFs as a universal postsynthetic modification (Scheme 1). Colloidal, structural, and chemical stability in simulated biological media were significantly improved in all cases upon application of the polymer coating, while *in vitro* toxicity was reduced. Upon DPMA coating, the NMOFs exhibited enhanced colloidal stability in various biologically relevant media and enabled rapid surface functionalization through click chemistry. Then, focusing on size equivalent ZIF-8 and UiO-67 NMOFs, we prove the versatility of the generated surface by the conjugation of different bioactive molecules containing azide groups. Both systems were loaded with cresyl violet (CV), DPMA-coated, and subsequently functionalized on the surface via click chemistry with azide-containing molecules: poly­(ethylene glycol)-azide (PEG-N_3_, 5 kDa), mannose-azide (Man-N_3_) and a cell-penetrating peptide-azide (CPP-N_3_). This approach revealed that the NMOFs were efficiently internalized by living cells without impairing cell viability and with a clear dependency on the ligands exposed on their surfaces.

**Scheme 1:**
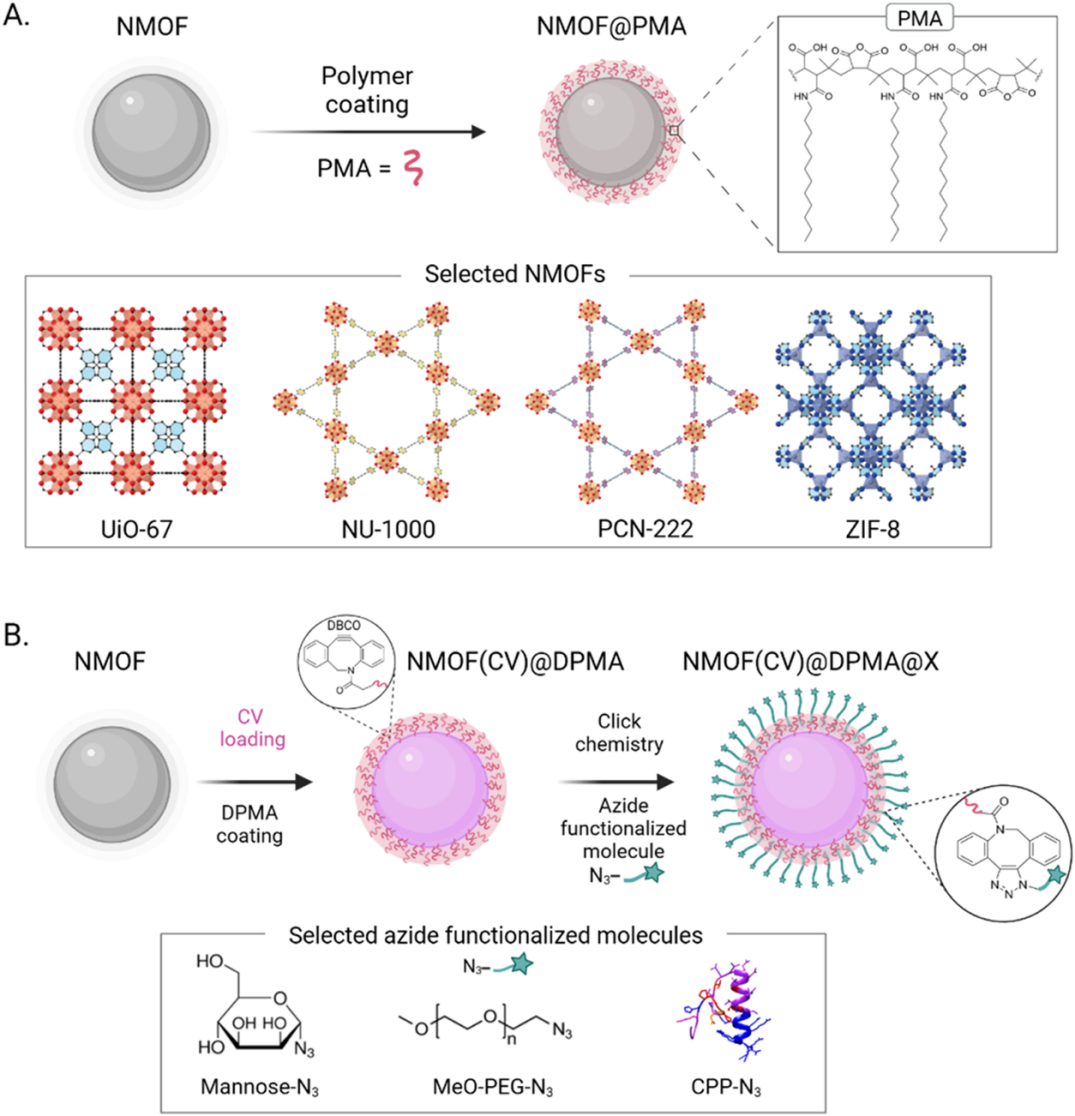
(A) Synthesis and Polymer Coating of NMOFs and Crystal Structures of the Selected Systems. (B) Cargo Encapsulation and Click Chemistry Functionalization Scheme, with the Corresponding (Macro)­molecules Employed for the Process

## 2 Results and Discussion

### 2.1 Synthesis and Surface Functionalization

Controlling size and morphology while producing nanoparticles is critical for their application in biological environments. Therefore, we optimized some of our previously reported methods to produce UiO-67 and ZIF-8 NMOFs,^20,46^ as well as other Zr-based NMOFs such as NU-1000^46^ and PCN-222 ^47^ (Figure S1). Typical syntheses to produce Zr-based MOFs are based on solvothermal methods employing high boiling temperature solvents such as DMF or DMSO to dissolve the metal salt(s) and ligand(s) in a vial or closed vessel, allowing for heating during long periods (i.e., 12 h or longer). Generally, organic acids such as acetic acid (AA), formic acid (FA), trifluoroacetic acid (TFA), and benzoic acid (BA), are used as modulators to control the reaction kinetics and, thus, the particle size. These modulators compete with the organic linkers for the available Zr sites, thereby controlling the growth of the particles.^48^

In our case, for the synthesis of the selected Zr-MOFs we have employed modifications of the experimental protocol described in Ceballos et al. where DMF was used as a solvent and AA as modulator,^1^ except for PCN-222 for which we used TFA as modulator and a preformed Zr-cluster.^20,46^ To produce UiO-67, ZrOCl_2_·8H_2_O, H_2_BPDC, and AA were used as metal precursor, ligand, and modulator, respectively, using a molar ratio of 1:2:220, as described in the Section 4. The resulting UiO-67 nanoparticles were characterized using scanning electron microscopy (SEM), presenting an octahedral morphology with an average edge size of 141 ± 4 nm (Figures 1a and S2). Dynamic light scattering (DLS) and ζ-potential measurements showed an average hydrodynamic diameter (*d*_h_) of 144 ± 11 nm, and an external surface charge of 15 ± 6 mV, respectively (Figure 2a,b and Table S2).

**Figure 1:**
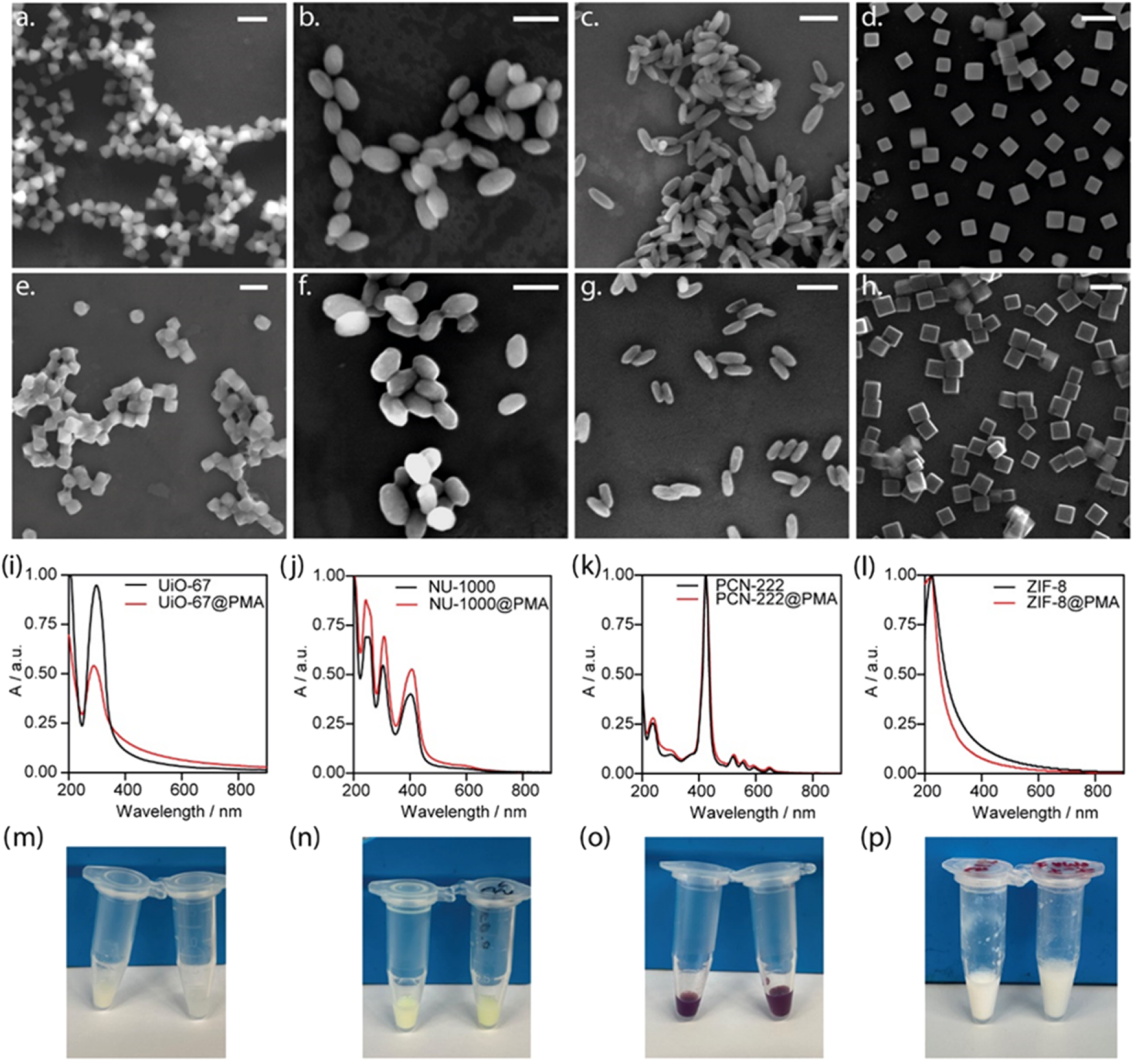
SEM images of the as-synthesized NMOFs (a) UiO-67, (b) NU-1000, (c) PCN-222 and (d) ZIF-8, and PMA-coated NMOFs redispersed in Milli-Q water, (e) UiO-67@PMA, (f) NU-1000@PMA, (g) PCN-222@PMA and (h) ZIF-8@PMA. Normalized UV–vis spectra corresponding to the above NMOFs before and after the polymer coating (i) UiO-67, (j) NU-1000, (k) PCN-222 and (l) ZIF-8. Photographs of NMOFs solutions before (left) and after (right) PMA coating of (m) UiO-67, (n) NU-1000, (o) PCN-222 and (p) ZIF-8. Scale bars are 200 nm.

**Figure 2:**
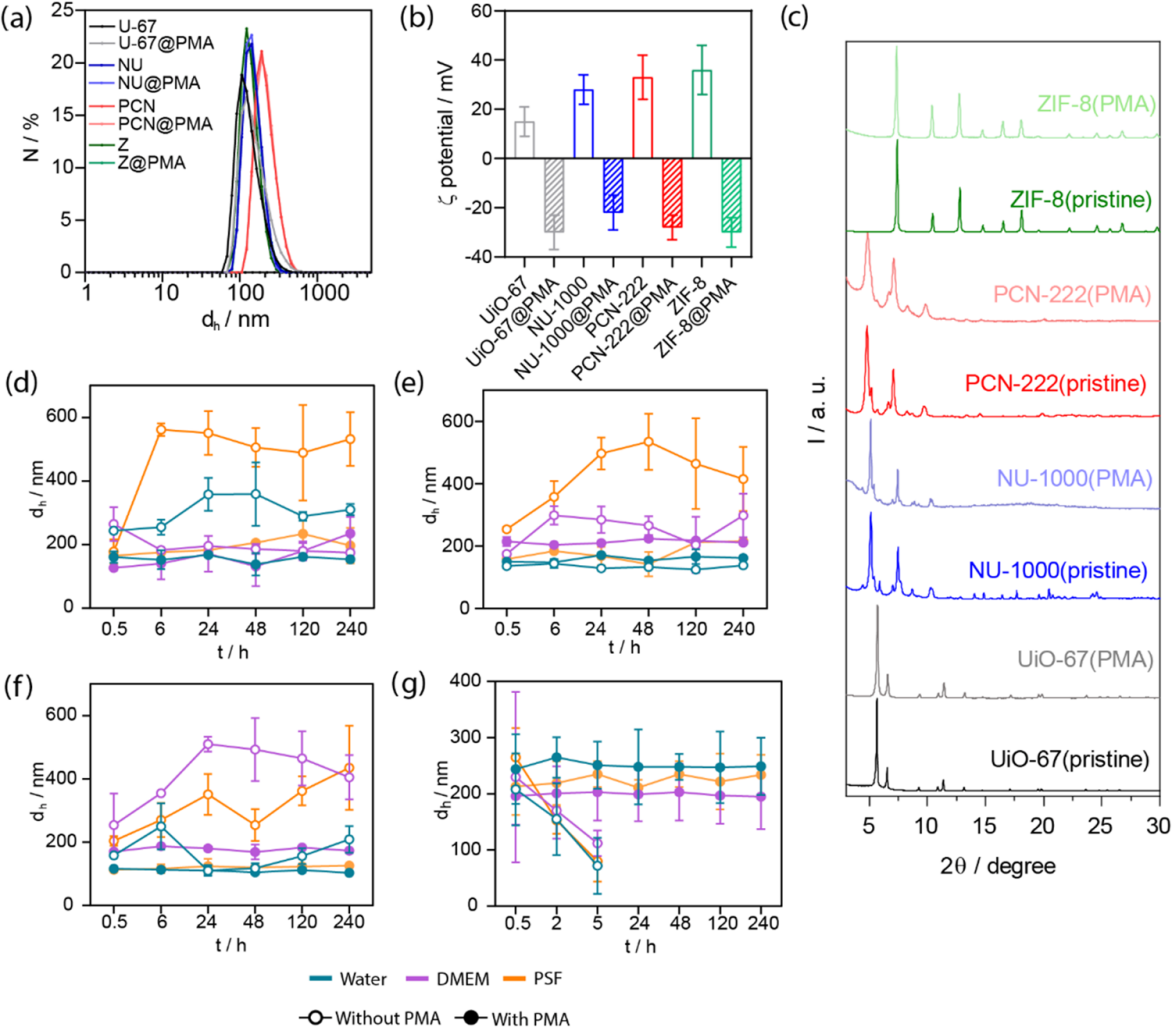
(a) Hydrodynamic diameter *d*_h_ measurement of the MOFs before and after the PMA coating (U = UiO-67, NU = NU-1000, PCN = PCN-222, Z = ZIF-8), (b) ζ-potential of MOFs before and after PMA functionalization and (c) PXRD patterns of the samples. Colloidal stability based on the hydrodynamic diameter of (d) UiO-67, (e) NU-1000, (f) PCN-222 and (g) ZIF-8 over time in water (blue), DMEM (complete cell media, violet) and phagolysosomal simulant fluid (PSF, orange) media, as determined by DLS (solid dots with PMA and empty dots without PMA). All experiments were conducted in triplicate. Error bars represent the standard deviation of the mean average *d*_h_.

For NU-1000, ZrOCl_2_·8H_2_O, 4,4′,4″,4‴-(pyrene-1,3,6,8-tetrayl)­tetrabenzoic acid (TBAPy), and AA were used as metal precursor, ligand, and modulator, respectively, using a molar ratio of 13.5:1:2.4. The resulting NU-1000 NMOFs were characterized using SEM, presenting an ellipsoidal morphology with an average length of 151 ± 16 nm (Figures 1b and S3). DLS and ζ-potential measurements showed a *d*_h_ of 150 ± 12 nm and an external surface charge of 28 ± 6 mV, respectively. PCN-222 was prepared in a DMF solution using previously synthesized Zr_6_O_4_(OH)_4_(benzoate)_12_ clusters and tetrakis (4-carboxyphenyl) porphyrin (TCPP) as metal precursor and linker, respectively,^20,49^ while TFA was used as the modulator. The resulting PCN-222 NMOFs presented ellipsoidal morphology with an average length of 134 ± 13 nm (Figures 1c and S4). DLS and ζ-potential measurements showed a *d*_h_ of 145 ± 10 nm and an external surface charge of 33 ± 9 mV, respectively.

ZIF-8 NMOFs were prepared following a previously reported method, in water at RT using Zn­(NO_3_)_2_·6H_2_O and 2-methylimidazole (MeIm) as the zinc and organic ligand sources, respectively, with a molar ratio of ligand to zinc ion (MeIm/Zn^2+^) of 54. Cetyltrimethylammonium bromide (CTAB) was used as size-controlling and structural-directing agent, resulting in the characteristic cuboidal shape with an average diameter size of 140 ± 8 nm (Figures 1d and S5) and an external surface charge of 36 ± 10 mV.

To study the effect of the external surface functionalization of the NMOFs, we selected the amphiphilic polymer PMA. This polymer has been used to stabilize inorganic nanoparticles of different composition, shapes, and sizes.^50^ PMA was already studied as a stabilizing and capping agent to ensure the stability of the loaded cargo inside NMOFs voids.^6,51,52^ This step is crucial due to the fast degradation of ZIF-8 when exposed to pH < 7 and CO_2_ dissolved in water, a process that typically unfolds within minutes.^7^ After the PMA functionalization and redispersion in Milli-Q water, the purified NMOFs (NMOF@PMA) did not present any changes in the morphology nor the sizes measured by SEM and DLS, respectively (Figures 1e–h and 2a, and Table S2). ultraviolet–visible (UV–vis) spectra revealed no significant optical differences among the samples before and after the functionalization (Figure 1i–l), indicating that the integrity of the NMOFs is preserved, as well as the sample appearance before and after the PMA functionalization. This was further corroborated by visual inspection of the NMOF solutions, as shown in Figure 1m–p. In contrast, ζ-potential values revealed a clear decrease among all the samples, which become highly negative (around −30 mV in all cases) due to the negative character of the PMA, associated with the ionization of −COOH groups present before and after the hydrolysis of maleic anhydride rings of PMA (Figure 2b and Table S2). This fall in the ζ-potential values (indicating charge inversion) indicated the successful PMA functionalization of the NMOFs particles.

The structural stability of the studied NMOFs was evaluated by powder X-ray diffraction (PXRD). Before the PMA coating, the pristine materials UiO-67, NU-1000, PCN-222, and ZIF-8 exhibited well-defined diffraction peaks (Figure 2c). Unit cells were refined by Pawley’s refinement, where UiO-67 showed a cubic structure with a cell parameter of 26.9635 Å and a space group *Fm*3 ®*m*, while ZIF-8 exhibited a cubic structure with a cell parameter of 16.9417 Å and a space group *I*4 ®3*m*. Both NU-1000 and PCN-222 displayed hexagonal structures with a space group *P*6/*mmm* and cell parameters of *a* = 39.8204 Å, *c* = 16.7904 Å for NU-1000, and *a* = 42.5744 Å, *c* = 17.0712 Å for PCN-222 (Figure S6). Notably, the synthesis of NMOFs in the NU-1000 phase can occasionally produce an undesired byproduct, the polymorph NU-901, which has a smaller average pore size and pore volume compared to NU-1000.^53^ In our case, this was confirmed by Pawley refinement (Figure S6b).

After PMA coating, the crystal structure was preserved as shown in Figure 2c. This is a significant benefit for some NMOFs such as ZIF-8, which present poor water stability due to the presence of CO_2_ dissolved in water damaging the MOF structure by forming ZnCO_3_ and causing the structures to collapse.^7^ This preservation also benefits MOFs with large pores such as UiO-67,^9^ as well as other Zr-MOFs, including NU-1000 and PCN-222, which have open metal sites that can be occupied by water molecules or phosphate groups due to the exposure of very strong Lewis acid Zr-sites.^54,55^

To quantify the weight percentage (% wt) of PMA in the NMOFs, thermogravimetric analysis (TGA) was performed (Figure S7), assessing their thermal behavior. All of them were thermally stable up to 400 °C. The thermograms were normalized to 100% based on their corresponding inorganic residue (ZrO_2_ or ZnO) to determine the connectivity of each NMOF (Figure S7), following the method reported by Valenzano.^56^ The solid lines represent the molar mass of the Zr_6_O_4_(OH)_4_ cluster with its corresponding number of linkers for UiO-67, NU-1000, and PCN-222; while the dashed lines indicate the dehydrated Zr-oxo cluster resulting from the loss of two water molecules and the change from an 8-coordinated square antiprismatic to a distorted 7-coordinated monocapped trigonal prismatic geometry (Figure S7).^57^ For ZIF-8, the solid lines represent a Zn node with two MeIm linkers due to its bidentate nature.

For UiO-67, NU-1000, and PCN-222, a final plateau representing the ZrO_2_ residue left from the temperature treatment is observed. The residual mass % for UiO-67 in this case is 35% whereas for samples with PMA, the inorganic composition is reduced to 19%, indicating that a 16% of the total weight of the sample corresponds to the polymer (Figure S7a,b). The presence of PMA is indicated by a significant weight loss before 400 °C. Specifically, an initial mass loss of 10 wt % is attributed to CO_2_ release from the −COOH groups of PMA, occurring between 150–250 °C, and a final loss of 20 wt % at around 250–350 °C. For NU-1000, it shows a residual mass of 27% and 9% for the samples without and with PMA, respectively (Figure S7c,d). Therefore, the polymer % corresponds to 18%, similar to that of UiO-67. For PCN-222, noncoated MOFs presented an inorganic residue of 23% whereas the PMA functionalized ones have a 7%, meaning a polymer content of 16% (Figure S7e,f).

Figure S7a shows the normalized thermogram for UiO-67, where a plateau between 400 and 500 °C is observed, indicating the presence of missing linker defects. Figure S7c presents the opposite case for NU-1000, where the theoretical line represents an 8-connected Zr-oxo cluster. This could be attributed to an excess of metal during synthesis, which can induce the formation of missing-cluster defects.^58^ Figure S7e shows a defect-free PCN-222 with an 8-connected Zr-oxo cluster, or two TCPP molecules per cluster. Similarly, ZIF-8 (Figure S7g) does not present missing linker defects. It is important to highlight that missing linkers or the presence of cluster defects are often associated with a decline in the chemical, thermal, and structural stability of the MOF.^59^

Colloidal stability in biological media is a key factor influencing the final fate of nanomaterials. We assessed the colloidal stability in different relevant biological media, including water, complete cell media supplemented with serum (Dulbecco’s Modified Eagle Medium with 10% FBS), and phagolysosome simulated fluid (PSF, pH 5) over 10 days using DLS (Figure 2d–g, and Tables S3–S6). Specifically, PSF simulates the lysosomal environment postinternalization, containing elevated levels of salts and high phosphate content that may bind to the Zr cluster or Zn center, leading to the degradation of the structures.^46^ Stability assays demonstrated improved stability over time for PMA-coated UiO-67, particularly in cell media, where a slight increase in size was observed, possibly due to nonspecific protein adsorption,^60^ yet the size remained stable over the studied period. Similarly, NU-1000 and PCN-222 exhibited higher colloidal stability after functionalization. On the other hand, uncoated NMOFs tended to aggregate, attributed to their high surface area at the nanoscale, which attempts to minimize their surface energies especially in PSF and cell media, where a clear increase in particle size was observed after 6 h (Figure 2e,f). In contrast, for uncoated ZIF-8 (Figure 2g), a decrease in the hydrodynamic diameter was observed after just 5 h in both cell media and PSF, indicative of poor stability and structural degradation in aqueous media and acidic environments.^6^ After PMA coating, all the samples exhibited excellent colloidal stability for up to 10 days in all tested media (Figure 2d–g).

Furthermore, SEM analyses were conducted to assess morphological changes. As in previous colloidal stability tests, PSF medium was chosen to mimic the biological environment postcellular internalization. The Zr NMOFs (PMA-coated and uncoated) were maintained in PSF for 7 days, then washed and prepared for SEM analysis. The images revealed no significant variations in the structural appearance after 7 days (Figure S8). However, as anticipated from DLS measurements, the ZIF-8 structure showed clear morphological degradation, while the ZIF-8@PMA exhibited no morphological changes or apparent degradation (Figure S8d,h).

To complement the stability tests, UiO-67 was specifically chosen to assess the release of its constitutive linker from the bare NMOFs. The presence of the linker in the supernatant (SN) was measured at different time points in water and PSF using high performance liquid chromatography (HPLC). Figure S9 illustrates the degradation patterns observed in both media at 0 h, 1, and 7 days. The findings demonstrate a higher degree of stability in PSF compared to water. This difference in structural stability can be attributed to the increased acidic character of PSF (pH 5), which generally favors the stability of carboxylate-based MOFs within a pH range around 3–5. Additionally, the presence of PMA significantly enhances stability in both media. The polymer coating on the NMOF surfaces acts as a protective barrier, effectively mitigating premature degradation in both media.

### 2.2 Cresyl Violet Loading, Stability and Leakage Assays

After proving the efficiency and universality of the PMA surface functionalization approach, we studied the encapsulation and loading capabilities of each NMOF in combination with the PMA coating. As a model, we selected the fluorescent molecule cresyl violet (CV) (Figure S10) a well-known fluorescent dye often used as a lysosomal marker in molecular biology.^61^ Additionally, the toxicity associated with CV at concentrations exceeding 100 μM serves as an indicator of any potential leakage from the loaded NMOFs. The harsh synthesis conditions of Zr-NMOFs hindered the *in situ* encapsulation of certain biomolecules such as proteins. Nonetheless, the selected postsynthesis loading method proves highly efficient and water-compatible, offering mild loading conditions for various biomolecules such as proteins or nucleic acids.^62^ In contrast, in ZIF-8 the encapsulation of CV (ZIF-8­(CV)) was achieved by incorporating the dye during the synthesis (see Section 4).

The postsynthetic encapsulation of CV consists on the immersion of the NMOFs in an aqueous CV solution for 1 day under stirring, utilizing a MOF to CV weight ratio of 1:3, as detailed in the Section 4. For UiO-67, where the dimensions of the CV molecule (12 × 4 Å) exceed the pore aperture of the MOF (∼8 Å), loading was performed at 60 °C, following the protocol outlined by Morabito et al.^63^ Conversely, for PCN-222 and NU-1000, encapsulation was conducted at RT owing to their larger window apertures, enabling direct immersion of CV in the pores of the MOFs.^21^

As mentioned above, ZIF-8­(CV) were loaded during the synthesis, and exhibited similar morphology and size to the bare ones, with an average *d*_h_ of 137 ± 7 nm (Figure 3a). This *in situ* encapsulation method enhances (i) the trapping of the loaded molecule inside the MOF while minimizing CV leaching; and (ii) it enables the encapsulation of molecules larger than the pore aperture, expanding its applicability. Considering that CV’s dimensions exceed ZIF-8′s pore aperture of 3.4 Å, this approach proves suitable for accommodating guest biomolecules such as enzymes and relatively large drugs for various bioapplications.^63^ However, its applicability is subjected to the synthetic MOF′s conditions; for instance, certain Zr-MOFs may need parameters of temperature, pH, and/or the use of some ligand/metal precursors that could compromise the integrity of selected cargoes. On the contrary, ZIF-8 is very well suited for biomolecule encapsulation due to its biocompatible synthetic conditions (i.e., RT, mild pH).^64^

**Figure 3:**
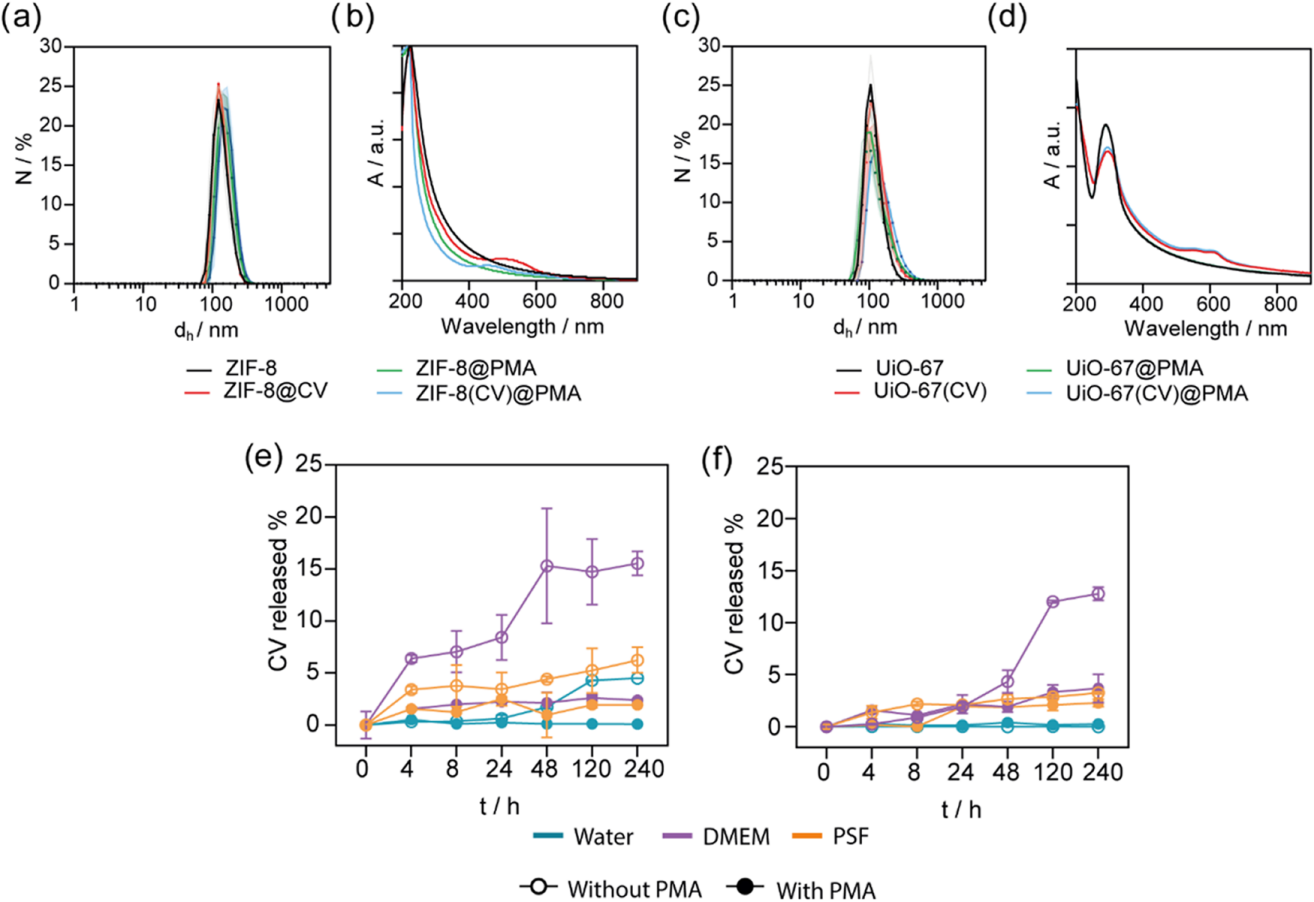
(a) *d*_h_ measurement of the ZIF-8 samples before and after the CV encapsulation and PMA functionalization and (b) UV–vis spectra. (c) *d*_h_ of UiO-67 samples before and after the CV encapsulation and PMA functionalization and (d) the corresponding UV–vis spectra. % CV released with respect to the loaded quantity measured by fluorescent measurements of the supernatant (SN) in water (blue), cell media (violet) and PSF (orange) up to 10 days for (e) ZIF-8 and (f) UiO-67 with (solid dots) and without PMA (empty dots).

The CV-loaded NMOFs were further functionalized with PMA following the protocol optimized for bare NMOFs. After purification, the NMOFs did not present any significant changes in the *d*_h_ or the morphology, as confirmed by DLS and SEM analysis, respectively (Figures 3a,c, and S11, and Table S7). Figure 3 illustrates the results obtained for UiO-67 and ZIF-8, highlighting these two NMOFs for subsequent investigations as they present different SBUs with full connectivity (NU-1000 and PCN-222 present 8-connected SBUs). Absorption spectra analysis reveals consistent profiles between pre- and postfunctionalization samples. Notably, a clear peak around λ ≈ 590 nm, characteristic of CV, appears in all CV-loaded samples, albeit with varying intensity levels (Figures 3b,d, S12 and S13) due to different CV loading rates (<3 wt %).

The encapsulated CV was quantified by measuring the fluorescence in the SN after the washing steps (see Section 4). The amount of encapsulated CV in each NMOF was determined using proper calibration curves in MeOH and water (Table 1 and Figure S14). ZIF-8 had an average of 1.73 × 10^5^ ± 4.34 × 10^2^ molecules of CV per ZIF-8 (which represents an 11.5 ± 0.7 wt %), whereas this quantity decreased to 8.1 ± 0.5 wt % after PMA coating. UiO-67 had 6.68 × 10^5^ ± 2.34 × 10^2^ molecules of CV per UiO-67 (21.4 ± 0.9 wt %), while this number decreased to 18.3 ± 0.5 wt % after the polymer coating. This reduction suggests that the aliphatic pendant chains of the polymer may infiltrate the pores, displacing the CV, which is consistent with findings from previous studies.^6^

**Table 1:** CV Encapsulation Quantification Measured via Fluorescence of the SN and TGA, and *S*_BET_ Area Calculated from BET Analysis

	CV wt % (Fluo)	CV wt % (TGA)	*S*_BET_ (m^2^·g^–1^)
ZIF-8(CV)	11.5 ± 0.7	9.5	1431
ZIF-8(CV)@PMA	8.1 ± 0.5	4.9	72
UiO-67(CV)	21.4 ± 0.9	23.9	721
UiO-67(CV)@PMA	18.3 ± 0.5	3.9	39

ζ-Potential measurements of the CV-unloaded and -loaded NMOFs exhibit positive surface potential values with negligible differences (Figure S15 and Table S7).^61^ A more significant change is observed when the NMOFs are coated with PMA, resulting in negative ζ-potential in all cases, indicative of free carboxylate groups after the hydrolysis of remaining maleic anhydride rings. To further corroborate the size of the particles and measure the concentration (particles·mL^–1^), nanoparticle tracking analysis (NTA) measurements were carried out (Figure S16). In all cases, NTA results showed that size remains stable upon PMA coating in all cases.

When designing efficient DDSs and theranostic probes, it is critical to develop new systems capable of retaining the selected drug or probe until it reaches its target.^22,37,47^ The chosen coating is anticipated to block the pores of the structure, thereby mitigating cargo leakage over time. To validate this, the stability of the CV-loaded NMOFs was assessed over 10 days both before and after the PMA-coating, using different biologically relevant media, including water, PSF, DMEM, and PBS. In this case, the amount of CV encapsulated per MOF was calculated by measuring the SN fluorescence using calibration curves for each selected media (Figures S14b and S17). The results for ZIF-8 revealed a significant reduction in the uncontrolled CV release following PMA coating, particularly evident in PSF and DMEM. In these media, values of the total loaded CV decreased from 5 to 2% and from 15 to 2% of the total loaded CV, respectively (Figure 3e, and Tables S8–S9). This decrease can be attributed to the low stability of ZIF-8 in aqueous media, leading to structural degradation and subsequent cargo release. Hence, PMA coating emerges as an effective strategy for enhancing cargo stability within the structure, resulting in significantly reduced release. These findings demonstrate that PMA not only enhances nanoparticle stability but also effectively covers the pores of the NMOF structure, minimizing cargo leakage.

For UiO-67, the encapsulated CV was stable in Milli-Q water with no significant release observed in any case (Figure 3f, and Tables S10–S11). However, for PSF, DMEM and PBS, a notable release of over 5% of the encapsulated CV is observed after 10 days. Especially in DMEM, where it goes up to 13%. The UiO-67­(CV)@PMA showed reduced leaking in all cases, with a notable effect in DMEM (just 3%) in comparison to the uncoated UiO-67­(CV). Results for NU-1000 and PCN-222 exhibit a similar trend (Figure S18, and Tables S12–S15), while the initial release of noncoated particles is relatively low, the addition of the polymer significantly enhances the stability of CV.

The adsorption capacities of the differently engineered NMOFs (PMA-coated or -uncoated, CV-loaded or -unloaded) were assessed through N_2_ sorption isotherms, as illustrated in Figure S19. Figure S19a shows the sorption isotherm of ZIF-8 NMOFs. The Type I isotherm shape is preserved postloading with CV, although the uptake decreases from 413 cm^3^·g^–1^ before loading to 375 cm^3^·g^–1^ after loading (at 0.8 *P*/*P*_0_) (Figures S20–S22). Previous investigations have revealed the inherent flexibility of ZIF-8 structures, allowing them to open their windows and accommodate larger molecules than their nominal window size,^5^ typically around 3.4 Å. However, in the nonloaded PMA-coated sample, a notable decrease in uptake to 260 cm^3^·g^–1^ is observed, whereas for the CV-loaded sample, it decreases to 72 cm^3^·g^–1^. This reduction can be attributed to both the pores being partially filled with alkyl chains from PMA and the hindrance of N_2_ diffusion into the pores caused by the coating, especially at 77 K, resulting in a significant loss of adsorption capacity. UiO-67 also exhibited a Type I isotherm, showing an uptake of 386 cm^3^·g^–1^ at 0.8 *P*/*P*_0_ (Figures S19b, S23, and S24), which decreased to 248 cm^3^·g^–1^ upon CV loading and further reduction to 39 cm^3^·g^–1^ after PMA coating of the CV-loaded sample (Table 1), following a similar trend as the ZIF-8 based samples. BET area values were determined using BETSI software (Figures S20–S24), based on an extended use of Rouquerol’s criteria.^65^

PXRD was also used to study the ZIF-8 and UiO-67 NMOFs before and after CV loading and PMA coating (Figure S25). Results show the prevalence of the main Bragg peaks analogous to previous results after adding the PMA, suggesting that the selected method for CV encapsulation does not affect the crystalline structure of the MOFs. Furthermore, the PMA coating of the NMOFs­(CV) did not affect the main Bragg peaks, although peak broadening is apparent, indicating subtle changes in crystallinity or particle size distribution.

TGA was used to analyze the composition of ZIF-8­(CV) and UiO-67­(CV) (Figure S26). The analysis was conducted similarly to that for the nonloaded MOFs. The differences in inorganic residues between the UiO-67­(CV)@PMA enabled us to estimate the relative content of PMA and CV in the samples (Tables 1 and S16–S17).

### 2.3 Cellular Uptake of Functionalized NMOFs

Next, the interaction of all the selected NMOFs with cells was evaluated in 2D cell cultures of A549 adenocarcinoma cells. Note that this cell line was used as a model. To generalize the conclusions, similar experiments should be conducted using other cell lines. The biocompatibility was assessed by determining the cellular metabolic activity using the 3-[4,5-dimethylthiazol-2-yl]-2,5-diphenyltetrazolium bromide (MTT) assay. This study confirmed the high viability related to the NMOFs at different concentrations. As demonstrated, the toxicity of MOFs can indeed be influenced by the metal component within the MOF structure, as well as by other factors such as particle size, surface properties, and the potential for leaching of metal ions. In the case of ZIF-8 and UiO-67, both have been shown to exhibit relatively low toxicity in certain cell lines, with their toxicity generally being minimal under controlled conditions. In this case, the employed concentrations were based on previous results,^6,15,49,51,52^ where particles are nontoxic. We used values of up to 100 pM in all cases, corresponding to ∼20–40 μg·mL^–1^ of Zr for UiO-67, NU-1000 and PCN-222; and 40 μg·mL^–1^ of Zn for ZIF-8. Figure S27 shows the toxicity results after 24 h of incubation for each NMOF.

Unloaded UiO-67, NU-1000, and PCN-222 showed no significant toxicity within the tested concentration range after 24 h of incubation, validating the high biocompatibility of these MOFs,^21^ as Zr is a highly biocompatible metal and is efficiently excreted, making it ideal for biomedical applications. Its low toxicity is supported by studies showing an LD50 of 4.1 mg/mL for zirconyl acetate in a rat model.^66^

A significant increase in toxicity is observed in UiO-67 (CV) and ZIF-8 (CV) samples before PMA coating, particularly at concentrations larger than 10 pM (Figure S27), corresponding to approximately 0.6 μM of encapsulated CV. This increase in toxicity is attributed to the leakage of CV associated with these NMOFs, as evidenced in previous stability tests in cell media and the related cytotoxicity observed for free CV (Figure S28). However, postfunctionalization with PMA demonstrates a notable enhancement in viability, evidencing its improvement in preventing aggregation and its efficacy in impeding cargo release to the DMEM medium. In contrast, both NU-1000 and PCN-222 consistently exhibit high viability across all conditions also related to the minimal cargo release. The MTT assay confirms that the selected Zr MOFs­(CV)@PMA and ZIF-8­(CV)@PMA exhibit good biocompatibility at concentrations up to 100 pM. Therefore, the PMA-coating of NMOFs remarkably retains the loaded cargo, effectively mitigating any potential leakage.

To better understand the cargo release kinetics in cells, more viability studies were carried out using UiO-67­(CV), both with and without PMA coating at two concentrations (10 and 100 pM) and at three incubations times (24, 48, and 120 h). MTT assays confirmed that cell viability significantly decreased with UiO-76­(CV) while remaining above 80% with UiO-67­(CV)@PMA even after 120 h (Figure S29). Microscopic analysis after 120 h (Figure S30) showed a significant decrease in cell population in the uncoated structures, whereas UiO-67­(CV)@PMA maintained a cell growth comparable to the control. The observed high cell survival rate, even after extended incubation periods, underscores the potential of the developed systems for biomedical applications, highlighting the importance of ensuring that the nanocarriers and the encapsulated drug remain stable and effective until they reach the desired tissue or cell for delivery or imaging purposes.

At this point, we have addressed stability concerns in aqueous media for the proposed NMOFs, but further modifications are necessary to improve their specificity for targeted cell interactions. Therefore, we explore the covalent linkage of various relevant biomolecules via click chemistry to direct cell-NMOF interactions. For comparative analysis, we used two size-equivalent (150 nm) NMOFs, ZIF-8­(CV) and UiO-67­(CV), which were coated with DBCO-derivatized PMA (refer as DPMA). To simplify terminology, UiO-67­(CV)@DPMA and ZIF-8­(CV)@DPMA will be referred to as U and Z, respectively.

First, PEG was selected due to its widespread application in surface shielding of nanomaterials. Its ability to hinder rapid cellular uptake by the mononuclear phagocyte system (MPS) ensures prolonged blood circulation. Specifically, we utilized methoxy PEG-azide (mPEG-N_3_, *M*_W_ = 5 kDa), following optimization findings reported by Wang et al.^67^ Next, based on previous studies for the coating with inorganic nanoparticles,^68^ an azide-modified cell-penetrating peptide (CPP) was selected. The DynPro (dynein-propelled) is a CPP derived from the product of the E183L gene of the African swine fever virus (p54). It has been reported that DynPro facilitates cellular uptake of DynPro-derivatized nanoparticles.^68^ The primary amine moiety of DynPro′s lysine residue was utilized for fluorescent labeling with 5-carboxytetramethylrhodamine (TAMRA). Lastly, we investigated the functionalization with an azide-functionalized mannose. In this study, we specifically targeted the A549 lung epithelial cancer cell line, which has been reported to overexpress the mannose receptor.^69^

In a typical experiment, the selected ligands (Figure S31 and Table S18) reacted with the DPMA-coated NMOFs in water for 30 min at 37 °C and washed three times with water. The ratio between the ligands and the NMOFs was independently optimized for each ligand to obtain similar coating densities. Therefore, eight samples were produced: Z, Z@PEG, Z@Man, Z@CPP, U, U@PEG, U@Man, and U@CPP. Following functionalization and redispersion in water, the *d*_h_ measured using DLS and NTA in all cases remained consistent with the original NMOFs (see Figures 4a,b, and S32, Tables S19–S20). Changes in ζ-potential values indicated successful particle functionalization, with a notable reduction observed in the absolute value of the initial surface charge. This reduction can be attributed to the chosen molecules being either neutral (e.g., PEG and mannose) or positively charged (e.g., CPP) (see Figure 4c,d).

**Figure 4:**
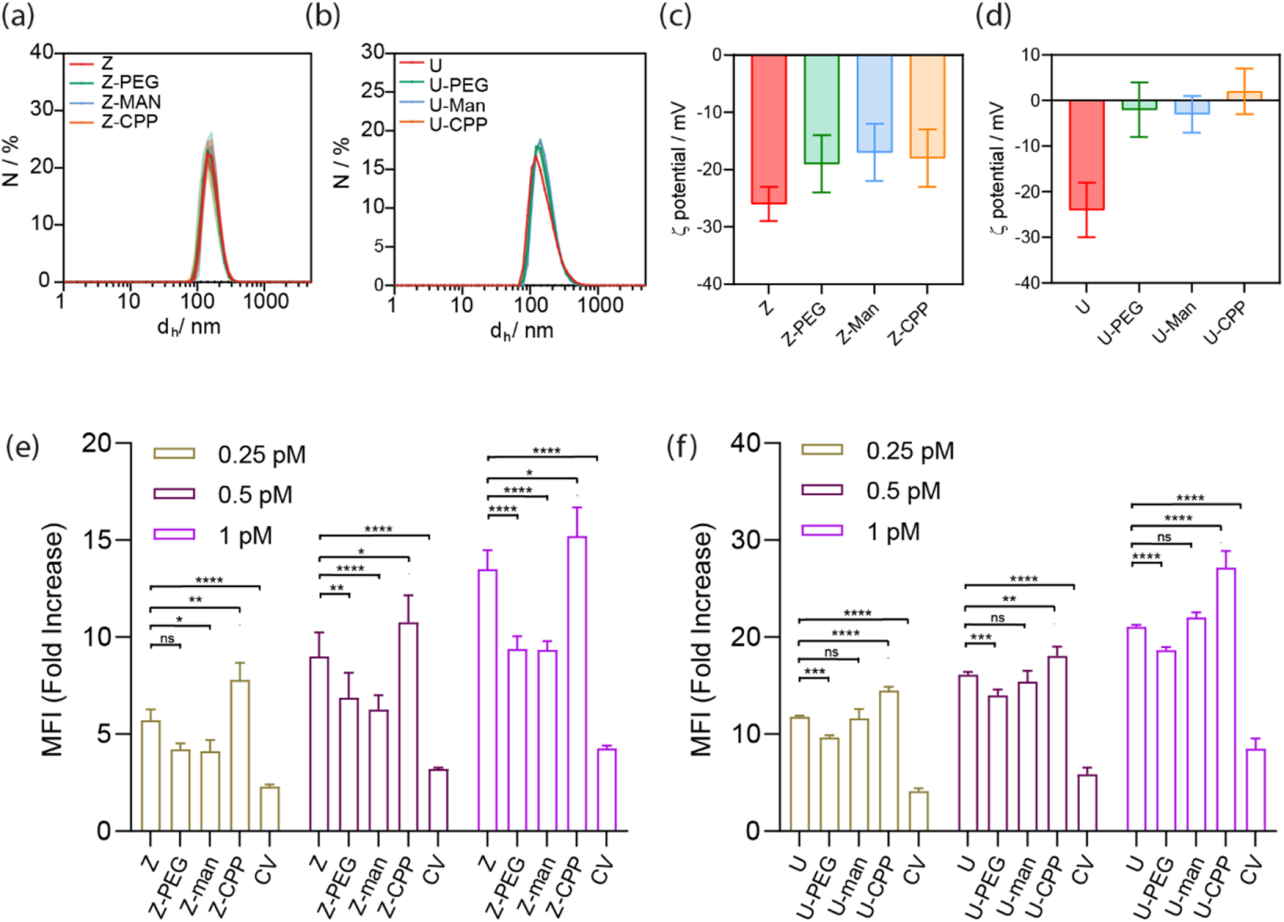
Sample characterization after the click-chemistry-based functionalization using DLS (a) Z and (b) U, and ζ-potential measurements for (c) Z and (d) U. Flow cytometry measurements of the particle uptake in A549 cell line for (e) Z and (f) U samples where Z = ZIF-8­(CV)@PMA, U = UiO-67­(CV)@PMA. Mean fluorescence intensity was recorded using a 532 nm green laser coupled to a 620/50 filter. Experiments were carried out using *n* = 3. Error bars represent standard deviation. Statistical analysis was assessed by two-way ANOVA test (***p = 0.0018, ****p < 0.0001, ns = no significant difference).

Subsequently, we assessed the interaction between the NMOFs and A549 cells. After confirming the low toxicity of our NMOFs (see above), we investigated the cellular uptake of the functionalized samples using flow cytometry (Figure 4e,f) and fluorescence microscopy (Figure 5a,b, see Figure S33 for controls of untreated cells and free CV) by tracking the CV emission. Quantitative uptake analysis was performed using doses of equivalent CV-loaded NMOFs using flow cytometry. As depicted in Figure 4e, the cell internalization of Z-CPP notably surpasses the other samples. These results align with our expectations; CPPs are known to enhance cell recognition, thereby promoting increased cellular internalization of CPP-functionalized particles.^33,44^ Conversely, Z-PEG samples exhibit reduced uptake compared to nonfunctionalized particles, highlighting the shielding potential of PEG polymer in diminishing cell recognition. Contrary to our expectations, which are based on the overexpression of mannose receptor (MR) in A549 cells,^29^ the reduced uptake of Z-man may be attributed to several factors: (i) the sparse density of mannose on the NMOF surface, (ii) the mannose modification with the azide group in the anomeric group which could hinder its recognition abilities, and (iii) the particle increased hydrophilicity by the glycan modification, which may reduce particle interactions with hydrophobic membranes.^24,31^ Moreover, this modification appears to inhibit nonspecific protein adsorption (protein corona) similarly to the effect observed with PEGylated samples.

**Figure 5:**
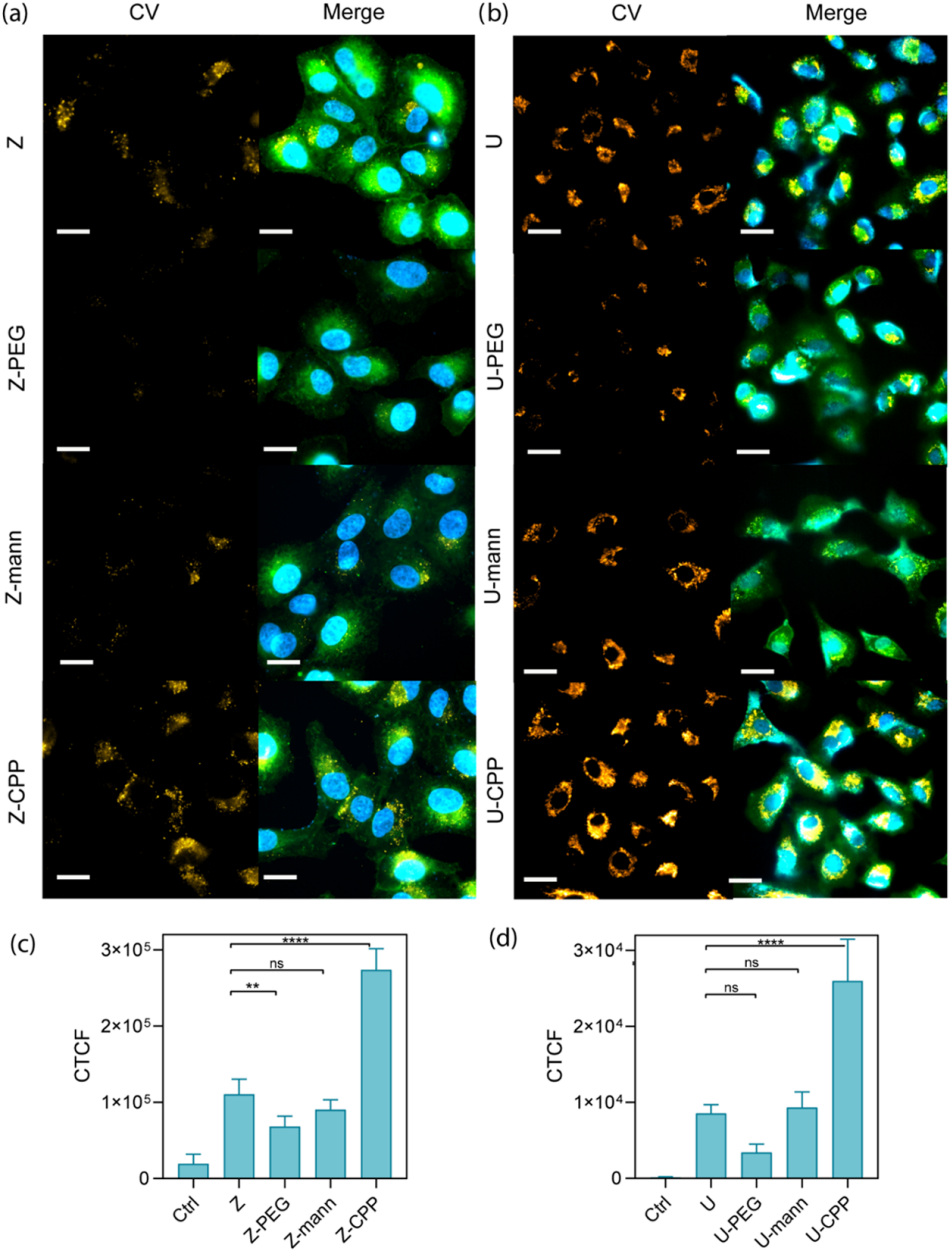
Fluorescence microscopy images of A549 cells incubated with NMOFs (a) ZIF-8 and (b) UiO-67 with the different surface functionalizations (PEG, mannose and CPP) for 3 h at a concentration of 0.5 pM. Left column (orange) corresponds to CV fluorescence and right column to merged channels: DAPI (ex. 391/32, em. 473/22), cell mask (em. 478/33, ex. 519/25), and CV (ex. 578/24, em. 641/78). Corrected total cell fluorescence (CTCF) values for each sample of (c) ZIF-8 and (d) UiO-67. Scale bars: 20 μm.

At this point, we are testing two libraries of functionalized NMOFs: UiO-67­(CV)@DPMA (U) and ZIF-8­(CV)@DPMA (Z). Just to summarize, UiO presents an octahedral shape while ZIF-8 are cubes. Nevertheless, in both cases, the overall hydrodynamic size is approximately 140 nm for the uncoated NMOFs. DLS measurements also confirm that their hydrodynamic sizes were similar once coated with the amphiphilic polymer. Once the NMOFs are polymer-coated, the exposed surface will be the polymer surface, making both systems virtually equivalent considering the hydrodynamic measurements. For all this, we consider that the differences in uptake that we observed are a consequence of the biofunctionalization with PEG, CPP, or mannose.

To verify the cargo retention within the NMOFs, as control, we examined the uptake of free CV using equivalent quantities (∼100 nM) based on fluorescence measurements. The uptake of free CV is substantially reduced compared to the encapsulated CV, indicating both the efficient internalization of the NMOFs and the successful retention of CV. The uptake trend observed in U closely resembles the one of Z library (see Figure 4f). Specifically, U-CPP exhibited an increased uptake, while U-PEG showed a decreased uptake compared to the control. However, the uptake of U-Man was similar to the uncoated sample U.

Fluorescence microscopy imaging experiments were conducted to further study the uptake and the intracellular localization of the NMOFs. Note that to elucidate the uptake mechanisms, a more systematic study using relevant uptake inhibitors should be conducted. NMOFs were incubated for 3 h at a concentration of 0.5 pM. The images clearly depicted the intracellular localization of all NMOFs­(CV) in the perinuclear region, consistent with our expectations (Figures 5 and S34).^1,49^ The microscopy results are in agreement with the flow cytometry findings in both Z (Figure 5a) and U samples (Figure 5b). CPP significantly enhances cell recognition of both NMOFs, resulting in increased uptake, whereas PEG shields the particles, thereby reducing internalization. Again, mannose-labeled NMOFs do not show the expected improvement in uptake, and quantitative analysis of corrected total cell fluorescence per cell (CTCF, Figure 5) shows no significant differences compared with controls. The internalization of ZIF-8 samples was further corroborated by acquired images using a Leica Thunder microscope (see Figure S34). These images allow us to confirm that the NMOFs are in the same focal plane as the nucleus, providing strong evidence for the internalization of the NMOFs rather than surface adhesion after the incubation times. The NMOFs appear as distinct punctate signals in the perinuclear region with no notable differences between the different coatings. This distribution typically correlates with their localization in lysosomes, which aligns with expectations, as NMOFs within the 100–200 nm size range used to be internalized via endocytosis. This intracellular distribution was constant independently of the surface modification of the NMOFs. To further confirm this observation, colocalization studies were conducted using stimulated emission depletion microscopy (STED) in A549 cells exposed to the modified UiO-67­(CV)@DMPA (U-PEG, U-Mann and U-CPP) using human transferrin (HT) to stain lysosomes (Figures S35 and S36, and Table S21). Quantitative analysis was carried out and it was observed that there was a significant colocalization of the CV signal from the NMOFs and the HT. This moderate colocalization is supported by the values of Pearson′s coefficient (in the range of 0.54–0.59 (Table S21)), and Manders′ coefficients M1 (0.31–0.37) and M2 (0.55–0.58). There were no significant differences among the different coatings.

When comparing the flow cytometry and microscopy data, both methodologies showed consistent and aligned results regarding the cellular uptake of functionalized NMOFs. Notably, NMOFs modified with CPP displayed significantly higher internalization rates in A549 cells compared to those coated with mannose or PEG. These findings highlight the effectiveness of the proposed functionalization strategy for binding biomolecules, thereby modulating NMOF–cell interactions. This is a crucial point in developing drug delivery systems and enhancing targeted therapies in biomedicine.

## 3 Conclusions

In summary, our study introduced an innovative approach for the surface modification of nanosized metal–organic frameworks (NMOFs) using a versatile, clickable amphiphilic polymer derivatized with dibenzo cyclooctyne groups. A selection of NMOFs, including UiO-67, NU-1000, PCN-222, and ZIF-8, were synthesized with high monodispersity and uniformity, and successfully coated with the amphiphilic polymer PMA. This coating significantly enhanced the structural and colloidal stability of the NMOFs in various biologically relevant media, addressing a major challenge of the proposed Zr and Zn NMOFs and expanding their potential in different bioapplications.

Additionally, the selected NMOFs were loaded with cresyl violet (CV), a common histological stain and lysosomal marker, demonstrating exceptional loading capacities and sustained cargo retention post-PMA functionalization. For comparative purposes, two size-equivalent (150 nm) NMOFs, ZIF-8 and UiO-67, were selected and functionalized with a library of biologically relevant azide-derivatized (macro)­molecules, including poly­(ethylene glycol), mannose, and a cell-penetrating peptide using a biorthogonal reaction.

The derivatized NMOFs demonstrated distinct cellular uptake patterns, which are attributed to their specific surface modifications. This underscores the effectiveness and adaptability of the proposed coating strategy. These results highlight the potential of the newly introduced universal, biorthogonal surface engineering to modulate NMOFs-cell interactions, expanding their applicability in diverse biomedical applications, including theranostic and controlled drug delivery.

## 4 Experimental Section

### 4.1 Materials and Reagents

Zinc nitrate hexahydrate (Zn­(NO_3_)_2_·6H_2_O; Sigma-Aldrich #96482), 2-methylimidazole (MeIm; Sigma-Aldrich #M50850), zirconyl chloride octahydrate (ZrOCl_2_·8H_2_O 98%, Sigma-Aldrich, #224316); zirconium­(IV) *n*-butoxide (80 wt % in 1-butanol,Sigma-Aldrich); 4,4′-Biphenyldicarboxylic acid (C_14_H_10_O_4_, 98%, BPDC, ACROS Organics, #A0420549); tetrakis­(*p*-benzoate)­pyrene (TBAPy), tetrakis­(4-carboxyphenyl) porphyrin (TCPP), *N*,*N*-dimethylformamide (DMF, ≥99.8%, Fisher Scientific); chloroform (CHCl_3_, ≥99.8%, Fisher Scientific); acetic acid glacial (CH_3_COOH, 99.7%, Fisher Scientific), benzoic acid (≥99.5%, Sigma-Aldrich); cresyl violet acetate (CV, ACROS ORGANICS), hexadecyltrimethylammonium bromide (CTAB, ≥98%, Sigma-Aldrich), poly­(ethylene glycol) methyl ether (mPEG, *M_n_* = 5000, Sigma-Aldrich), mannose-azide (≥99%, Sigma-Aldrich, Man-N_3_), Methoxypoly­(ethylene glycol) azide (*M_n_* = 5000, mPEG- N_3_), Fmoc-protected amino-acids (>99%, Carbolution), piperidine (>99%, Alfa Aesar), diisopropyl carbodiimide (DIC, >99%, Carbolution) Oxyma (>99%, Carbolution), N-HBTU (>99%, Carbolution), N-HATU (>99%, Carbolution) diisopropyl-ethyl amine (DIEA), (≥99%, Sigma-Aldrich), 5-carboxytetramethylrhodamine (TAMRA, >99%, Carbosynth), Rink amide Protide (LL) resin (CEM), Cyanine5 azide (Cy5-N_3,_ Lumiprobe) and methanol (MeOH) were used as purchased without any purification.

### 4.2 Synthesis of ZIF-8 NMOFs

In a 5 mL glass vial, under magnetic stirring at room temperature (RT), 1 mL of a 1.3 M aqueous solution of 2-methylimidazole, 1 mL of a 0.025 M aqueous solution of zinc nitrate hexahydrate, and 1 mL of a 1.6 × 10^–3^ M CTAB aqueous solution were mixed. The mixture was stirred for 5 min and left to stand for 3 h at room temperature. The development of whitish turbidity signified the formation of ZIF-8. Subsequently, the particles were washed by centrifugation at 7200 rcf for 10 min and were rinsed twice with methanol (MeOH).

### 4.3 Synthesis of UiO-67 NMOFs

51 mg of ZrOCl_2_·8H_2_O and 72.5 mg of BPDC were weighed in two separated 50 mL falcons and redispersed in 25 mL of DMF each. Then, a heating plate and the adaptor for the 50 mL round flask were prepared at 120 °C. When the temperature was reached, 5 mL of ZrOCl_2_·8H_2_O and 5 mL of BPDC were added into a 50 mL round flask and the mixture was sonicated for 30 s. Then, 0.4 mL of AA was added to the solution. After sonication for another 30 s, the round flask was placed in the heating plate and left at 120 °C overnight. Subsequently, the particles were washed by centrifugation at 7200 rcf for 10 min and were rinsed twice with DMF and once in acetone.

### 4.4 Synthesis of NU-1000 NMOFs

In a 20 mL glass vial, a mixture was prepared by dissolving 64 mg of ZrOCl_2_·8H_2_O and 10 mg of H_4_TBAPy in 10 mL of DMF. Subsequently, 2 mL of AA and 1 mL of water were added. The vial was sealed and heated at 90 °C for 30 min. Following this, the resulting product was centrifuged at 7200 rcf for 10 min, with three subsequent washes using DMF and three washes with acetone. The product was then dried under vacuum for its subsequent characterization.

### 4.5 Zr Cluster

100 mL of 1-propanol and 5 mL of Zr (IV) n-butoxide (80 wt %) were combined and stirred for 10 min. Subsequently, 33 g of benzoic acid were added, and the mixture was ultrasonicated for 20 additional minutes. The solution was then heated at reflux and stirred overnight. The following day, it was dried using a rotary evaporator, and the resulting solid product was washed three times with 1-propanol and centrifuged at 7000 rcf for 10 min each time. The final product was dried under vacuum at room temperature. Any nonvolatile residues of 1-propanol were washed and removed with MeOH, and the solid was dried under vacuum at room temperature. This cluster was utilized for the synthesis of PCN-222.

### 4.6 Synthesis of PCN-222 NMOFs

22.5 mg of TCPP (28.5 μmol) were weighed directly in a 20 mL glass vial and 8 mL of DMF were added using a 10 mL syringe and sonicated. Then, 38 mg of the Zr_6_-oxo cluster and 230 μL of TFA were added to the solution under magnetic stirring. The reaction was sealed and left in a block heater at 120 °C under magnetic stirring for 5h. the resulting product was centrifuged at 7200 rcf for 35 min, with three subsequent washes using DMF and three washes in ethanol. The final product was stored in 1 mL of ethanol.

### 4.7 CV Encapsulation

UiO-67 NPs dispersed in acetone (200 μL, 1 mg·mL^–1^) were mixed with a solution of CV in MeOH (200 μL, 2 mg·mL^–1^) in a weight relation of 1:2 (NMOF/CV). The mixture was incubated for 3 days under stirring at 60 °C to ensure that the maximum loading was reached regardless of the diffusion kinetics of the dye through the UiO-67 pores. Particles were collected by centrifugation (10,000 rcf, 10 min) and washed three times in acetone (10,000 rcf, 10 min). The process was analogous for NU-1000 and PCN-222, but for these NMOFs the loading was performed at room temperature. For further PMA functionalization, particles were redispersed in acetone.

### 4.8 Synthesis of ZIF-8­(CV) NMOFs

In a 5 mL glass vial, under magnetic stirring at room temperature (RT), a mixture was created by combining 1 mL of a 1.3 M aqueous solution of 2-methylimidazole, 1 mL of a 0.025 M aqueous solution of zinc nitrate hexahydrate, and 1 mL of a 1.6 × 10^–3^ M CTAB aqueous solution containing CV in a relation of 1:4.75. The mixture was stirred for 5 min and left to stand for 3 h at room temperature. The development of violet turbidity signified the formation of ZIF-8­(CV). Subsequently, the particles were washed by centrifugation at 7200 rcf for 10 min and were rinsed twice and redispersed in 1 mL of MeOH.

### 4.9 PMA Synthesis

PMA-based amphiphilic polymer (i.e., poly [isobutylene– alt–maleic anhydride]–graft–dodecyl) was synthesized following a protocol previously described by Parak et al. In a 50 mL round flask, 20 mmol of poly­(isobutylene-*alt*-maleic anhydride) and 15 mmol of dodecylamine (DDA) were mixed in 40 mL of tetrahydrofuran (THF). The solution was kept under magnetic stirring (350 rpm) and reflux (65 °C) overnight. Then, the THF was evaporated under vacuum using a rotary evaporator and the dried polymer was redispersed in 10 mL of chloroform. To purify the polymer and remove the excess of unreacted DDA, 50 mL of hexane were added to the solution and left for 2 h in the freezer (−20 °C). The sample was centrifuged (2 min, 7100 rcf) to remove the hexane and dried under vacuum. The final powder was weighed to obtain a final modified monomer (*M*_W_ = 209 g·mol^–1^) concentration of 0.5 M in MeOH.

### 4.10 PMA Modification with DBCO (DPMA)

For the reaction, 20 mmol of poly­(isobutylene-*alt*-maleic anhydride), 15 mmol of dodecylamine (DDA) and 0.4 mmol of DBCO were mixed in 40 mL of tetrahydrofuran (THF). The solution was kept under magnetic stirring (350 rpm) and reflux (65 °C) overnight. Then, the THF was evaporated under vacuum using a rotary evaporator and the dried polymer was redispersed in 10 mL of chloroform. To purify the polymer and remove the excess of unreacted DDA, 50 mL of hexane were added to the solution and left for 2 h in the freezer (−20 °C). The sample was centrifuged (2 min, 7100 rcf) to remove the organic solvents and dried under vacuum. The powder was weighed to prepare a final solution in chloroform of a modified monomer (*M*_W_ = 215 g·mol^–1^) concentration 0.5 M.

### 4.11 PMA Coating of Zr NMOFs

All Zr MOFs were mixed with a chloroform solution of PMA (300 monomers per nm^2^ surface area of NP, considering for the calculations a sphere with the same NC diameter) and the solvent (3:1 acetone:CHCl_3_) was slowly evaporated in a rotary evaporator. Then the dried product was resuspended by adding approximately 0.5 mL of sodium hydroxide (0.001 M, pH 11), aided by sonication (3 min). The resulting nanoparticles (NMOFs@PMA) were collected by centrifugation at 7200 rcf for 10 min, washed twice with sodium hydroxide (0.001 M, pH 11), and finally redispersed in Milli-Q water.

### 4.12 PMA Coating of ZIF-8 and ZIF-8­(CV)

NMOFs dispersed in MeOH were mixed with a chloroform solution of PMA (600 monomers per nm^2^ surface area of NP, assuming a spherical particle) and the solvent (3:1 MeOH/CHCl_3_) was slowly evaporated in a rotary evaporator. Then the dried product was resuspended by adding approximately 0.5 mL of sodium hydroxide (0.1 M, pH 13), aided by sonication (1 min). The resulting nanoparticles (ZIF-8/ZIF-8­(CV)@PMA) were collected by centrifugation at 7200 rcf for 10 min, washed twice with sodium hydroxide (0.01 M, pH 12), and finally redispersed in Milli-Q water.

### 4.13 Synthesis of CPP-N_3_

DynPro peptide sequence (GGGGKHPAEPGSTVTTQNTASQTMSRRRRRRRR) was carried out by robotic solid phase peptide synthesis on a CEM Liberty Lite 1.0 apparatus. Rink amide resin (0.025 mmol) was selected as solid support for the synthesis. For iterative coupling cycles, 20% piperidine in DMF was utilized for Fmoc-cleavage phase, whereas DIC/Oxyma were used as carboxy-activating mix. Standard microwave-assisted coupling protocols following peptide synthesizer’s manufacturer guidelines were employed. After peptide elongation and cleavage of the N-terminus Fmoc group, a manual coupling of N_3_–PEG_4_ moiety was carried out by the addition of a premixed solution of N_3_–PEG_4_-COOH (2 equiv), N-HBTU (2 equiv) and DIEA (8 equiv) in DMF (2 mL) and stirring of the resulting mixture under nitrogen stream for 30 min followed by washings with DMF (3 × 3 mL) and DCM-HPLC (3 × 3 mL). For the introduction of TAMRA fluorophore, Lys-Mtt protecting group was cleaved on-resin by using mild acidic conditions consisting DCM/HFIP/TFE/TIS (6.5/2/1/0.5, 2 × 2 mL, 2h) followed by the addition of a premixed solution of TAMRA (1 equiv), N-HATU (1 equiv) and DIEA (2 equiv) in DMF (2 mL) and the mixture was stirred under nitrogen stream for 30 min followed by washings with DMF (3 × 3 mL) and DCM-HPLC (3 × 3 mL). Finally, the peptide was deprotected and cleaved from the resin by standard TFA cleavage procedure at rt by using TFA/DCM/H_2_O/TIS (90:5:2.5:2.5, 1 mL per 70 mg of resin) for 2 h. Then, the mixture was filtered, washed with TFA (1 mL) and the peptide was precipitated with ice-cold Et_2_O (25 mL). The precipitate was centrifuged, dissolved in H_2_O (5 mL) and purified by semipreparative reverse phase chromatography in an JASCO HPLC with an Agilent Eclipse XDB-C18 column (9.4 mm × 250 mm). A binary mixture of solvent A: H_2_O with 0.1% TFA; solvent B: CH_3_CN with 0.1% TFA, with a gradient of 95:5 (5 min), 95:5 → 5:95 (5 → 35 min); 2.5 mL/min was employed. Pure fractions of the target peptide were collected and freeze-dried to provide the product as a pink powder (5.4 mg. 5% yield). Peptide identity was confirmed by analytical HPLC-MS (Agilent SB-C18 column; 95:5 → 5:95 (0 → 12 min); 0,5 mL/min, Figure S37). *R*_t_: 6.2 min; *m*/*z* (%) 634 (21, [M + 7H + TFA]^7+^), 740 (48, [M + 6H + TFA]^6+^]), 760 (42, [M + 6H + 2TFA]^6+^), 866 (36, [M + 5H]^5+^)­888 (25, [M + 5H + TFA]^5+^), 912 (32, [M + 5H + 2TFA]^5+^), 1083 (29, [M + 4H]^4+^) 1109 (100, [M + 4H + TFA]^4+^), 1138 (77, [M + 4H + 2TFA]^4+^), 1516 (15, [M + 3H + 2TFA]^3+^), 1557 (25, [M + 3H + 3TFA]^3+^).

### 4.14 Postfunctionalization via Click Chemistry

NMOFs previously coated with PMA were reacted with the selected molecules (PEG-N_3_, mannose-N_3_, CPP-N_3_). Typically, 100 μL of PMA-DBCO modified NMOFs as redispersed in water were mixed with the selected azide-functionalized molecules (see Table S18) at 37 °C for 30 min. Samples were collected by centrifugation at 7200 rcf for 10 min, washed twice with Milli-Q water and redispersed in 100 μL of Milli-Q water.

### 4.15 Quantification by Fluorescence Measurements

Fluorescence characterization in solution was performed using a Horiba FluoroMax-3 spectrometer. The amount of CV molecules loaded into NMOFs was quantified by fluorescence (λ_exc_/λ_em_ = 590/620 nm) indirectly, by measuring the CV remaining in the supernatants after centrifugation and washing steps of the NMOFs. The CV concentration in the supernatant was determined by interpolation of the measured fluorescence intensity (FI) to freshly prepared calibration curves for each media.

### 4.16 Scanning Electron Microscopy

SEM micrographs were obtained using a ZEISS FE-SEM ULTRA Plus after the deposition of a drop of diluted sample onto a piece of clean silicon wafer.

### 4.17 Thermogravimetric Analysis

TGA measurements were carried out using a TA Instruments Inc. SDT Q-600 thermobalance with a general heating profile from 25 to 800 °C and a heating rate of 5 °C min^–1^ under air using a flux of 100 mL·min^–1^. Before the analysis, all the samples were lyophilized and dried at 100 °C.

### 4.18 UV–Vis Spectroscopy

UV–visible absorption spectra were recorded using an Agilent UV–vis Cary 3500 spectrophotometer. The measurements were performed using a 1 cm quartz cell at a controlled temperature of 25 °C to minimize variability. Data acquisition was carried out over a wavelength range of 200 to 1000 nm to capture the entire extinction profile.

### 4.19 Nanoparticle Tracking Analysis

A NanoSight NS300 (Malvern Instruments, U.K.) equipped with a 488 nm laser module, a sCMOS camera and a syringe pump was used for all NTA measurements. All measurements were carried out at 24 °C. NMOFs were diluted in filtered Milli-Q water to a final volume of 1 mL and loaded in the measurement chamber with a flow rate of 100 μL·min^–1^. Flow mode measurements were obtained recording 3 videos of 60 s for each measurement. The NanoSight NS300 software was used to analyze the sample (10–100 particles/frame).

### 4.20 Dynamic Light Scattering

The *d*_h_ and polydispersity index (PDI) of the nanoparticles were determined via DLS using a Malvern Zetasizer Nano ZSP equipped with a 10 mW He–Ne laser operating at a wavelength of 633 nm and a fixed scattering angle of 173°.

### 4.21 N_2_ Adsorption–Desorption Analysis

N_2_ sorption isotherms at 77 K were measured on a Micromeritics 3Flex Adsorption Analyzer. The samples (about 20–30 mg) were activated overnight under a high vacuum at 90 °C prior to analysis. The specific surface area was extrapolated within the relative pressure (*P*/*P*_0_, where P_0_ is the saturation pressure) interval of 0.05–0.3 by applying the Brunauer, Emmett & Teller (BET) equation. The data were analyzed using the 3Flex V5.03 software (Micromeritics Instrument Corp., Norcross, GA).

### 4.22 Colloidal Stability Tests

DLS measurements at different times were carried out in different media: Milli-Q water and cell culture medium (Dulbecco’s Modified Eagle Medium with phenol red, 4.5 g L^–1^ d-glucose, l-glutamine and pyruvate (DMEM Gibco, Thermo Fisher Scientific, Massachusetts) supplemented with 10% fetal bovine serum (Gibco, Thermo Fisher, Massachusetts) and 1% penicillin/streptomycin (P/S, Gibco, Thermo Fisher Scientific, Massachusetts)) and lysosomal medium (phagolysosomal simulant fluid (PSF), pH = 5; 114 mM sodium phosphate dibasic anhydrous, 114 mM sodium chloride, 0.5 mM sodium sulfate anhydrous, 0.2 mM calcium chloride dihydrate, 6 mM glycine and 20 mM potassium hydrogen phthalate).

### 4.23 High Performance Liquid Chromatography

The potential linker release (BPDC) from the MOF structure was determined using a reverse phase HPLC Jasco LC-4000 series system, equipped with a photodiode array (PDA) detector MD-4015 and a multisampler AS-4150 controlled by ChromNav software (Jasco Inc., Japan). For the quantification, isocratic conditions were used through a Purple ODS reverse phase column (C18, 5 μm, 4.6 mm × 150 mm, Análisis Vínicos, Spain) with a flow rate of 1 mL·min^–1^. The column temperature was fixed at 25 °C and the injection volume was 30 μL. The mobile phase was based on a mixture of 95:5 phosphate-buffered solution (pH 9):MeOH. Retention time and maximum adsorption (λ) for BPDC were 7.6 min and 278 nm, respectively.

### 4.24 Cargo Loading Stability

Cargo stability tests were performed at different times (*t* = 0, 4, 8, 24, 48, 120 and 240 h) in different media: Milli-Q water, cDMEM, phosphate-buffered saline (PBS 1×, pH = 7.4) and lysosomal medium (phagolysosomal simulant fluid (PSF), pH = 5; PSF (100 mL): 114 mM sodium phosphate dibasic anhydrous, 114 mM sodium chloride, 0.5 mM sodium sulfate anhydrous, 0.2 mM calcium chloride dihydrate, 6 mM glycine and 20 mM potassium hydrogen phthalate). For the analysis, 100 μL of the samples were centrifuged and the pellets were redispersed in 2 mL of the corresponding media for the analysis. At each time point, 100 μL (×3) of each sample were centrifuged to remove the SN and measure its fluorescence using the same procedure that was used previously to calculate the dye/MOF.

### 4.25 Cell Culture

The A549 (human lung carcinoma) cell line was maintained in culture in Dulbecco’s Modified Eagle Medium with phenol red, 4.5 g L^–1^ d-glucose, l-glutamine and pyruvate (DMEM Gibco, Thermo Fisher Scientific, Massachusetts) supplemented with 10% fetal bovine serum (FBS, Gibco, Thermo Fisher, Massachusetts) and 1% penicillin/streptomycin (P/S, Gibco, Thermo Fisher Scientific, Massachusetts) (DMEM). Cells were cultured at 37 °C under a 5% CO_2_ atmosphere and kept under humid conditions. After reaching 80% confluency, the cells were washed with Dulbecco’s phosphate-buffered saline (PBS, Thermo Fisher #14190169) and passaged after incubation with 0.25% Trypsin–EDTA (Gibco, Thermo Fisher Scientific, Massachusetts).

### 4.26 MTT Test

MTT (3-[4,5-dimethylthiazol-2-yl]-2,5-diphenyltetrazolium bromide) assay was performed to study cell viability after NMOFs exposure. A549 cells were seeded in 96-well plates (7500 cells/well in 100 μL of DMEM). After 24 h, the medium was removed and 100 μL of NMOFs solution diluted in fresh DMEM was added. After 1 h, each well was rinsed once with 1× PBS and 100 μL of freshly prepared MTT solution diluted in DMEM was added. 96-well plates were left 3 h at 37 °C and 5% CO_2_, then MTT solution was removed and 50 μL of DMSO were added to each well to dissolve the formazan crystals. After 10 min at 37 °C and 5% CO_2_, absorbance was measured with a plate reader (TECAN, Infinite 200 PRO) at 540 nm. The absorbance value of each well provided by the instrument is an average of nine consecutive measures in the same well. Final absorbance value for control cells (Ac) (untreated), is an average of, at least, nine different well values. The final absorbance values for samples (As) are a mean of three independent well values. Cell viability value is calculated as follows: (As/Ac) × 100.

### 4.27 Flow Cytometry Experiments

A549 cells were seeded in 48-well plates at a density of 25,000 cells/well in 0.3 mL of DMEM. After 24 h, DMEM was replaced with NMOFs solutions diluted in fresh DMEM. Experiments were performed by exposing cells to NMOFs dispersions at 0.25, 0.5, and 1 pM for 1 h. Then, cells were rinsed once with 1× PBS and were harvested after trypsinization for 2 min with 0.070 mL 0.25% Trypsin-EDTA. 0.15 mL of PBS were added to each well to recover the cells. Fluorescence intensity was measured using a Guava Millipore flow cytometer equipped with a 532 nm green laser coupled with a 620/50 filter, according to CV emission spectrum. Results are reported as the mean of cell fluorescence intensity, provided by the measurement of 5 wells (MFI). All data are presented as the mean and standard deviation (SD). Two-way ANOVA was used to test the differences between groups. Statistical significance was set at *P* < 0.05. All statistical analysis were conducted using GraphPad Prism 8.0.1.

### 4.28 Microscopy Imaging

A549 cells were seeded on μ-Slide 8 well-ibiTreat chambers (1 cm^2^ per well, Ibidi, Germany, #80826) at a density of 25.000 cells/well in DMEM. After 24 h the medium was replaced with freshly prepared 1 pM NMOFs solutions diluted in DMEM. After 1 h, cells were rinsed once with 1× PBS in order to remove noninternalized NMOFs and left in DMEM without phenol red. 3,3′-dioctadecyloxacarbocyanine perchlorate (DiO) was used to stain membranes, while Ibidi Mounting Medium with DAPI (Ibidi, Germany, #50011) was used to stain the nuclei and to preserve fluorescence intensities.

Fluorescence images of cells were captured using a Leica Microscope type DMI8 equipped with a Leica DFC9000 camera. All the images were processed with ImageJ.

## Supplementary Material



## Notes

Additional data on the (bio)­physicochemical characterization of the materials produced in this study (PDF)
