# Supplementary material for: Clickable Polymer-Based Coatings for Modulating the Interaction of Metal–Organic Framework Nanocrystals with Living Cells
Source: ACS Appl Mater Interfaces. 2025 Apr 21;17(17):24994–5010. doi: 10.1021/acsami.5c01695 (PMC12131220; doi:10.1021/acsami.5c01695)
Supplement: Supplementary file 1 [file am5c01695_si_001.pdf]

Supporting Information

Clickable Polymer-Based Coatings for Modulating the Interaction of Metal-Organic Framework Nanocrystals with Living Cells

Manuela Cedrun-Morales [a], Martina Migliavacca [c], Manuel Ceballos [a], Marta Perez-Maseda [a], Giulia Zampini [c], María Teresa Alameda Felgueiras [c], Jon Ostolaza-Paraiso [d], Marisa Juanes [e], Irene Rincón [f], David Fairen-Jimenez [d], Javier Montenegro [e], Patricia Horcajada [f], Ester Polo [b], Beatriz Pelaz [c,], and Pablo del Pino [a,*]*

[a] Centro Singular de Investigación en Química Biolóxica e Materiais Moleculares (CiQUS), Departamento de Física de Partículas, Universidade de Santiago de Compostela, 15705 Santiago de Compostela, Spain

[b] Centro Singular de Investigación en Química Biolóxica e Materiais Moleculares (CiQUS), Departamento de Bioquímica y Biología Molecular, Universidade de Santiago de Compostela, 15705 Santiago de Compostela, Spain

[c] Centro Singular de Investigación en Química Biolóxica e Materiais Moleculares (CiQUS), Departamento de Química Inorgánica, Universidade de Santiago de Compostela, 15705 Santiago de Compostela, Spain

[d] The Adsorption and Advanced Materials Laboratory (A2ML), Department of Chemical Engineering and Biotechnology, University of Cambridge, Philippa Fawcett Drive, Cambridge CB3 0AS, UK

[e] Centro Singular de Investigación en Química Biolóxica e Materiais Moleculares (CiQUS), Departamento de Química Orgánica, Universidade de Santiago de Compostela, 15705 Santiago de Compostela, Spain

[f] Advanced Porous Materials Unit (APMU), IMDEA Energy Institute, Av. Ramón de la Sagra 3, 28935 Móstoles-Madrid (España)

[*]Email: beatriz.pelaz@usc.es; pablo.delpino@usc.es

Table S1. Table comparing the advantages or disadvantages of the most commonly used post-synthetic modification strategies for surface modification of MOFs.

Functionalization	Type of molecules used[e]	Further Chemical Modification	Enhanced Colloidal Stability	Drug-loading capacity	Enhanced Cellular Uptake	Refs
Coordinative PSM[a]	Typically, carboxylates or phosphonate macromolecules (i.e., PEG, proteins, nucleic acids, etc.)	Depending on the molecule (in many cases not possible)	Yes	Yes	Yes	ref[1-3]
Covalent PSM[b]	Ligands capable of adding specific chemical functionalities (i.e., PEG, etc.)	Depending on the molecule (in many cases not possible)	Yes	Yes	Yes	ref[4-6]
Core@shell[c]	Shell of silica or other MOF	Depending on the shell	Yes	Yes	No	ref[7-10]
Core@shell[c]	Polymers (i.e., PEG, PMA, PVP, etc.)	Depending on the shell (in many cases not possible)	Yes	Yes	Yes	ref[11-12]
Cell membrane coating[d]	Fragments of cellular membranes (biomimetic coatings, i.e., cancer cells, platelets, red blood cells, etc.), Liposomes, Exosomes	Introduction of different lipids in the composition during preparation	Yes	Yes	Yes (related to the cell line)	ref[13-15]
Core@shell[c]	DPMA	Yes (SPAAC click chemistry)	Yes	Yes	Yes	This work

[a] attachment of new molecules via coordination of free metal sites; [b] formation of covalent interactions at reactive sites; [c] coating with polymers or silica; [d] coating using fragments of cell membranes. [e] ”Further chemical modification refers” to the possibility of a subsequent chemical reaction on the incorporated surface/ligands.

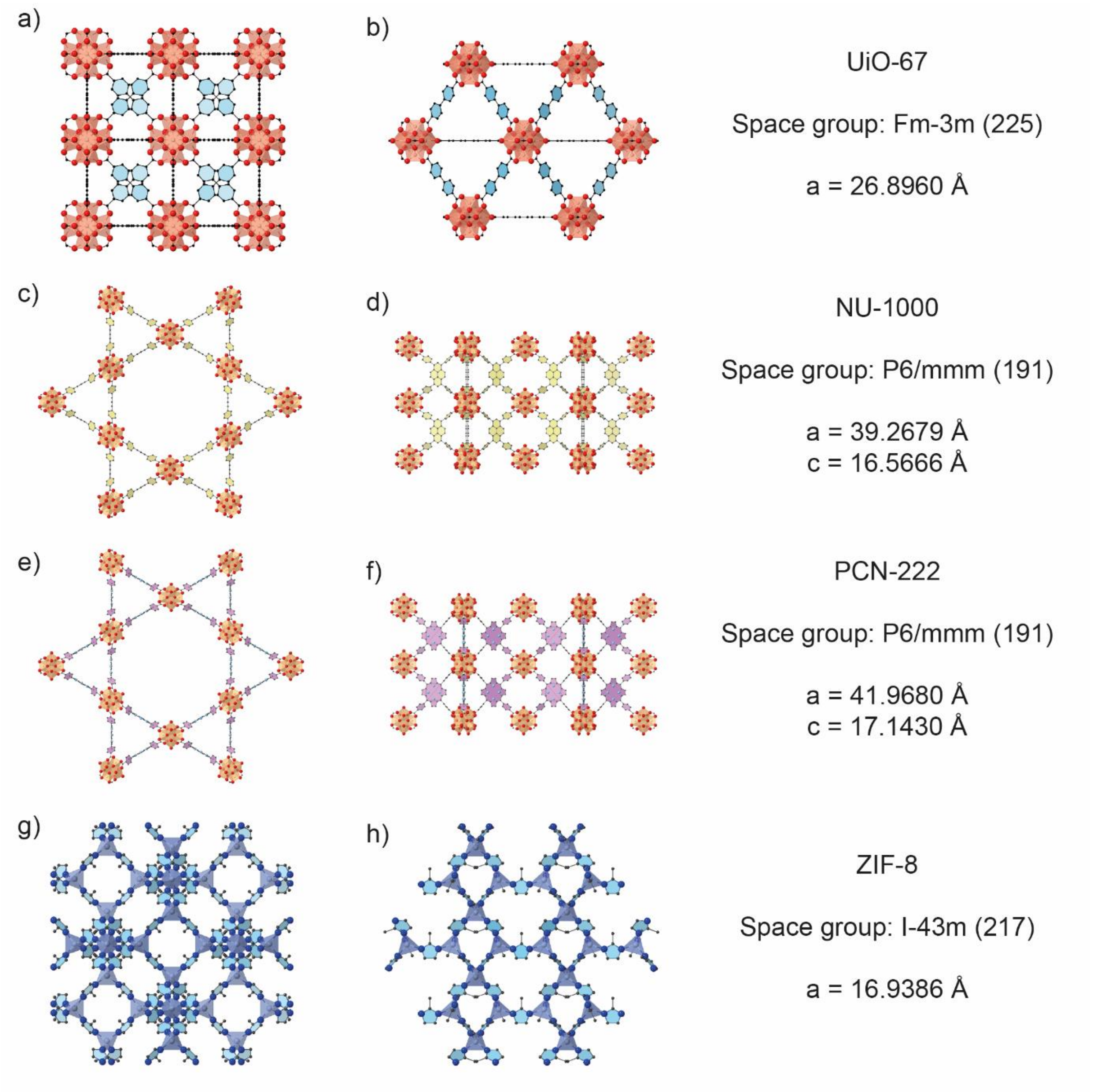

Figure S1: Crystal structure of the selected NMOFs: UiO-67 a) a-axis oriented and b) [110] oriented; NU-1000 c) c-axis oriented and d) a-axis oriented; PCN-222 e) c-axis oriented and f) a-axis oriented; ZIF-8 g) a-axis oriented and h) [110] oriented.

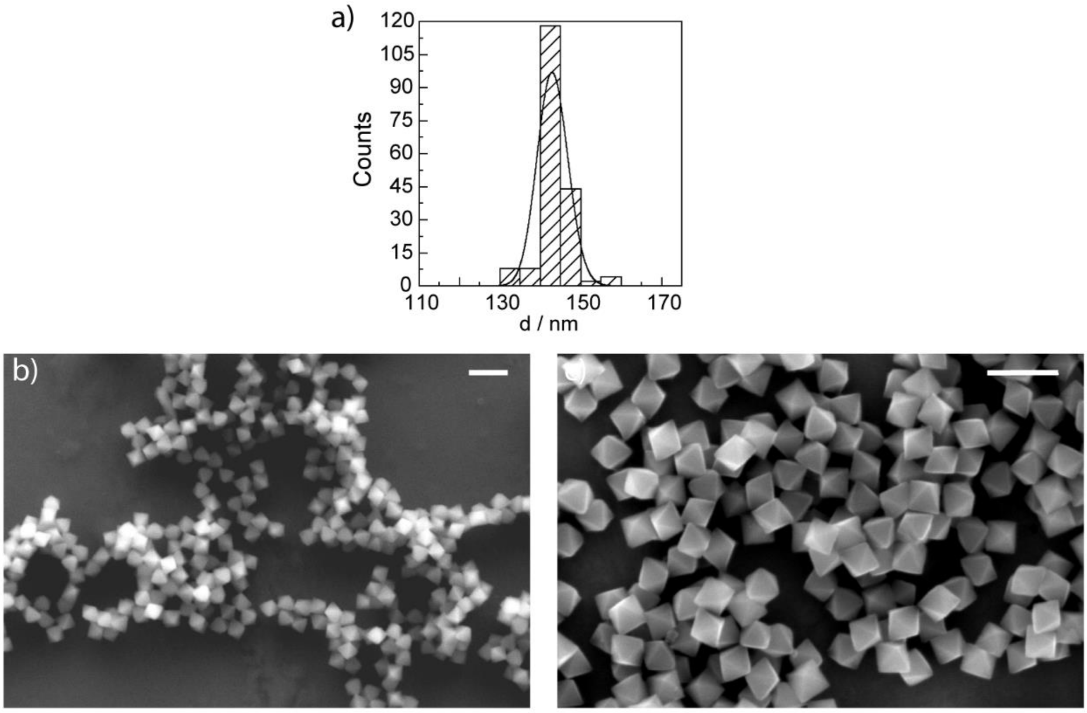

Figure S2: a) Diameter histogram based on SEM images of UiO-67, b-c) SEM images of UiO-67. Scale bars correspond to 200 nm.

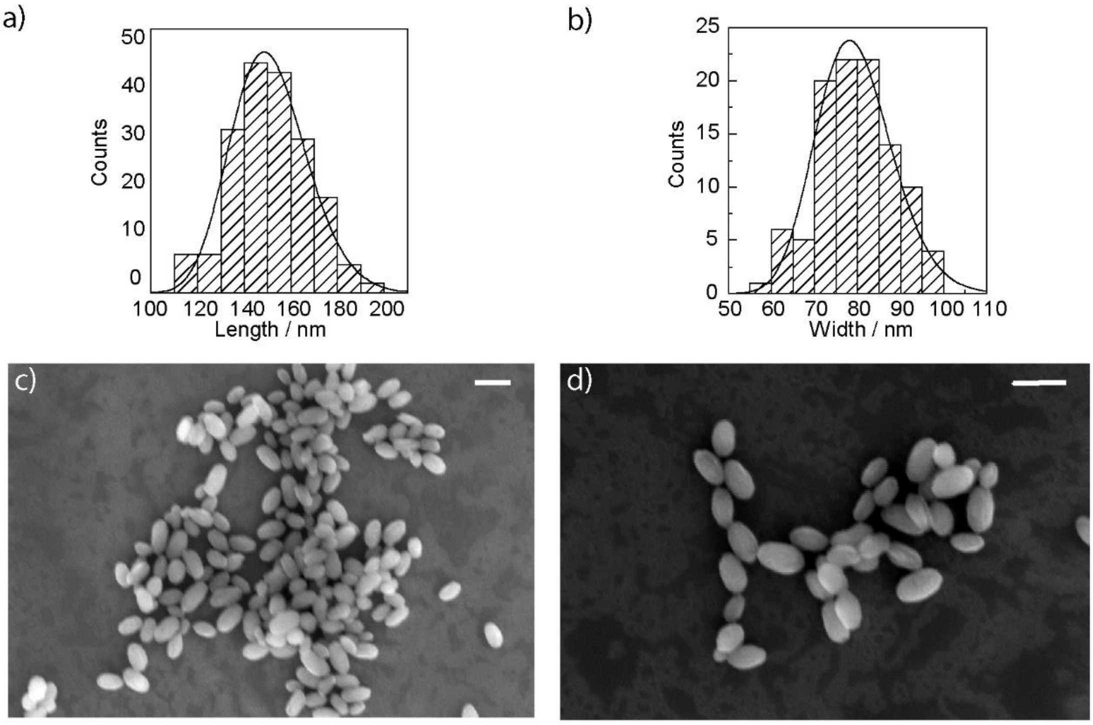

Figure S3: a) Length and b) Width histograms of NU-1000 based on SEM images, c-d) SEM images of NU-1000. Scale bars correspond to 200 nm.

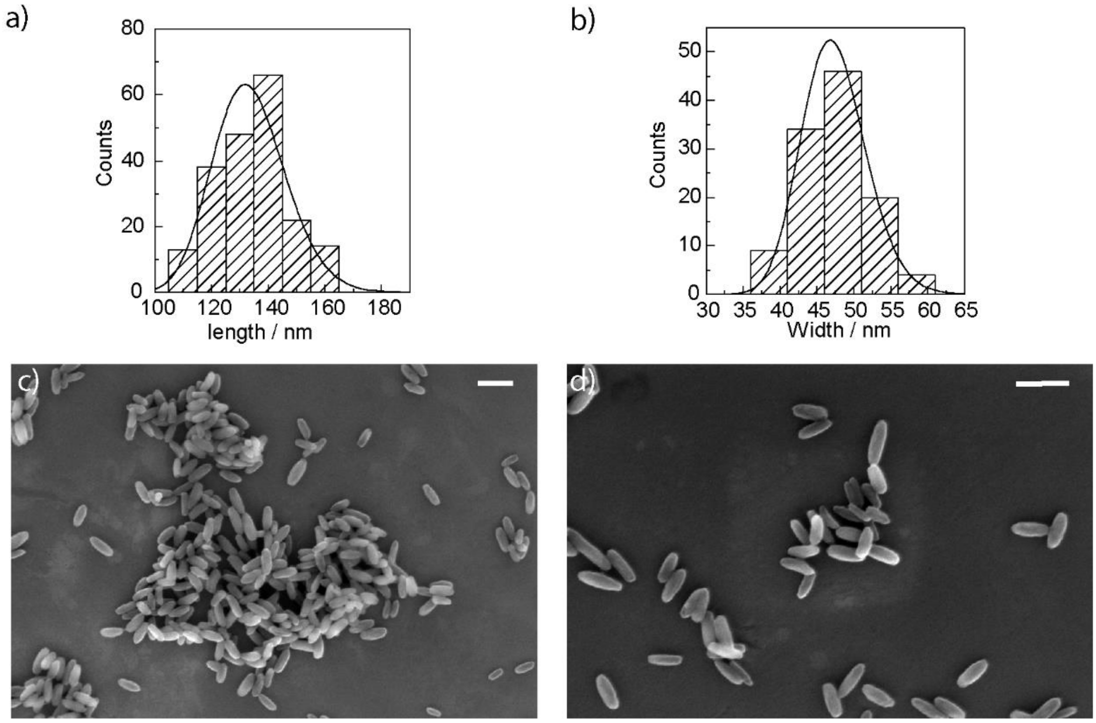

Figure S4: Size histogram based on SEM images of PCN-222, a) length and b) width; c) and d) SEM images of NU-1000. Scale bars correspond to 200 nm.

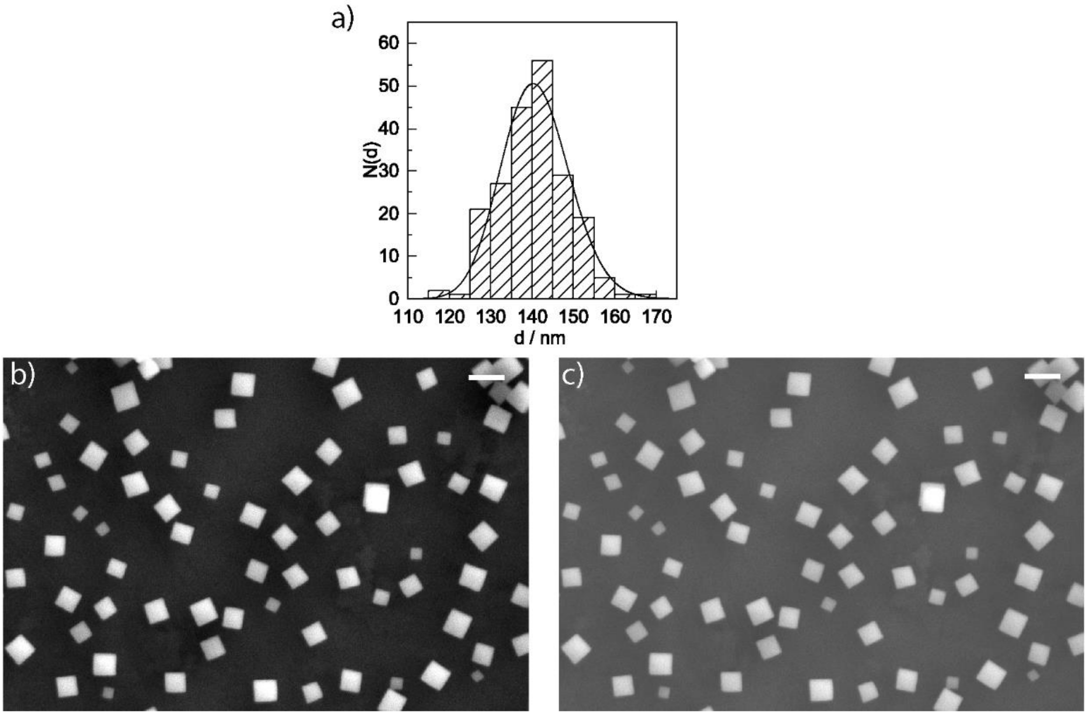

Figure S5: a) Size histogram based on SEM images of ZIF-8, b-c) SEM images of ZIF-8. Scale bars correspond to 200 nm.

Table S2. Mean size d_h (in nm) from the number and intensity distributions measured with DLS and ζ–potential results for Zr MOFs without and with PMA

Sample	N ± sd (nm)	I ± sd (nm)	V ± sd (nm)	PDI	ζ-pot (mV)
UiO-67	144 ± 11	375 ± 71	362 ± 82	0.17	15 ± 6
UiO-67@PMA	159 ± 18	275 ± 91	314 ± 59	0.14	-30 ± 7
NU-1000	150 ± 12	184 ± 8	181 ± 9	0.12	28 ± 6
NU-1000@PMA	199 ± 10	210 ± 9	211 ± 10	0.21	-22 ± 7
PCN-222	145 ± 10	280 ± 35	223 ± 33	0.20	33 ± 9
PCN-222@PMA	206 ± 8	261 ± 4	267 ± 5	0.16	-28 ± 5
ZIF-8	140 ± 6	170 ± 3	166 ± 1	0.04	36 ± 10
ZIF-8@PMA	154 ± 7	188 ± 4	187 ± 4	0.05	-30 ± 6

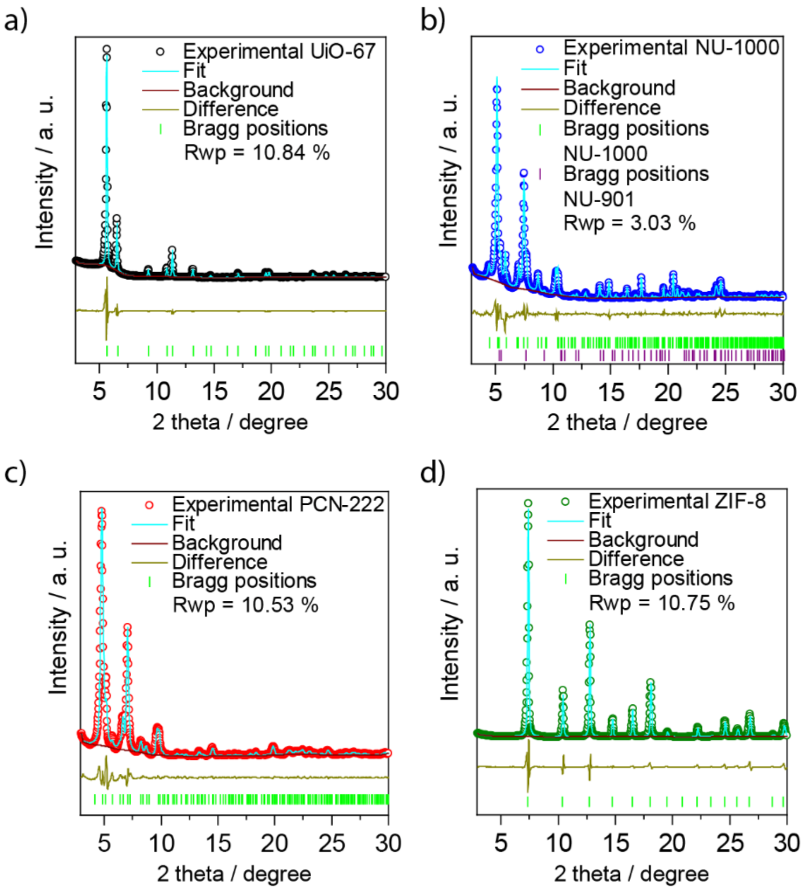

Figure S6: PXRD refinement of a) UiO-67, b) NU-1000/NU-901, c) PCN-222 and d) ZIF-8.

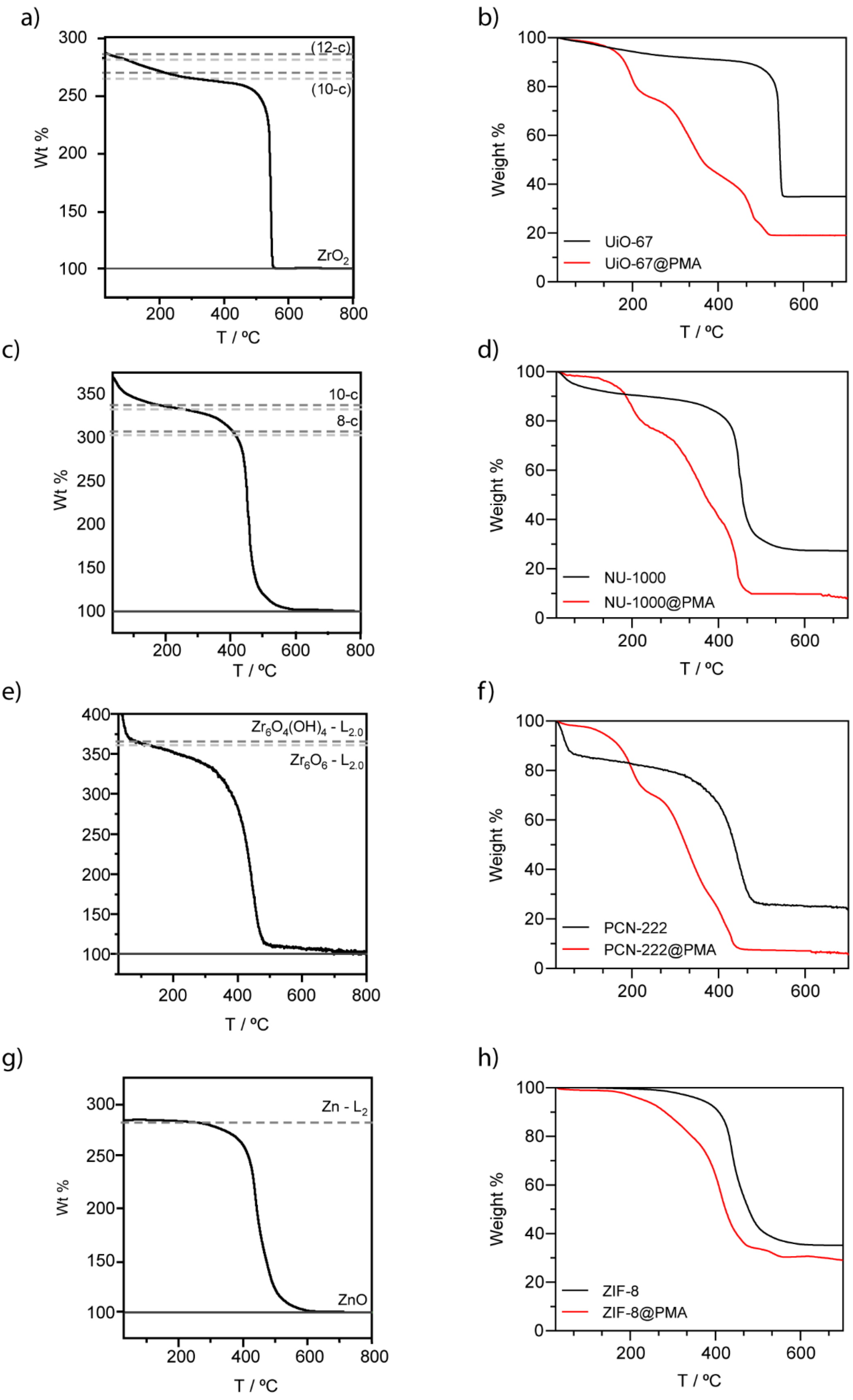

Figure S7: Normalized TGA measurements with theoretical lines of fully coordinated SBU of each MOF a) UiO-67, c) NU-1000, e) PCN-222 and g) ZIF-8; and TGA measurements before and after the PMA coating b) UiO-67,d) NU-1000, f) PCN-222 and h) ZIF-8

Table S3. Mean size d_h (in nm) from the number distribution of the hydrodynamic diameter of UiO-67 NMOFs in water, DMEM and PSF.

	Water		DMEM		PSF	
t (h)	UiO-67	UiO-67@PMA	UiO-67	UiO-67@PMA	UiO-67	UiO-67@PMA
0	144 ± 11	161 ± 18	265 ± 53	265 ± 11	178 ± 39	165 ± 5
6	255 ± 24	152 ± 30	183 ± 7	141 ± 50	562 ± 20	176 ± 5
24	358 ± 52	168 ± 13	196 ± 7	171 ± 56	651 ± 69	183 ± 12
48	359 ± 100	138 ± 35	186 ± 16	132 ± 62	406 ± 61	206 ± 12
120	128 ± 14	162 ± 7	180 ± 25	280 ± 30	489 ± 150	234 ± 49
240	195 ± 18	154 ± 8	175 ± 8	235 ± 52	532 ± 84	197 ± 56

Table S4. Mean size d_h (in nm) from the number distribution of the hydrodynamic diameter of NU-1000 NMOFs in water, DMEM and PSF.

	Water		DMEM		PSF	
t (h)	NU-1000	NU-1000@PMA	NU-1000	NU-1000@PMA	NU-1000	NU-1000@PMA
0	136 ± 6	150 ± 2	175 ± 5	215 ± 15	254 ± 12	158 ± 3
6	144 ± 14	148 ± 10	299 ± 30	204 ± 10	358 ± 51	184 ± 11
24	129 ± 7	171 ± 4	285 ± 43	210 ± 10	497 ± 51	168 ± 30
48	133 ± 6	154 ± 6	266 ± 30	224 ± 12	535 ± 90	142 ± 39
120	125 ± 7	166 ± 24	204 ± 90	218 ± 12	364 ± 145	212 ± 10
240	138 ± 10	162 ± 5	298 ± 70	211 ± 4	415 ± 103	216 ± 10

Table S5. Mean size d_h (in nm) from the number distribution of the hydrodynamic diameter of PCN-222 NMOFs in water, DMEM and PSF.

	Water		DMEM		PSF	
t (h)	PCN-222	PCN-222@PMA	PCN-222	PCN-222@PMA	PCN-222	PCN-222@PMA
0	158 ± 2	116 ± 10	254 ± 100	170 ± 10	204 ± 15	113 ± 5
6	250 ± 73	113 ± 6	355 ± 10	187 ± 12	270 ± 54	116 ± 14
24	110 ± 15	111 ± 17	510 ± 24	180 ± 5	351 ± 65	124 ± 24
48	118 ± 16	105 ± 5	593 ± 100	169 ± 23	254 ± 50	120 ± 5
120	156 ± 25	112 ± 5	365 ± 86	183 ± 13	362 ± 46	123 ± 7
240	209 ± 42	103 ± 7	405 ± 70	173 ± 6	435 ± 103	126 ± 10

Table S6. Mean size d_h (in nm) from the number distribution of the hydrodynamic diameter of ZIF-8 NMOFs in water, DMEM and PSF.

	Water		DMEM		PSF	
t(h)	ZIF-8	ZIF-8@PMA	ZIF-8	ZIF-8@PMA	ZIF-8	ZIF-8@PMA
0	140 ± 6	154 ± 7	230 ± 152	196 ± 51	265 ± 152	213 ± 51
1	155 ± 64	265 ± 36	170 ± 50	201 ± 48	153 ± 24	219 ± 39
6	72 ± 50	251 ± 62	112 ± 23	203 ± 51	80 ± 36	235 ± 23
24	-	248 ± 67	-	196 ± 48	-	211 ± 14
48	-	248 ± 23	-	203 ± 50	-	235 ± 23
120	-	247 ± 64	-	197 ± 49	-	222 ± 50

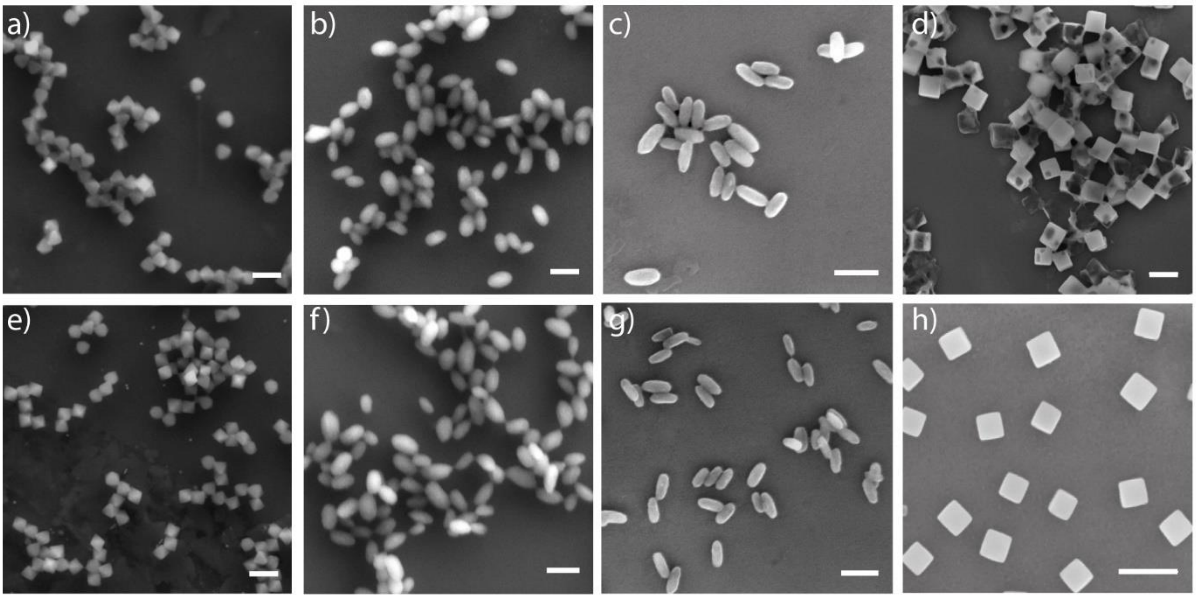

Figure S8: SEM images of the samples placed in PSF for 7 days: a) UiO-67, b) NU-1000 c) PCN-222 d) ZIF-8 (after 5h) and PMA coated samples after 7 days in PSF: e) UiO-67@PMA, f) NU-1000@PMA, g) PCN-222@PMA, h) ZIF-8@PMA. Scale bars correspond to 200 nm.

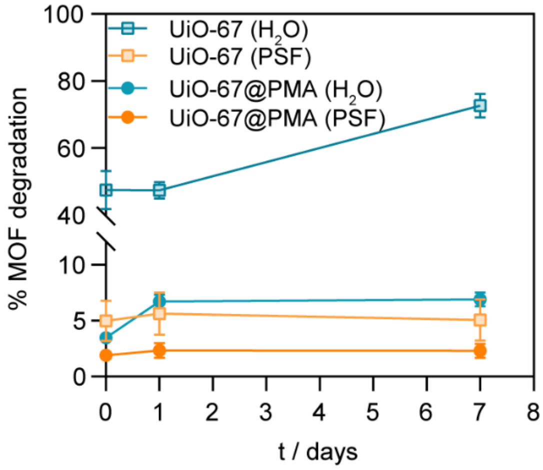

Figure S9: UiO-67 linker release in water (blue) and PSF (orange).

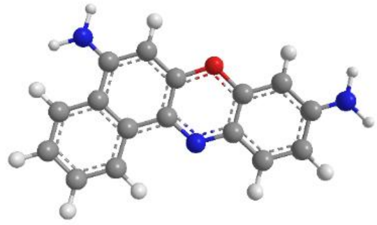

Figure S10: Cresyl violet molecule (Chem3D). C (grey), O (red) and N (blue).

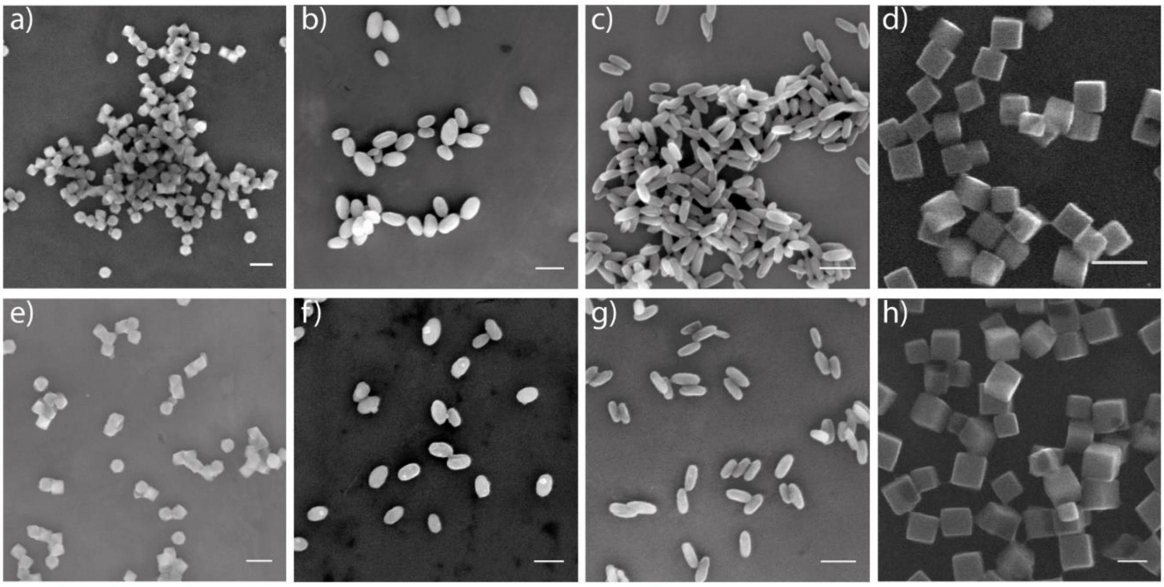

Figure S11: SEM images of the samples after the CV loading: a) UiO-67(CV), b) NU-1000(CV) c) PCN-222(CV) d) ZIF-8(CV); and a PMA coated samples after 7 days in PSF: e) UiO-67(CV)@PMA, f) NU-1000(CV)@PMA, g) PCN-222(CV)@PMA, h) ZIF-8(CV)@PMA. Scale bars correspond to 200 nm.

Table S7. Mean size d_h (in nm) from the number and intensity distributions, PDI and ζ-potentials of the samples with CV and PMA.

Sample	N ± sd (nm)	I ± sd (nm)	PDI	ζ -pot (mV)
UiO-67(CV)	196 ± 34	475 ± 71	0.17 ± 0.07	16 ± 5
UiO-67(CV)@PMA	161 ± 15	275 ± 91	0.14 ± 0.09	-24 ± 6
NU-1000(CV)	136 ± 6	158 ± 24	0.30 ± 0.03	27 ± 7
NU-1000(CV)@PMA	160 ± 12	216 ± 8	0.12 ± 0.06	-26 ± 7
PCN-222(CV)	158 ± 2	250 ± 56	0.18 ± 0.01	26 ± 6
PCN-222(CV)@PMA	132 ± 13	180 ± 8	0.11 ± 0.01	-32 ± 11
ZIF-8(CV)	138 ± 3	163 ± 2	0.04 ± 0.04	28 ± 7
ZIF-8(CV)@PMA	158 ± 6	194 ± 7	0.05 ± 0.03	-23 ± 6

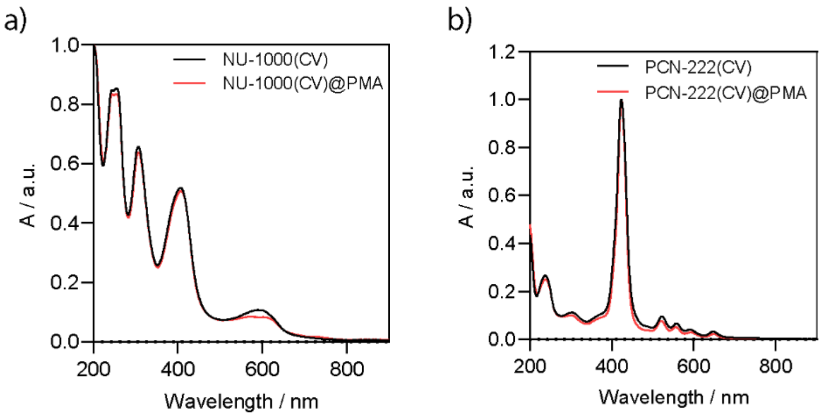

Figure S12: UV-VIS spectra of a) NU-1000(CV) and b) PCN-222(CV) before and after PMA coating.

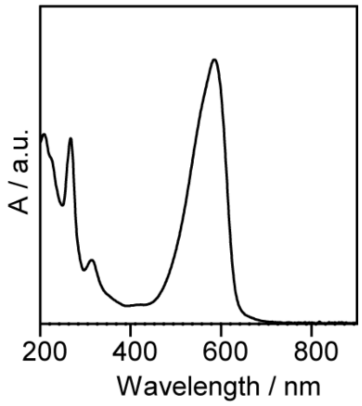

Figure S13: UV-Vis spectra of Cresyl Violet.

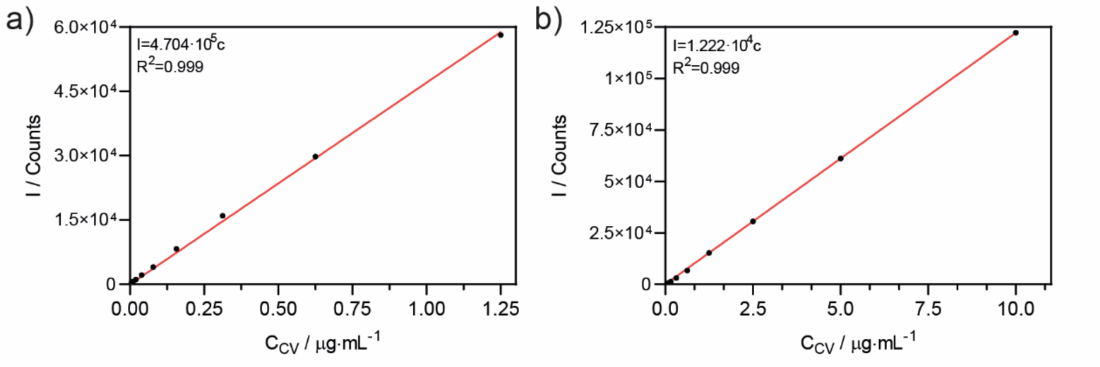

Figure S14: Calibration curve of the fluorescence of CV in a) MeOH and b) water.

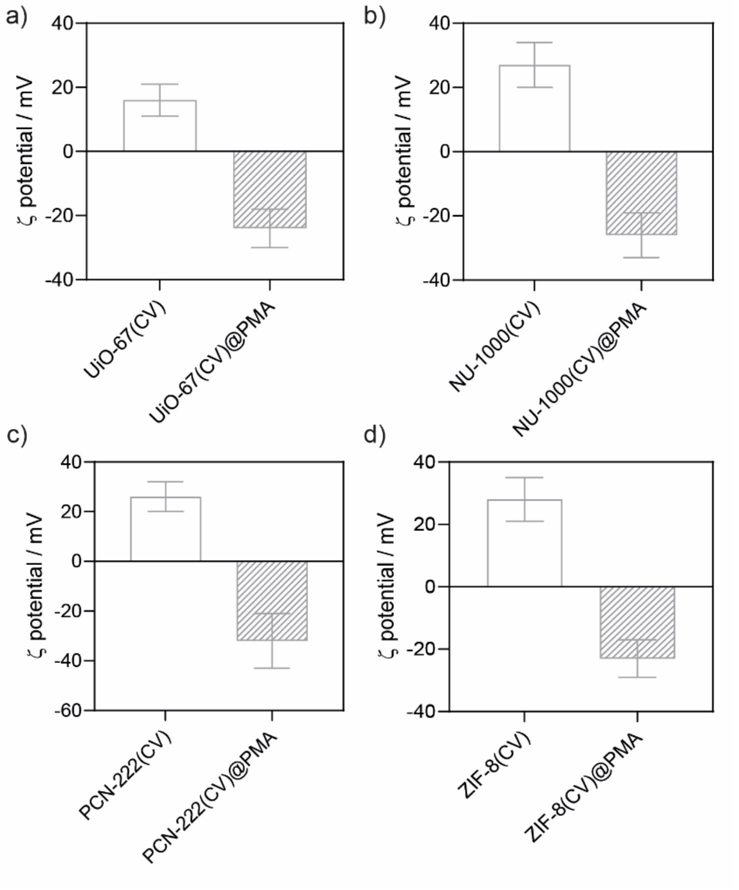

Figure S15: Zeta potential (ζ-potential) after the CV loading and the PMA coating of each MOF a) UiO-67, b) NU-1000, c) PCN-222 and d) ZIF-8.

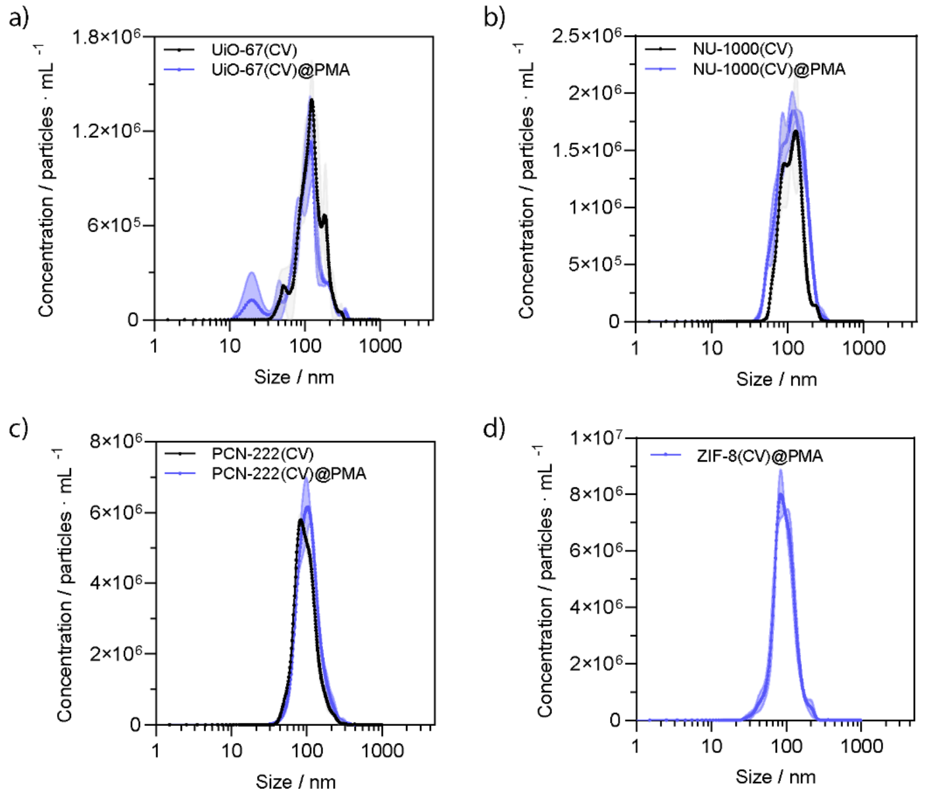

Figure S16: NTA measurements of the NMOFs diameter (nm) after the CV encapsulation and further polymer coating. a) UiO-67; b) NU-1000; c) PCN-222 and d) ZIF-8.

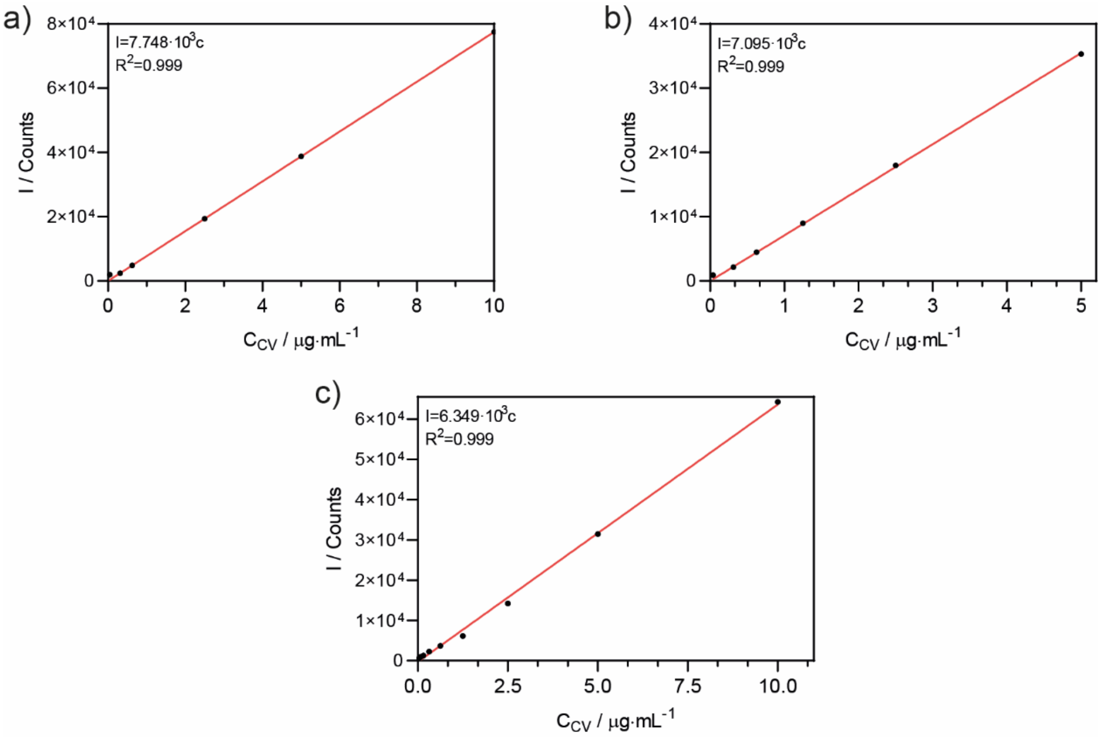

Figure S17: Calibration curves of the fluorescence of CV in a) PSF, b) DMEM and c) PBS.

Table S8. % CV released from ZIF-8(CV) in water, PSF, DMEM and PBS

ZIF-8(CV)				
t(h)	water	PSF	DMEM	PBS
0	0.00 ± 0.03	0.00 ± 0.06	0.00 ± 0.05	0.00 ± 0.06
4	0.30 ± 0.05	3.39 ± 0.31	6.37 ± 0.35	0.27 ± 0.54
8	0.36 ± 0.01	3.77 ± 2.00	7.04 ± 1.99	0.07 ± 0.02
24	0.62 ± 0.33	3.44 ± 1.60	8.42 ± 2.17	0.06 ± 0.17
48	0.73 ± 0.17	4.40 ± 0.25	15.30 ± 5.53	1.34 ± 0.06
120	1.28 ± 0.01	5.23 ± 2.15	14.72 ± 3.15	2.34 ± 1.69

Table S9. % CV released from ZIF-8(CV)@PMA in water, PSF, DMEM and PBS

ZIF-8(CV)@PMA				
t(h)	water	PSF	DMEM	PBS
0	0.00 ± 0.01	0.00 ± 0.01	0.00 ± 0.01	0.00 ± 0.04
4	0.51 ± 0.01	1.54 ± 0.11	1.53 ± 0.15	1.61 ± 0.09
8	0.10 ± 0.02	1.22 ± 0.09	1.97 ± 0.13	1.92 ± 0.04
24	0.22 ± 0.01	2.51 ± 0.19	2.21 ± 0.08	2.61 ± 0.09
48	0.12 ± 0.01	0.95 ± 2.15	1.20 ± 1.15	2.91 ± 1.32
120	0.10 ± 0.01	1.92 ± 0.26	2.59 ± 0.31	2.71 ± 0.13

Table S10. % CV released from UiO-67(CV) in water, PSF, DMEM and PBS

UiO-67(CV)				
t(h)	water	PSF	DMEM	PBS
0	0.00 ± 0.02	0.00 ± 0.03	0.00 ± 0.04	0.00 ± 0.05
4	0.01 ± 0.02	1.37 ± 0.62	0.24 ± 0.07	3.32 ± 1.73
8	0.02 ± 0.02	2.18 ± 0.21	0.09 ± 0.22	2.99 ± 0.72
24	0.01 ± 0.01	2.09 ± 0.04	1.93 ± 0.30	3.94 ± 0.23
48	0.01 ± 0.01	2.67 ± 0.40	4.33 ± 1.08	3.99 ± 0.31
120	0.01 ± 0.01	2.85 ± 0.25	12.01 ± 0.12	5.68 ± 3.27

Table S11. % CV released from UiO-67(CV)@PMA in water, PSF, DMEM and PBS

UiO-67(CV)@PMA				
t(h)	water	PSF	DMEM	PBS
0	0.00 ± 0.01	0.00 ± 0.11	0.00 ± 0.10	0.00 ± 0.09
4	0.25 ± 0.06	0.18 ± 0.15	1.59 ± 0.06	1.10 ± 0.11
8	0.12 ± 0.11	0.01 ± 0.04	1.10 ± 0.14	1.49 ± 0.28
24	0.15 ± 0.04	1.93 ± 0.05	2.16 ± 0.88	3.52 ± 0.22
48	0.40 ± 0.15	1.88 ± 0.07	1.90 ± 0.50	4.86 ± 0.51
120	0.17 ± 0.12	2.08 ± 0.54	3.33 ± 0.66	6.68 ± 0.15

Table S12. % CV released from NU-1000(CV) in water, PSF, DMEM and PBS

NU-1000(CV)				
t(h)	water	PSF	DMEM	PBS
0	0.0 ± 0.3	0.0 ± 0.1	0.0 ± 0.1	0.0 ± 0.1
4	2.3 ± 0.5	1.0 ± 0.6	1.8 ± 0.1	0.0 ± 0.2
8	1.7 ± 0.5	1.0 ± 0.8	1.9 ± 0.2	0.0 ± 0.1
24	2.0 ± 0.1	1.0 ± 1.1	1.8 ± 0.1	0.1 ± 0.1
48	2.7 ± 0.5	2.0 ± 1.6	2.8 ± 0.1	0.3 ± 0.1
120	3.5 ± 0.5	3.1 ± 0.5	3.1 ± 0.5	0.1 ± 0.1

Table S13. % CV released from NU-1000(CV)@PMA in water, PSF, DMEM and PBS

NU-1000(CV)@PMA				
t(h)	water	PSF	DMEM	PBS
0	0.0 ± 0.02	0.0 ± 0.06	0.00 ± 0.32	0.00 ± 0.08
4	0.01 ± 0.02	0.74 ± 0.07	0.69 ± 0.21	0.22 ± 0.04
8	0.12 ± 0.04	0.39 ± 0.03	0.31 ± 0.21	0.12 ± 0.04
24	0.01 ± 0.04	0.28 ± 0.07	0.39 ± 0.18	0.11 ± 0.03
48	0.01 ± 0.1	0.09 ± 0.07	0.04 ± 0.12	0.01 ± 0.02
120	0.04 ± 0.02	0.07 ± 0.03	0.04 ± 0.15	0.03 ± 0.01

Table S14. % CV released from PCN-222(CV) in water, PSF, DMEM and PBS

PCN-222(CV)				
t(h)	water	PSF	DMEM	PBS
0	0.34 ± 0.07	0.04 ± 0.01	0.05 ± 0.05	0.03 ± 0.01
4	2.78 ± 0.38	0.05 ± 0.01	0.08 ± 0.01	0.04 ± 0.01
8	3.08 ± 0.45	3.87 ± 0.03	0.54 ± 0.02	0.06 ± 0.02
24	5.21 ± 0.59	5.03 ± 0.06	0.77 ± 0.74	0.07 ± 0.01
48	5.16 ± 0.09	5.05 ± 0.01	0.87 ± 0.82	0.02 ± 0.01
120	6.21 ± 0.59	6.03 ± 0.06	0.77 ± 0.73	0.07 ± 0.01

Table S15. % CV released from PCN-222(CV)@PMA in water, PSF, DMEM and PBS

PCN-222(CV)@PMA				
t(h)	water	PSF	DMEM	PBS
0	0.17 ± 0.02	0.58 ± 0.03	0.04 ± 0.02	0.24 ± 0.01
4	0.13 ± 0.02	0.51 ± 0.02	0.52 ± 0.23	0.24 ± 0.03
8	0.13 ± 0.02	0.51 ± 0.02	0.52 ± 0.23	0.24 ± 0.03
24	0.15 ± 0.02	0.68 ± 0.07	0.67 ± 0.16	0.33 ± 0.01
48	0.17 ± 0.02	0.77 ± 0.04	0.58 ± 0.04	0.44 ± 0.03
120	0.15 ± 0.02	0.28 ± 0.01	0.64 ± 0.10	0.41 ± 0.03

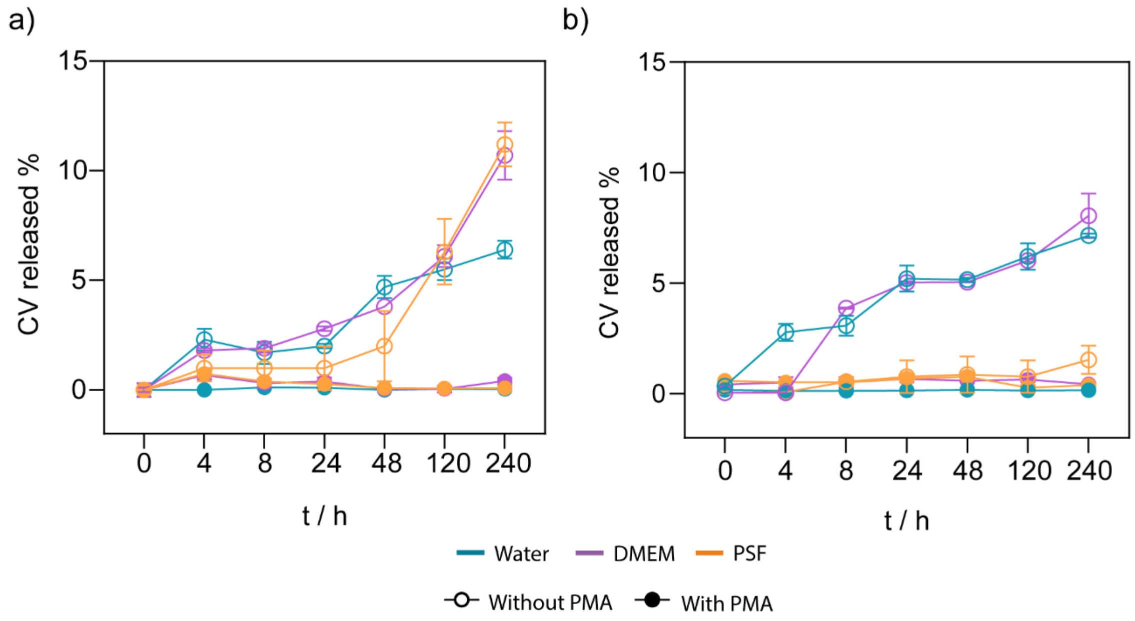

Figure S18: % CV released with respect to the loaded quantity measured by fluorescent measurements of the SN in water, PSF and cell medium up to 10 days for a) NU-1000 and b) PCN-222, with (solid dots) and without PMA (empty dots).

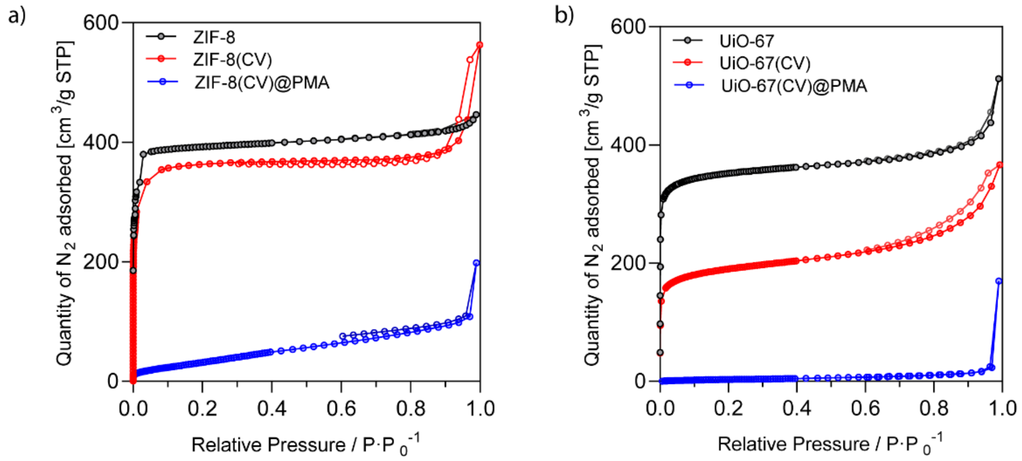

Figure S19: N_2 adsorption and desorption isotherms of a) ZIF-8 and b) UiO-67 samples.

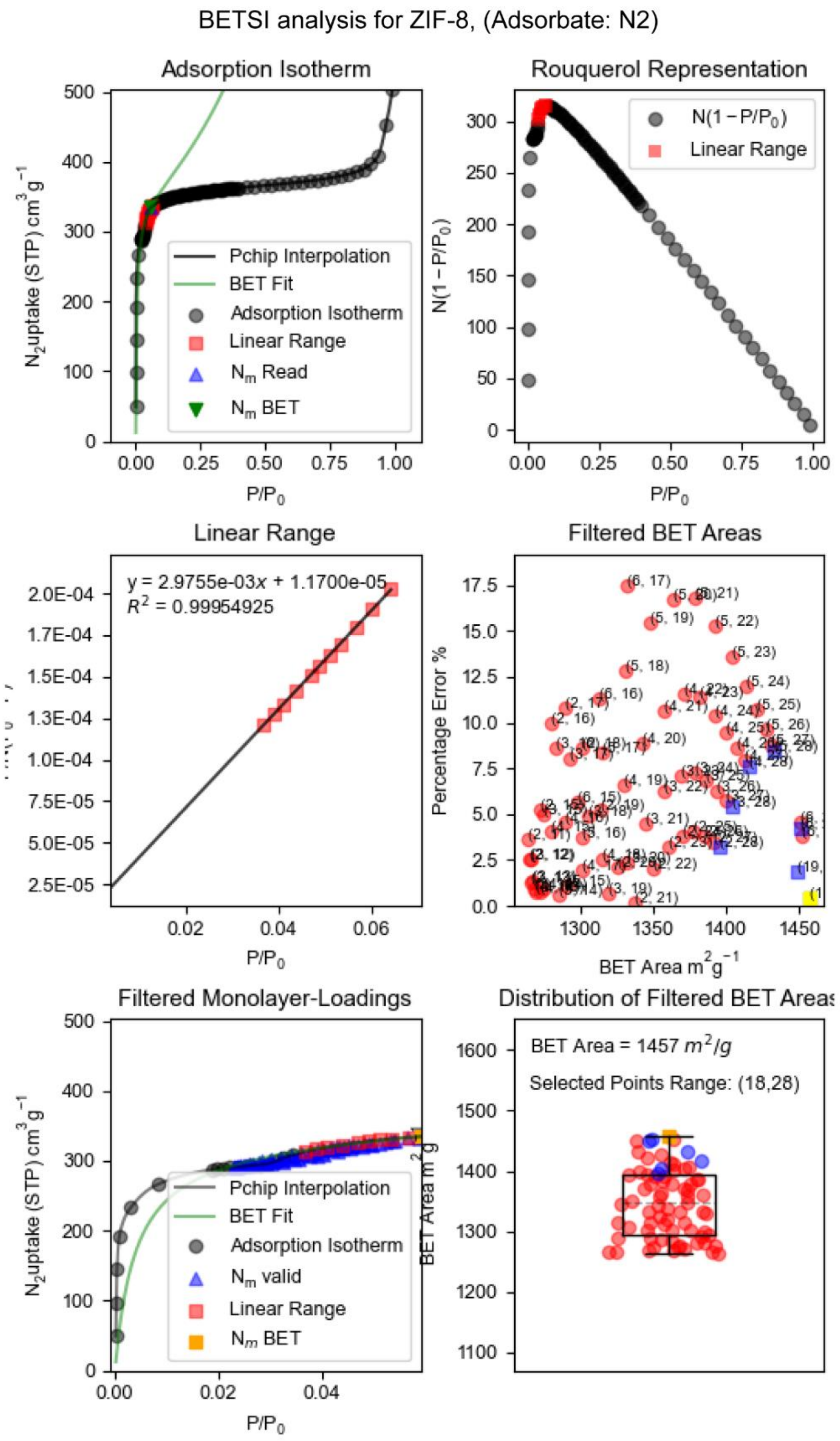

Figure S20: BETSI analysis of ZIF-8.

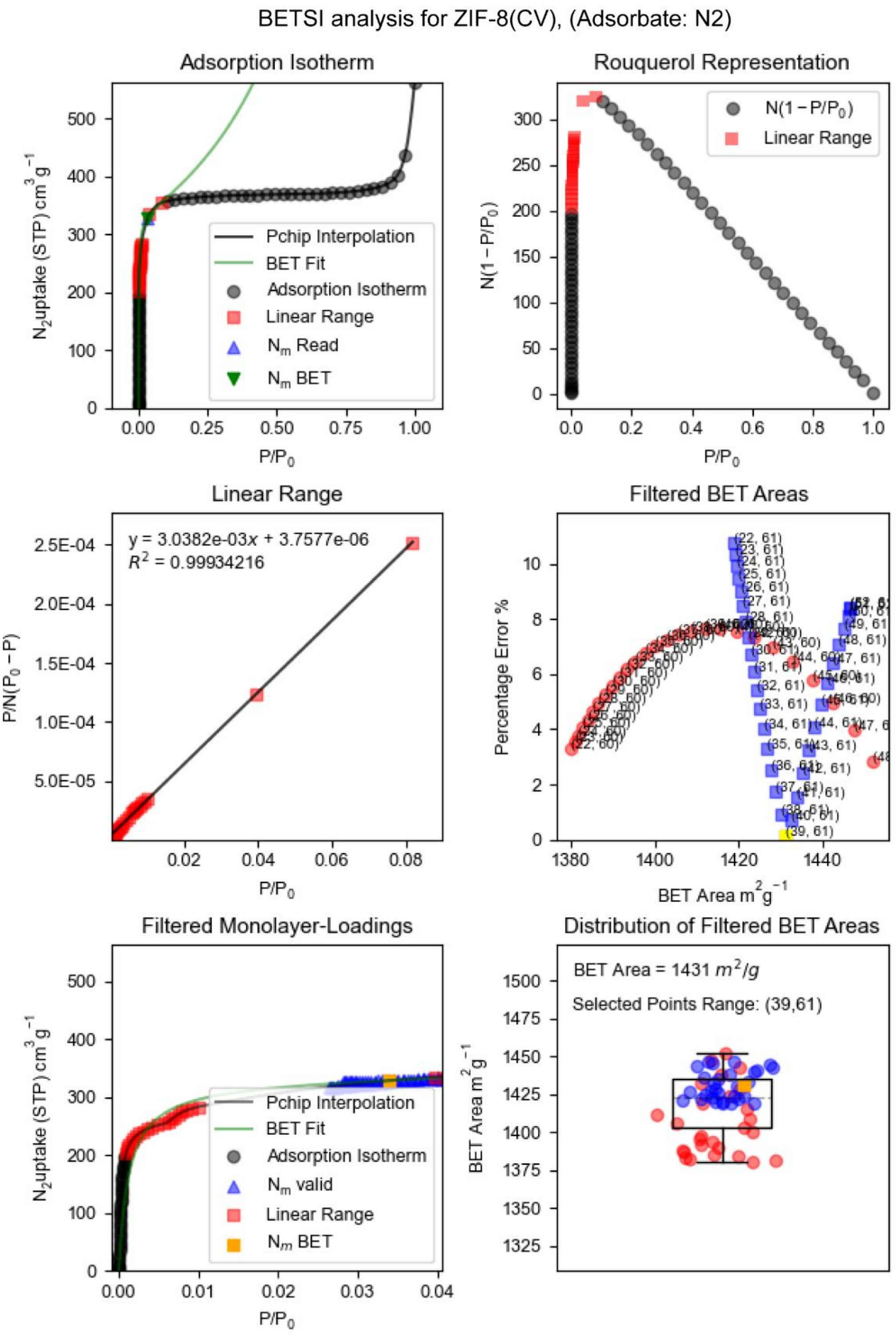

Figure S21: BETSI analysis of ZIF-8(CV).

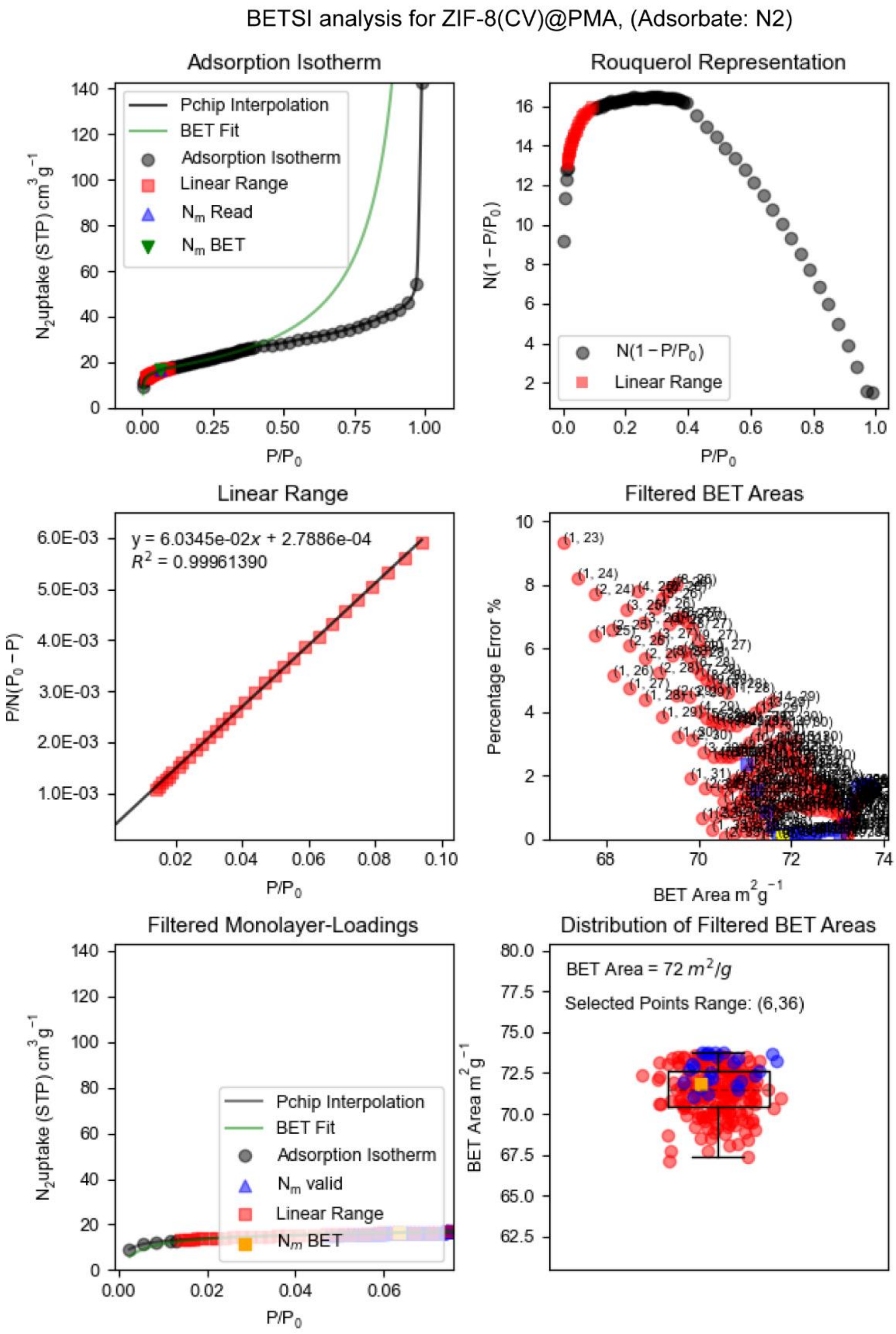

Figure S22: BETSI analysis of ZIF-8(CV)@PMA.

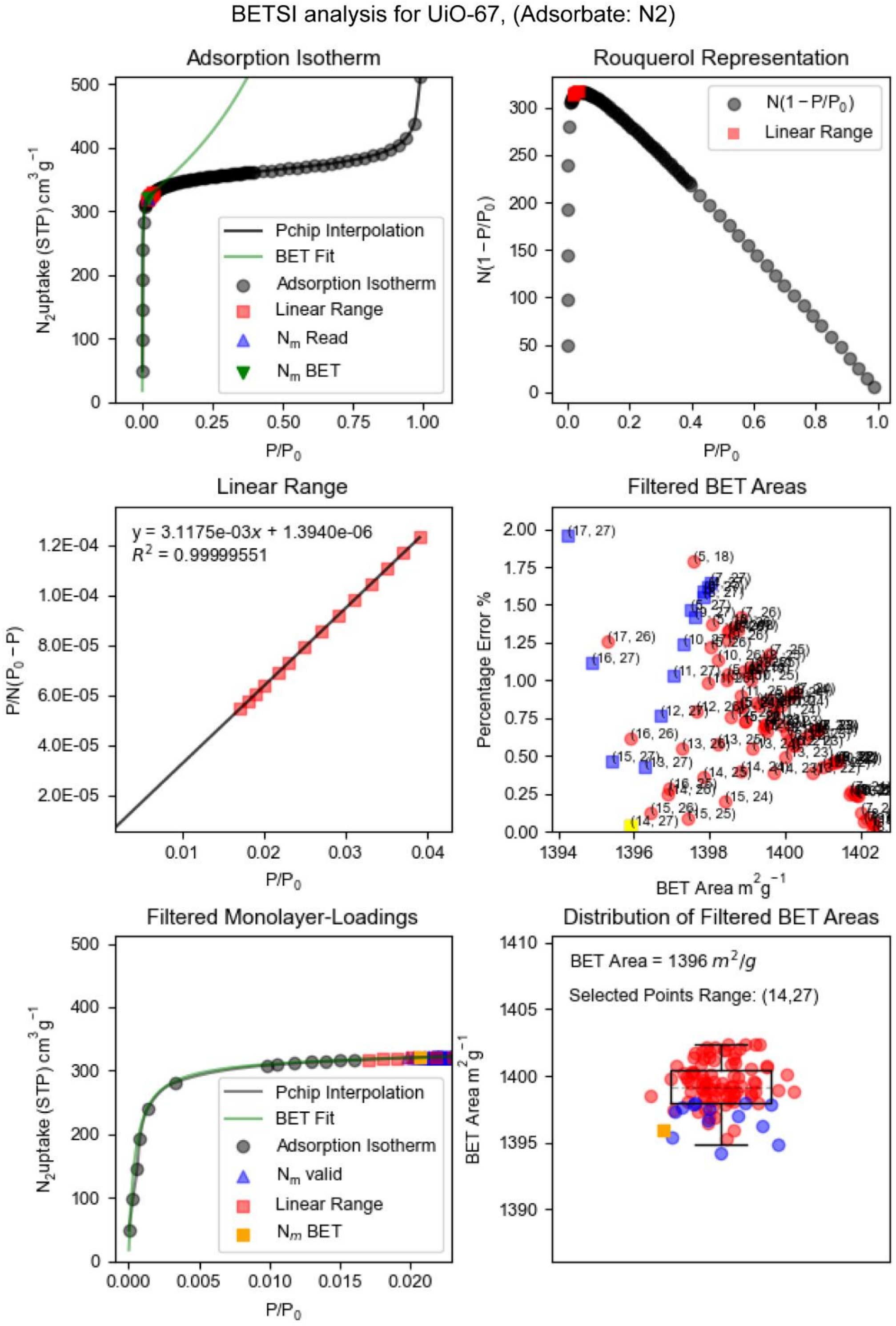

Figure S23: BETSI analysis of UiO-67.

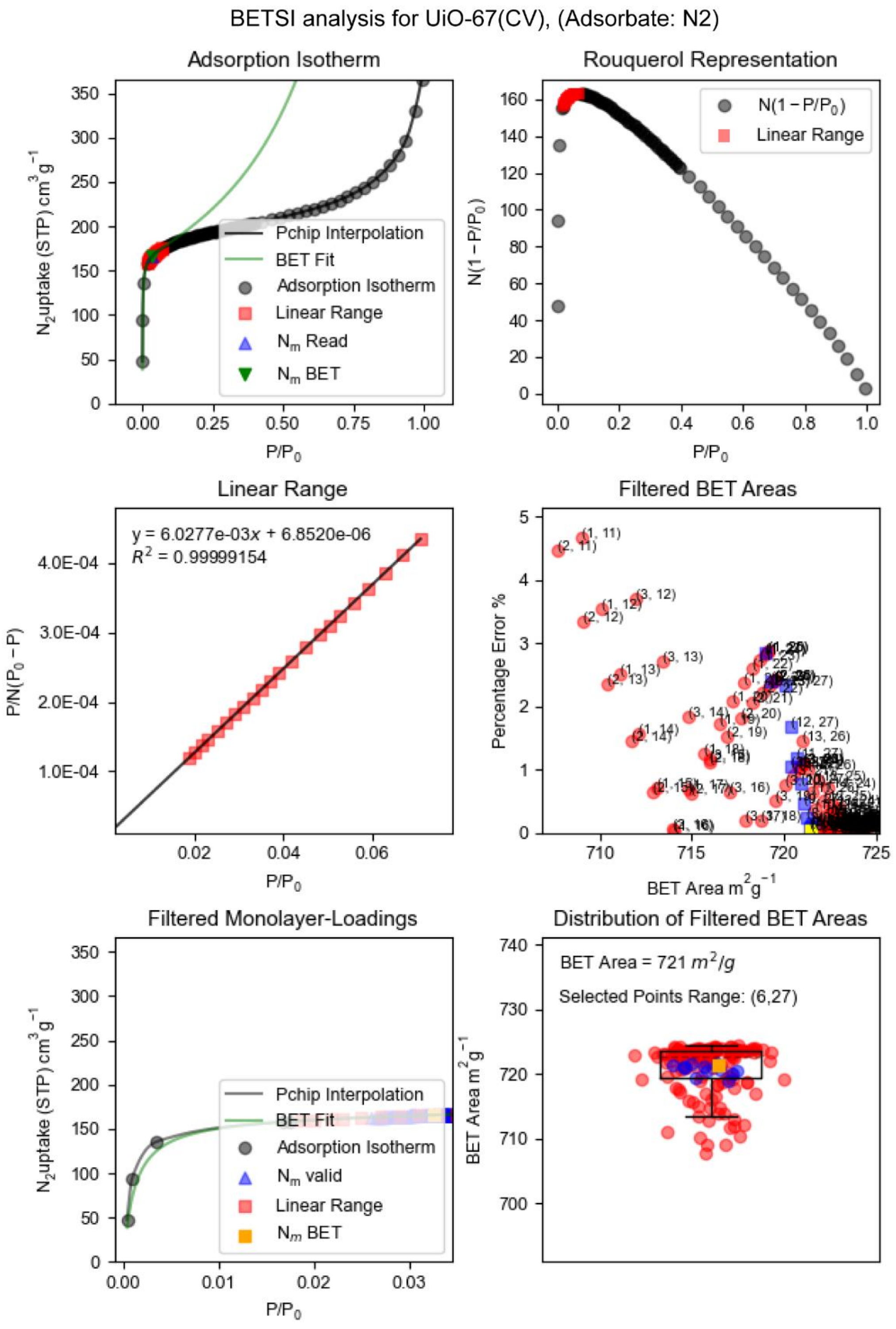

Figure S24: BETSI analysis of UiO-67(CV).

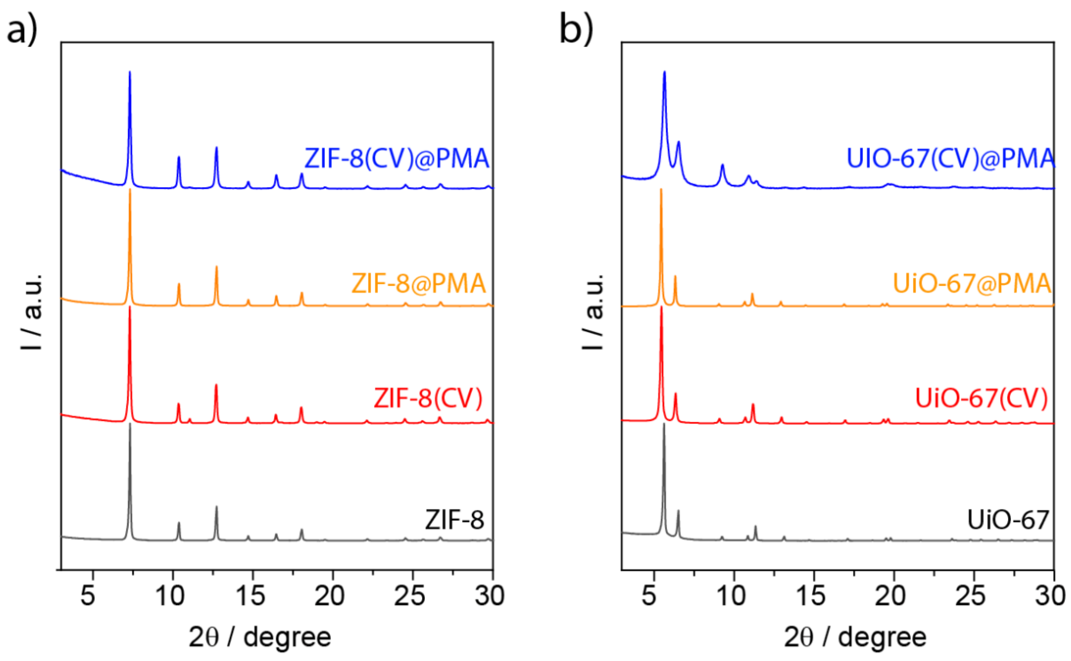

Figure S25: PXRD patterns of a) ZIF-8 and b) UiO-67 samples before and after the CV loading and PMA functionalization.

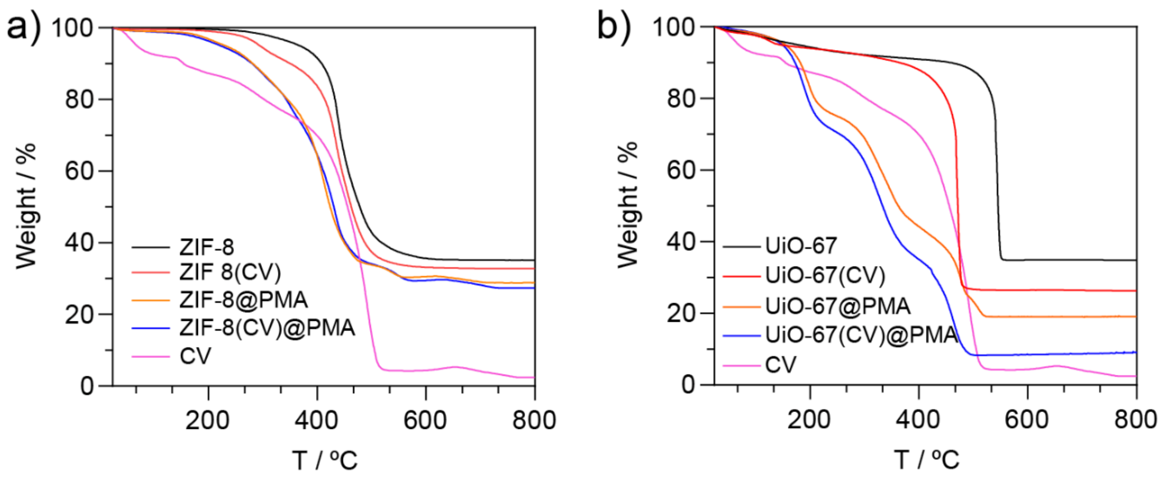

Figure S26: TGA measurements of a) ZIF-8 and b) UiO-67 prepared samples.

Recalculation of the Inorganic and Organic Content

Considering the evaporation of water and remaining solvents within the porosity T<150 °C, the recalculation of the inorganic and organic content (% wt = weight percentage) of the samples (*i.e.*, ZIF-8, ZIF-8(CV), ZIF-8@PMA, ZIF-8(CV)@PMA and the UiO-67 equivalents), is described below (numerical values are provided in Table 12):

- CV wt% NMOFs: considering the inorganic content (Zn) remains constant:

$$\text{Organic wt\% NMOF}_{\text{equivalent}} = \frac{\text{Inorganic wt\% NMOF(CV)}}{\text{Inorganic wt\% NMOF}} \text{x Organic wt\% NMOF}$$

$$\text{CV \%} = \text{Total organic wt\% NMOF(CV)} - \text{Organic wt\% NMOF(CV)}_{\text{equivalent}}$$

- PMA wt% in NMOFs-PMA**:** considering the inorganic content (Zn) remains constant:

$$\text{Organic wt\% NMOF@PMA}_{\text{equivalent}} = \frac{\text{Inorganic wt\% NMOF@PMA}}{\text{Inorganic wt\% NMOF}} \text{x Organic wt\% NMOF}$$

$$\text{PMA wt \%} = \text{Total organic wt\% NMOF@PMA} - \text{Organic wt\% NMOF@PMA}_{\text{equivalent}}$$

- CV & PMA wt% in NMOFs(CV)@PMA: considering the inorganic content remains constant and taking the values obtained from fluorescence measurements as the % of CV that remains inside after PMA functionalization:

$$\text{Organic wt\% NMOF(CV)@PMA}_{\text{equivalent}} = \frac{\text{Inorganic wt\% NMOF(CV)@PMA}}{\text{Inorganic wt\% NMOF}} \text{x Organic wt\% NMOF}$$

$$(\text{PMA} + \text{CV}) \text{ wt \%} = \text{Total organic wt\% NMOF(CV)@PMA} - \text{Organic wt\% NMOF(CV)@PMA}_{\text{equivalent}}$$

$$\text{CV wt \%} = (\%CV\ fluo) \frac{\text{Inorganic wt\% NMOF(CV)@PMA}}{\text{Inorganic wt\% NMOF}} \text{x CV wt\% in NMOF(CV)}$$

$$\text{PMA wt \%} = (\text{PMA} + \text{CV}) \text{ wt\%} - \text{CV wt\%}$$

Table S16. TGA Measurements of ZIF-8 NMOFs

	% Inorganic	% Organic	wt % (CV)	wt % (PMA)
ZIF-8	35.2	64.8	n/a	n/a
ZIF-8@PMA	28.8	71.2	n/a	12.9
ZIF-8(CV)	30.38	69.62	9.5	n/a
ZIF-8(CV)@PMA	25.38	74.62	-	-
CV	2.42	97.58	n/a	n/a

Table S17. TGA Measurements of UiO-67 NMOFs

	% Inorganic	% Organic	wt % (CV)	wt % (PMA)
UiO-67	34.80	65.20	n/a	n/a
UiO-67@PMA	19.10	80.90	n/a	36.5
UiO-67(CV)	23.89	76.11	23.9	n/a
UiO-67(CV)@PMA	6.64	93.36	-	-
CV	2.42	97.58	n/a	n/a

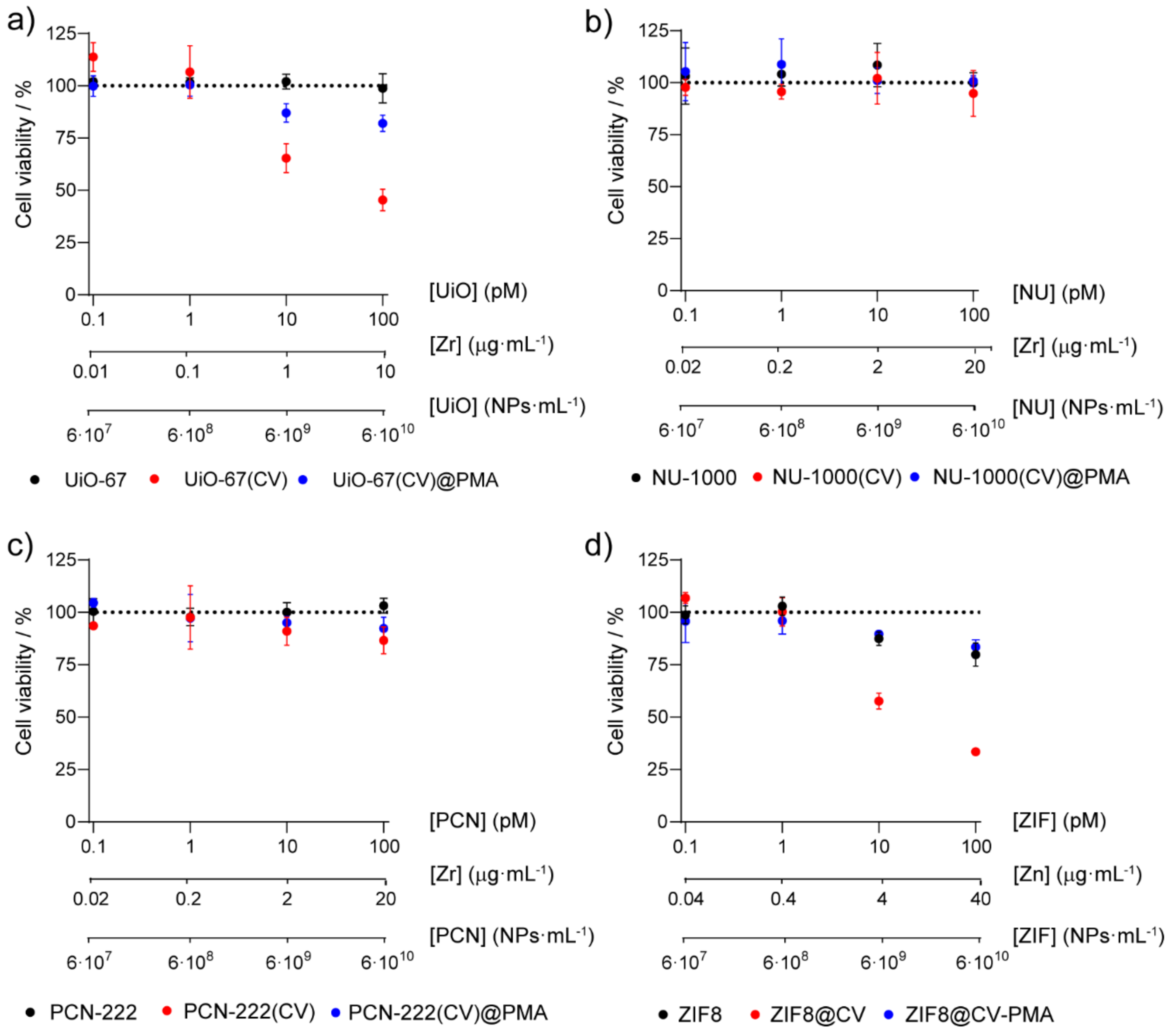

Figure S27: Cell viability of A549 cells incubated with a) UiO-67, b) NU-1000, c) PCN-222 and d) ZIF-8 nanoparticles for 24 h, using MTT assay.

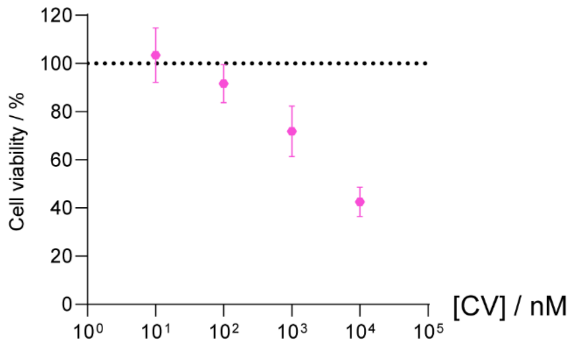

Figure S28. Cell viability of A459 cells incubated with CV for 24 h assessed by MTT assay.

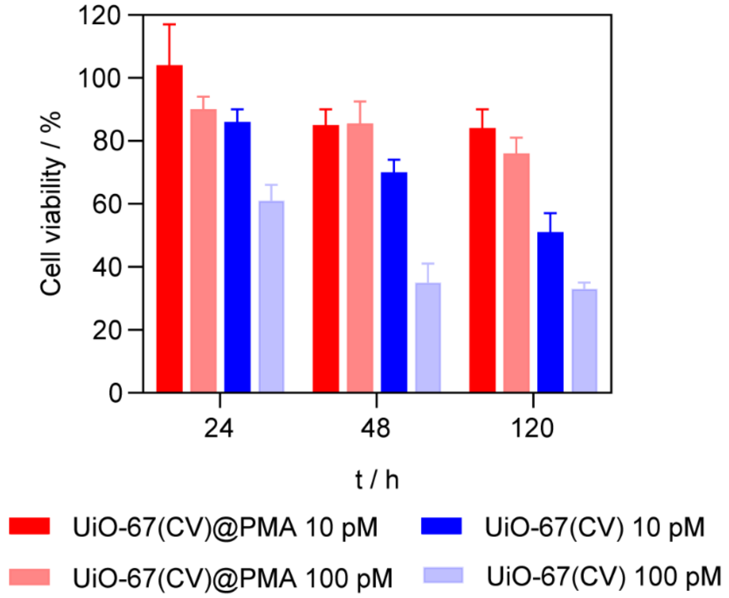

Figure S29. Cell viability of A459 cells incubated with UiO-67(CV) and UiO-67(CV)@PMA by MTT assay.

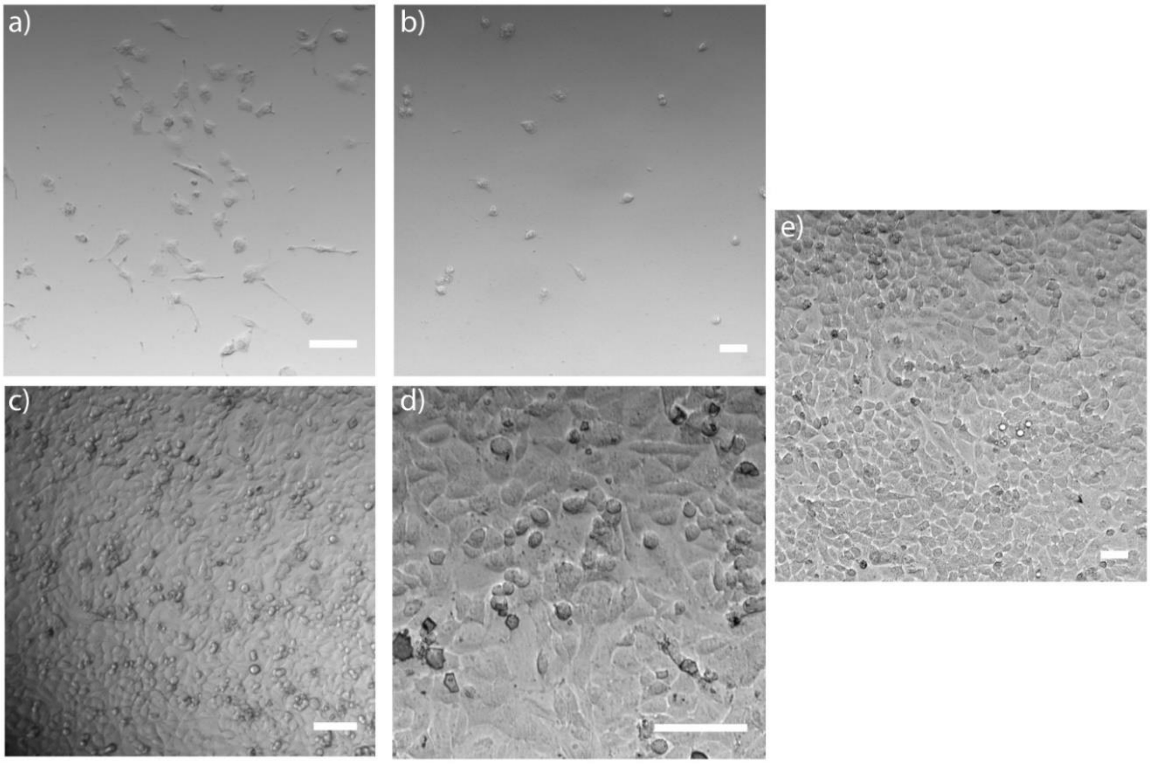

Figure S30. Microscopy images of A459 cells incubated for 5 days with a) UiO-67(CV) 10 pM, b) UiO-67(CV) 100 pM, c) UiO-67(CV)@PMA 10 pM and d) UiO-67(CV)@PMA 100 pM and e) Control cells. Scale bars 50 µm.

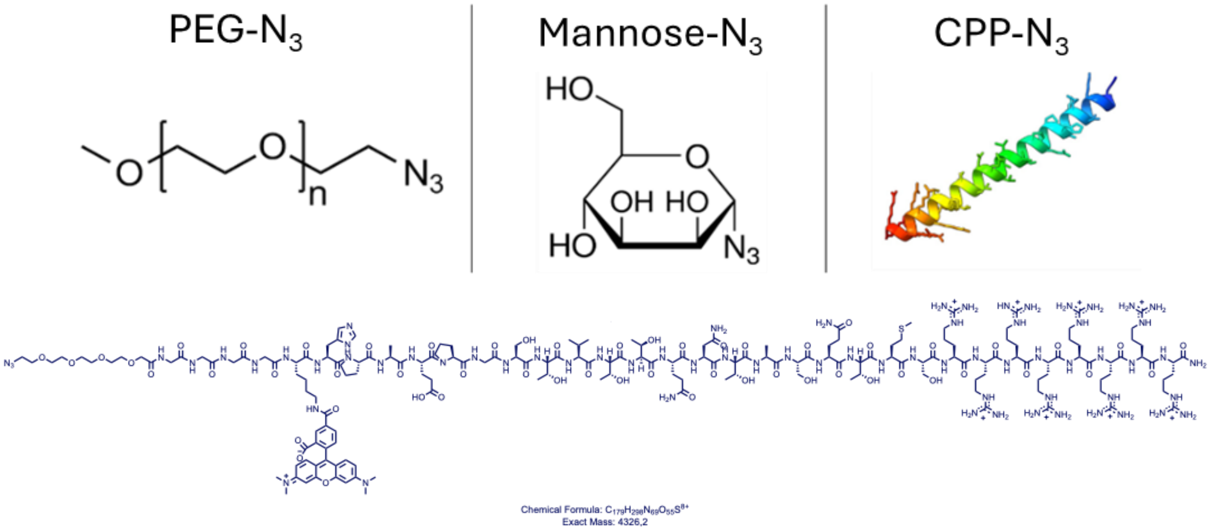

Figure S31: Selected azide-functionalized biomolecules for the functionalization of the NMOFs.

Table S18. Molar weight and selected quantities for the chosen biomolecules

Dye	MW (g·mol^{-1})	molecules/ mol MOF
PEG	5000	10000
Mannose	430	17000
CPP	3907	4000

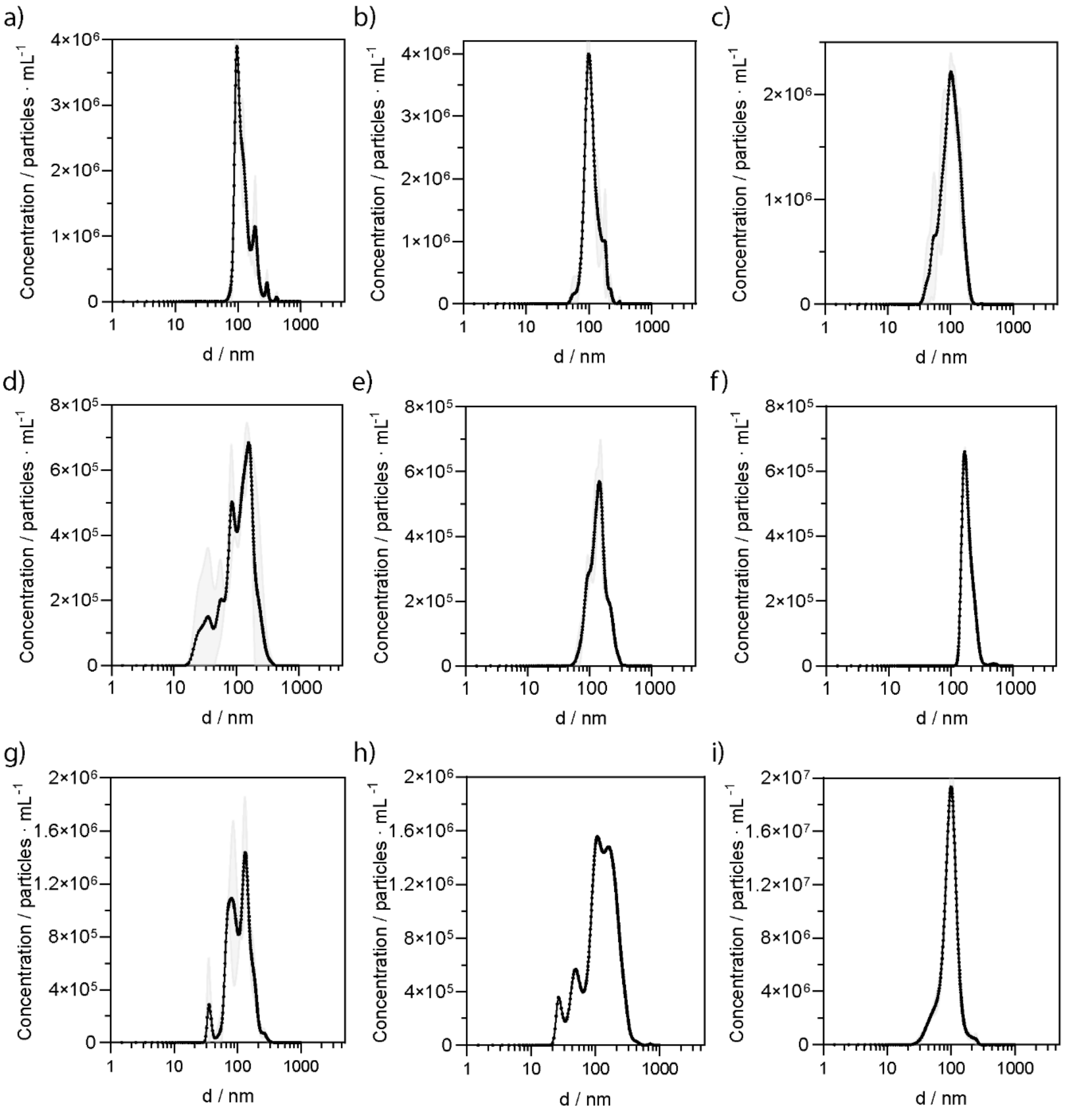

Figure S32: NTA measurements of the nanoparticle diameter (nm) for a) Z b) Z@PEG, c) Z@Man, d) Z@CPP, e) U (Without PMA), f) U, g) U@PEG, h) U@Man, i) U@PEG.

Table S19. Mean size d_h (in nm) from the number (N) and intensity (I) distribution from DLS and Zeta potential results for ZIF-8(CV)@DPMA (Z) samples

Sample	N ± sd	I ± sd	V ± sd	PDI	ζ-pot (mV)
Z	158 ± 6	195 ± 7	194 ± 7	0.05	-23 ± 6
Z@PEG	155 ± 8	184 ± 5	183 ± 5	0.05	-19 ± 5
Z@Man	154 ± 14	203 ± 19	202 ± 21	0.20	-17 ± 5
Z@CPP	162 ± 6	199 ± 10	198 ± 6	0.08	-18 ± 5

Table S20. Mean size d_h (in nm) from the number (N) and intensity (I) distribution from DLS and Zeta potential results for UiO-67(CV)@DPMA (U) samples

Sample	N ± sd	I ± sd	V ± sd	PDI	ζ-pot (mV)
U	162 ± 9	195 ± 7	194 ± 7	0.05	-24 ± 6
U@PEG	169 ± 7	196 ± 7	190 ± 5	0.02	-2 ± 6
U@Man	159 ± 10	187 ± 3	186 ± 4	0.05	-3 ± 4
U@CPP	163 ± 6	192 ± 10	192 ± 7	0.02	2 ± 15

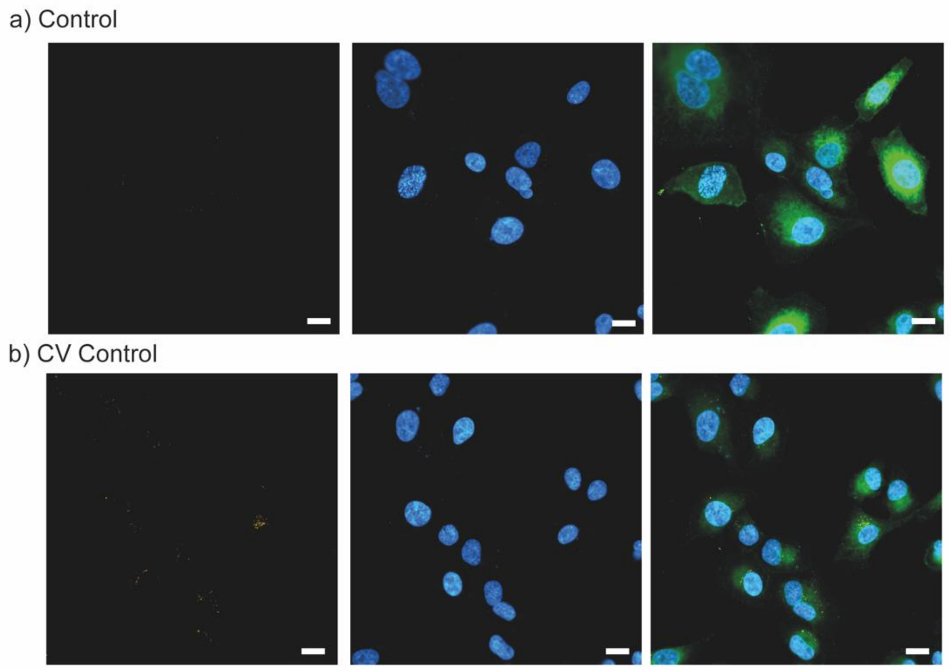

Figure S33: Confocal microscopy images of A549 cells control (a) and A549 incubated with CV concentration comparable with the loaded into the NMOFs (100 nM) (b). Left column (yellow) corresponds to CV fluorescence channel, central column corresponds to DAPI, and right column to the merged channels CV + DAPI + cell mask. Images acquired by Leica Thunder microscope (channels: DAPI (ex. 391/32, em. 473/22), cell mask (em. 478/33, ex. 519/25), and CV (ex. 578/24, em. 641/78). Scale bars: 20 μm.

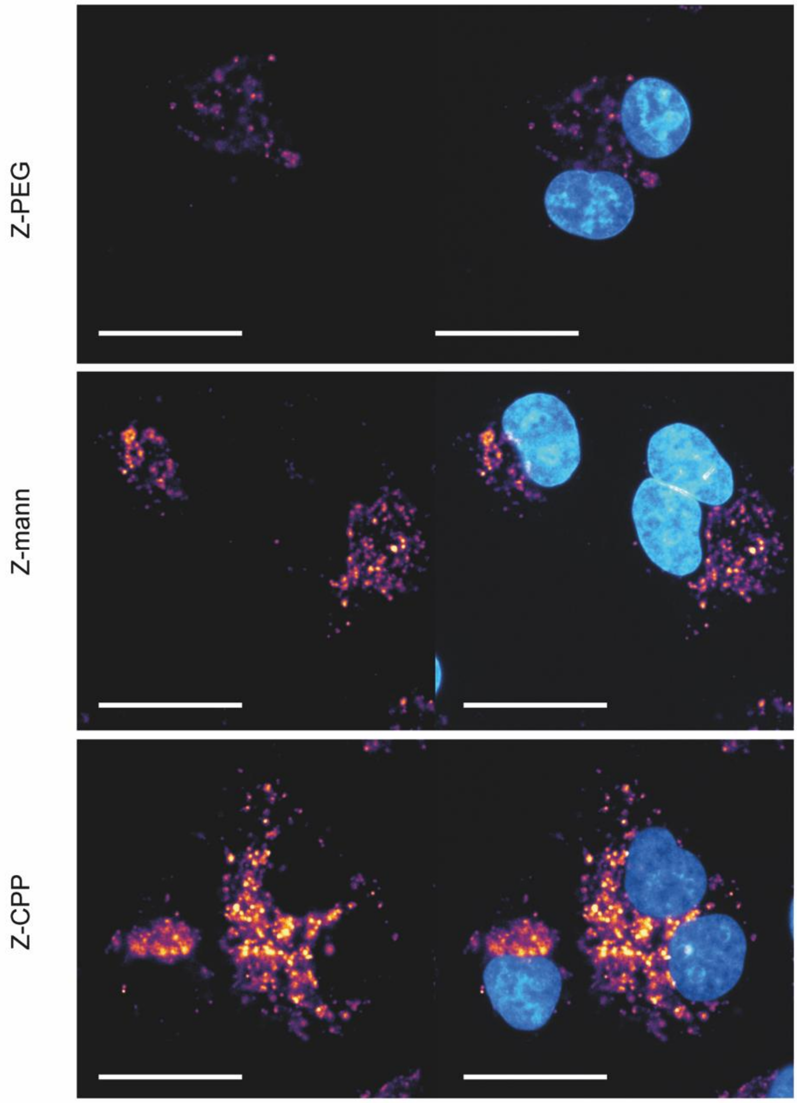

Figure S34: Thunder Leica microscopy images (computational clearance) of A549 cells incubated with ZIF-8 with different surface functionalizations (PEG, mannose, and CPP) for 3 h at a concentration of 0.5 pM. The left column (magenta) corresponds to CV fluorescence, and the right column corresponds to the merged channels of CV and DAPI. Images acquired by Thunder Leica microscope (63x objective; channels: DAPI (ex. 391/32, em. 473/22) and CV (ex. 578/24, em. 641/78). Scale bars: 20 μm.

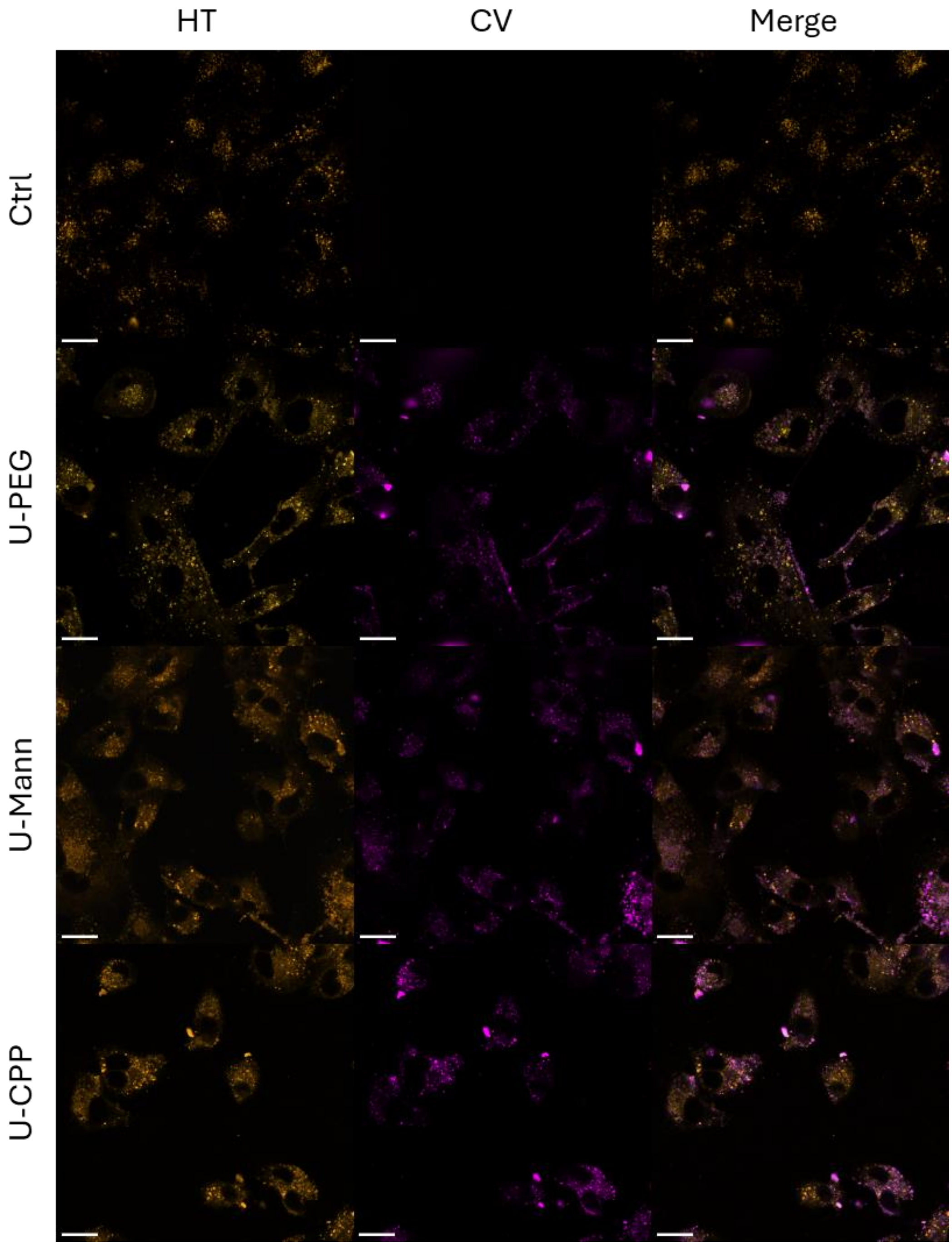

Figure S35. A549 cells incubated overnight with 0.5 pM of UiO-67 with different surface functionalizations (U-PEG, U-Mann and U-CPP). Images were acquired on a Leica Stellaris 8 confocal/STED microscope with a 93x/1.30 GLYC objective (HC PL APO CS2). Human Transferrin CF®543 (orange) (Biotium, Cat#00082, reconstituted 1x PBS to a stock 1 mg/mL, for culture a 1:40 dilution in cell media was used for 40 min) imaged in confocal mode excited with 543 nm laser line, detection at 548–610 nm (HyD S 2), pinhole 1 AU and Cresyl violet (magenta) excited 610 nm line detection at 620–771 nm (HyD X 4) with STED (775 nm depletion laser, 1.057 W pulsed, Vortex 780 phase filter). Scale bars: 20 µm.

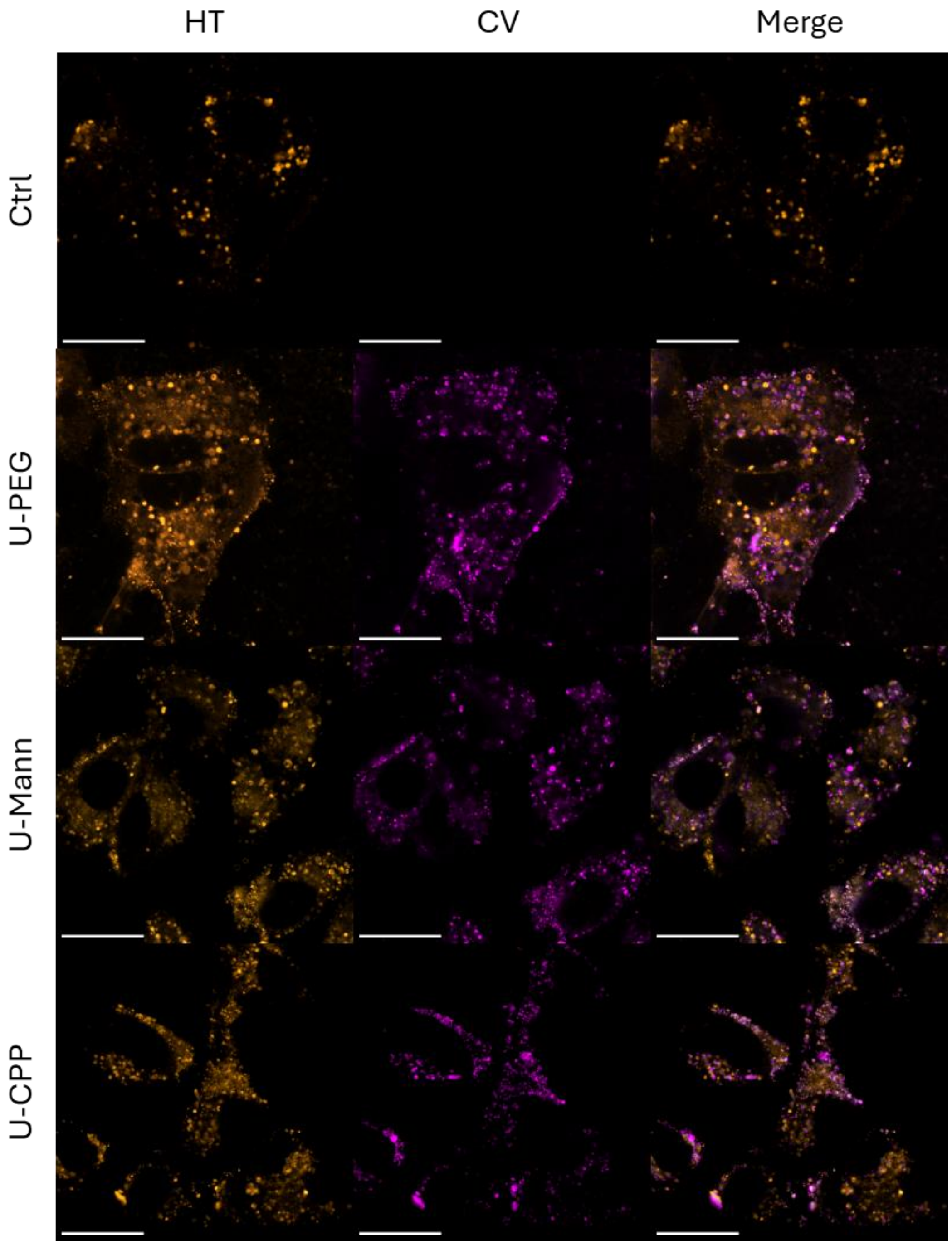

Figure S36. A549 cells incubated overnight with 0.5 pM UiO-67 with different surface functionalization (U-PEG, U-Mann and U-CPP). Images were acquired on a Leica Stellaris 8 confocal/STED microscope with a 93x/1.30 GLYC objective (HC PL APO CS2) (zoom 1.95×) for high-resolution detail. Human Transferrin CF®543 (orange) (Biotium, Cat#00082, reconstituted 1x PBS to a stock 1 mg/mL, for culture a 1:40 dilution in cell media was used for 40 min) imaged in confocal mode excited with 543 nm laser line, detection at 548–610 nm (HyD S 2), pinhole 1 AU and Cresyl violet (magenta) excited 610 nm line detection at 620–771 nm (HyD X 4) with STED (775 nm depletion laser, 1.057 W pulsed, Vortex 780 phase filter). Scale bars: 20 µm**.**

Table S21. Co-localization analysis was performed using *JaCoP* ImageJ plugin, quantifying Pearson's coefficient (*R*, ranging -1 to 1), overlap coefficient (r, ranging 0 to 1), and Mander's coefficients (M_1, M_2, ranging 0 to 1), where M_1 represents the fraction of Human Transferrin (endosome) overlapping with cresyl violet (U), and M_2 the fraction of cresyl violet (U) overlapping with Human Transferrin (endosome). R = 0.5-0.6 indicates a moderate co-localization between Human Transferrin (endosome) and cresyl violet (U-X= U-PEG, U-Mann & U-CPP), suggesting a partial U-X accumulation in endosomal compartments. On the other hand, an overlap coefficient (r) value of 0.8 indicates a strong coincidence with endosomal trafficking. $M_1 \approx 0.3$ indicates that U-X are partially interacting with endosomes, while an $M_2 \approx 0.5$ indicates that half of the trafficking of U-X is endosome-associated (likely early in the uptake process).

Sample	Ctrl	U-PEG	U-Mann	U-CPP
R (Pearson's Coefficient)	0.065	0.545	0.581	0.598
r (Overlap Coefficient)	0.000	0.834	0.865	0.846
Manders' Coefficients:				
M1	0.000	0.310	0.341	0.373
M2	0.000	0.568	0.578	0.548

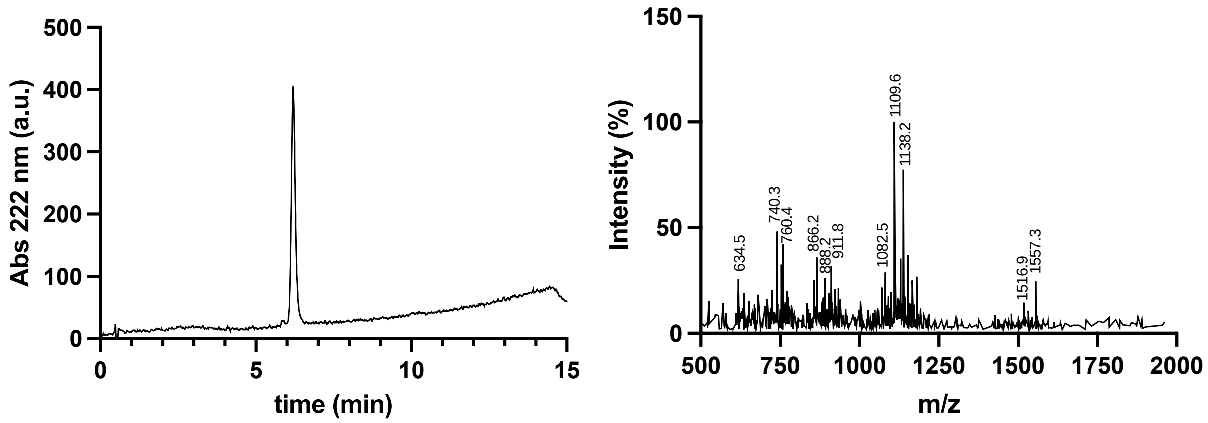

Figure S37. RP-HPLC [Agilent SB-C18 column, H_2O (0.1% TFA)/ CH_3CN (0.1% TFA) 95:5 →5:95 (0→12 min)] (retention time 6.2 min) and ESI-MS for CPP-N_3.

1. Melle, F.; Menon, D.; Conniot, J.; Ostolaza-Paraiso, J.; Mercado, S.; Oliveira, J.; Chen, X.; Mendes, B. B.; Conde, J.; Fairen-Jimenez, D., Rational Design of Metal–Organic Frameworks for Pancreatic Cancer Therapy: from Machine Learning Screening to In Vivo Efficacy. *Adv. Mater.* **2025,** *Early View* (10.1002/adma.202412757), 2412757.
2. Ceballos, M.; Funes‐Hernando, S.; Zampini, G.; Cedrún‐Morales, M.; Vila‐Fungueiriño, J. M.; Pelaz, B.; del Pino, P., Seeded‐Growth of PCN‐224 onto Plasmonic Nanoparticles: Photoactive Microporous Nanocarriers. *Small Str.* **2024**, 2300464.

3. Zimpel, A.; Preiß, T.; Röder, R.; Engelke, H.; Ingrisch, M.; Peller, M.; Rädler, J. O.; Wagner, E.; Bein, T.; Lächelt, U.; Wuttke, S., Imparting Functionality to MOF Nanoparticles by External Surface Selective Covalent Attachment of Polymers. *Chem. Mater.* **2016,** *28* (10), 3318-3326.
4. Chen, X.; Mendes, B. B.; Zhuang, Y.; Conniot, J.; Mercado Argandona, S.; Melle, F.; Sousa, D. P.; Perl, D.; Chivu, A.; Patra, H. K.; Shepard, W.; Conde, J.; Fairen-Jimenez, D., A Fluorinated BODIPY-Based Zirconium Metal–Organic Framework for In Vivo Enhanced Photodynamic Therapy. *J. Am. Chem. Soc.* **2024,** *146* (2), 1644-1656.
5. Wang, S.; Chen, Y.; Wang, S.; Li, P.; Mirkin, C. A.; Farha, O. K., DNA-functionalized metal–organic framework nanoparticles for intracellular delivery of proteins. *J. Am. Chem. Soc.* **2019,** *141* (6), 2215-2219.
6. Chen, X.; Zhuang, Y.; Rampal, N.; Hewitt, R.; Divitini, G.; O'Keefe, C. A.; Liu, X.; Whitaker, D. J.; Wills, J. W.; Jugdaohsingh, R.; Powell, J. J.; Yu, H.; Grey, C. P.; Scherman, O. A.; Fairen-Jimenez, D., Formulation of Metal–Organic Framework-Based Drug Carriers by Controlled Coordination of Methoxy PEG Phosphate: Boosting Colloidal Stability and Redispersibility. *J. Am. Chem. Soc.* **2021,** *143* (34), 13557-13572.
7. Zheng, X.; Wang, L.; Guan, Y.; Pei, Q.; Jiang, J.; Xie, Z., Integration of metal-organic framework with a photoactive porous-organic polymer for interface enhanced phototherapy. *Biomaterials* **2020,** *235*, 119792.
8. Zheng, X.; Wang, L.; Pei, Q.; He, S.; Liu, S.; Xie, Z., Metal–Organic Framework@Porous Organic Polymer Nanocomposite for Photodynamic Therapy. *Chem. Mater.* **2017,** *29* (5), 2374-2381.
9. Chen, L.-J.; Zhao, X.; Liu, Y.-Y.; Yan, X.-P., Macrophage membrane coated persistent luminescence nanoparticle@MOF-derived mesoporous carbon core–shell nanocomposites for autofluorescence-free imaging-guided chemotherapy. *J. Mater. Chem. B* **2020,** *8* (35), 8071-8083.
10. Adhikari, C.; Mishra, A.; Nayak, D.; Chakraborty, A., Metal organic frameworks modified mesoporous silica nanoparticles (MSN): A nano-composite system to inhibit uncontrolled chemotherapeutic drug delivery from Bare-MSN. *J. Drug Deliv. Sci. Tec.* **2018,** *47*, 1-11.
11. Chen, Y.; Wu, H.; Yang, T.; Zhou, G.; Chen, Y.; Wang, J.; Mao, C.; Yang, M., Biomimetic Nucleation of Metal–Organic Frameworks on Silk Fibroin Nanoparticles for Designing Core–Shell-Structured pH-Responsive Anticancer Drug Carriers. *ACS Appl. Mater. Interfaces* **2021,** *13* (40), 47371-47381.
12. Hu, L.; Xiong, C.; Wei, G.; Yu, Y.; Li, S.; Xiong, X.; Zou, J.-J.; Tian, J., Stimuli-responsive charge-reversal MOF@polymer hybrid nanocomposites for enhanced co-delivery of chemotherapeutics towards combination therapy of multidrug-resistant cancer. *J. Colloid Interface Sci.* **2022,** *608*, 1882-1893.
13. Migliavacca, M.; Cedrún-Morales, M.; Ceballos, M.; Soprano, E.; Polo, E.; Pelaz, B.; Pino, P. d., Engineered cell membrane-cloaked metal-organic framework nanocrystals for intracellular cargo delivery. *J. Colloid Interface Sci.* **2025,** *682*, 31-40.
14. Illes, B.; Hirschle, P.; Barnert, S.; Cauda, V.; Wuttke, S.; Engelke, H., Exosome-Coated Metal–Organic Framework Nanoparticles: An Efficient Drug Delivery Platform. *Chem. Mater.* **2017,** *29* (19), 8042-8046.
15. Alyami, M. Z.; Alsaiari, S. K.; Li, Y.; Qutub, S. S.; Aleisa, F. A.; Sougrat, R.; Merzaban, J. S.; Khashab, N. M., Cell-Type-Specific CRISPR/Cas9 Delivery by Biomimetic Metal Organic Frameworks. *J. Am. Chem. Soc.* **2020,** *142* (4), 1715-1720.
